# Photo-editable macromolecular information

**DOI:** 10.1038/s41467-019-11566-2

**Published:** 2019-09-04

**Authors:** Niklas Felix König, Abdelaziz Al Ouahabi, Laurence Oswald, Roza Szweda, Laurence Charles, Jean-François Lutz

**Affiliations:** 1 Université de Strasbourg, CNRS, Institut Charles Sadron UPR22, 23 rue du Loess, 67034 Strasbourg Cedex 2, France; 2 Aix Marseille Université, CNRS, UMR 7273, Institute of Radical Chemistry, 13397 Marseille Cedex 20, France

**Keywords:** Polymer characterization, Polymer synthesis

## Abstract

Light-induced alteration of macromolecular information plays a central role in biology and is known to influence health, aging and Darwinian evolution. Here, we report that light can also trigger sequence variations in abiotic information-containing polymers. Sequence-coded poly(phosphodiester)s were synthesized using four phosphoramidite monomers containing either photo-sensitive or photo-inert substituents. These monomers allow different sequence manipulations. For instance, using two light-cleavable monomers containing *o*-nitrobenzyl ether and *o*-nitroveratryl ether motifs, photo-erasable digital polymers were prepared. These polymers can be decoded by tandem mass spectrometry but become unreadable after UVA exposure. The opposite behavior, i.e. photo-revealable sequences, was obtained with polymers made of two isobaric monomers containing light-cleavable *o*-nitrobenzyl ether and light-inert *p*-nitrobenzyl ether substituents. Furthermore, when the latter two monomers were used in conjunction with a third monomer bearing a light-inert OH group, site-directed photo-mutations were induced in synthetic polymers. This was used herein to change the meaning of binary sequences.

## Abstract (web summary)

Light-induced alteration of macromolecular information plays a central role in biology and is known to influence health and aging. Here the authors show that light can trigger sequence variations in abiotic information-containing polymers that possess photo-sensitive monomers

## Introduction

Information-containing macromolecules are molecularly uniform polymers, in which a defined sequence of monomers is used to store a message: for example, a genetic or a digital sequence^1–3^. In recent years, it was shown that intentional information can be stored in biological^4^ and abiological polymers^5–11^. Such polymers are promising for applications in anti-counterfeiting, traceability, cryptography, and data storage technologies^2^. However, until now, only the synthesis (i.e. the writing method) and the sequencing (i.e. the reading method) of these polymers have been extensively studied. There are currently very few reports that describe the reorganization (e.g. erasing, changing, revealing, or rewriting) of information sequences^6,12,13^. For biological polymers such as nucleic acids, sequence rearrangement is not easy to achieve using a simple physical trigger and may require epigenetics modifications or the use of a bacterial machinery^12,13^. For synthetic polymers on the other hand, structural design can be adjusted to enable control over information sequences; e.g. using dynamic chemical bonds or stimuli-responsive functional groups^14–16^. For example, our group has reported that controlled thermal degradation can be used to erase information-containing polymers^6^. In this earlier study, main-chain thermolabile bonds were employed, thus leading to complete polymer destruction upon mild heating. Yet, temperature is not a practical trigger for all applications, in particular for devices that operate at room temperature. A possible alternative is the use of light as a stimulus. Photo-irradiation has been widely studied in academia and industry to control the synthesis^17,18^, folding^19,20^, self-assembly^21,22^, and physico-chemical properties^23–25^ of synthetic polymers. Here, we describe that light is a useful and versatile stimulus to control information sequences in digital polymers. The synthesis and characterization of digitally encoded polymers containing photo-controllable side-chains is presented herein. These polymers are constructed using four custom-made monomers containing either light-sensitive or light-inert substituents. This small monomer library enables various possibilities of sequence transformation such as erasing, mutation, and revealing.

## Results

### Molecular design of the light-sensitive digital polymers

**Fig. 1:**
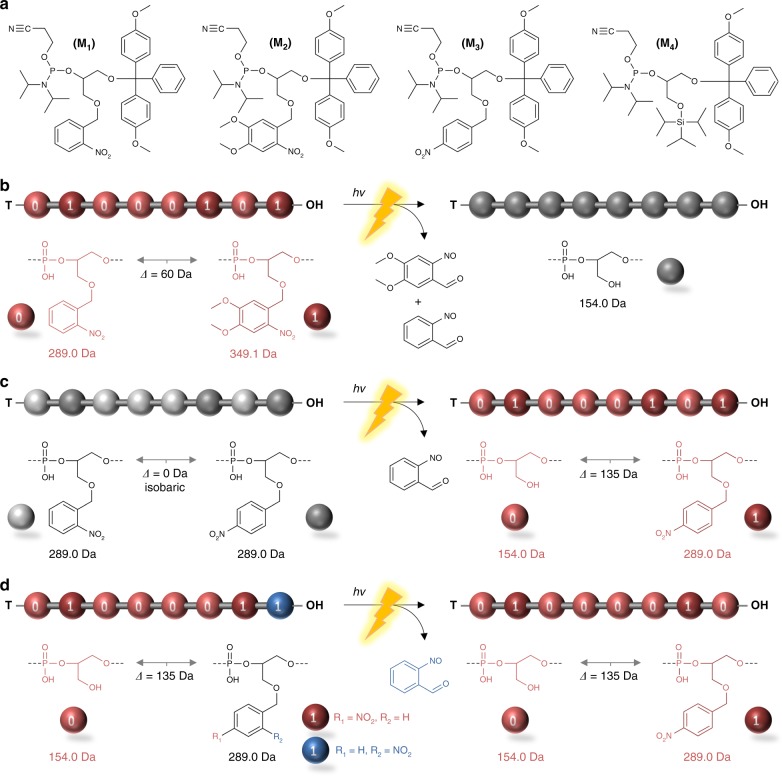
Monomer and polymer design. **a** Molecular structures of the different phosphoramidite monomers that were synthesized and used in the present work. **b** Strategy for the preparation of photo-erasable digital polymers. In this case, monomers **M1** and **M2** are used as 0- and 1-bits, respectively. These monomers have different molar mass and therefore allow construction of digital sequences that can be decoded by MS/MS. Upon light irradiation, both 0 and 1 units are photo-cleaved, thus leading to a non-decodable homopolymer (depicted in gray). **c** Strategy for the preparation of polymers containing hidden messages. In this case, isobaric monomers **M1** and **M3** are used as an invisible binary ink, which lead to MS/MS non-decodable sequences. Light is then used as a revealer. Photo-irradiation cleaves selectively *ortho*-nitrobenzyl units, thus leading to a MS/MS decodable binary sequence. **d** Strategy for photo-induced site-directed mutations. In this case, the polymers are constructed using monomers **M1**, **M3**, and **M4**. After TIPS deprotection, the formed digital polymer contains isobaric 1-bits (depicted in red and blue). Light irradiation allows selective 1→0 mutation of a single type of 1 unit (i.e. only the blue one). The letter T symbolizes a terminal thymidine nucleoside unit in panels **b**, **c**, and **d**

Figure 1 depicts the different concepts that were studied in the present work. Sequence-coded poly(phosphodiester)s were selected as model polymers^26^, even though the concepts described in this article could be potentially applied to various other types of information-containing macromolecules^3^. Abiotic poly(phosphodiester)s are prepared by automated solid-phase chemistry using phosphoramidite protocols that have been originally introduced for chemical nucleic acid synthesis^27,28^. It has been already described in previous works that long digital poly(phosphodiester)s can be synthesized by this approach^29,30^ and decoded by mass spectrometry^31^. In order to control information sequences by light, four phosphoramidite monomers **M**_**1**_–**M**_**4**_ were synthesized and characterized in this work (Fig. 1a). Monomers **M**_**1**_ and **M**_**2**_ contain light-cleavable *o*-nitrobenzyl and *o*-nitroveratryl ether motifs, respectively^32^. Monomer **M3** contains a light inert *p*-nitrobenzyl ether substituent. Monomer **M4** contains a triisopropylsilyl (TIPS)-protected OH group. All monomers were derived in five steps from glycerol and characterized by high-resolution mass spectrometry (HRMS) as well as ^1^H, ^13^C, and ^31^P NMR (nuclear magnetic resonance) spectroscopy (Supplementary Methods). To illustrate the versatility of this monomer library, different sequence transformations were investigated in this work (Fig. 1b–d). In the first strategy (Fig. 1b), photo-irradiation is used to erase information stored inside a synthetic polymer. To do so, the polymer is encoded using monomers **M**_**1**_ and **M**_**2**_ as a binary language. These two monomers have a different molar mass and it is therefore possible to write a digital sequence that can be decoded by tandem mass spectrometry (MS/MS)^2^. However, under light exposure, both *o*-nitrobenzyl and *o*-nitroveratryl side-chain moieties are cleaved, thus leading to a non-coded homopolymer. This concept permits to erase molecularly stored information without destroying the polymer main chain. In the second strategy (Fig. 1c), light is used as a trigger to reveal a hidden message stored in a polymer. Isobaric monomers **M**_**1**_ and **M**_**3**_ are used as bits 0 and 1 in this approach. Since these two monomers have the same molar mass, the formed sequence-coded polymer cannot be decrypted by MS/MS. However, under light exposure, the *o*-nitrobenzyl side-chains of bit-0 are cleaved, while the *p*-nitrobenzyl ether motifs remain unchanged. As a consequence, bit-0 and bit-1 have a different molar mass and their sequence can hence be revealed by MS/MS. The third approach (Fig. 1d) is inspired by irradiation-mediated mutagenesis and enables site-directed mutations in synthetic polymers. In this strategy, the polymers were synthesized using monomers **M**_**1**_, **M**_**3**_, and **M**_**4**_. After synthesis, the TIPS groups of **M**_**4**_ are cleaved, thus leading to a digital polymer, in which OH side groups are 0-bits and isobaric monomers **M**_**1**_ and **M**_**3**_ are 1-bits. When exposed to light, a 1→0 mutation occurs exclusively on *o*-nitrobenzyl 1-bits. As a consequence, the meaning of the information sequence can be manipulated by light.

### Photo-erasable digital polymers

**Fig. 2:**
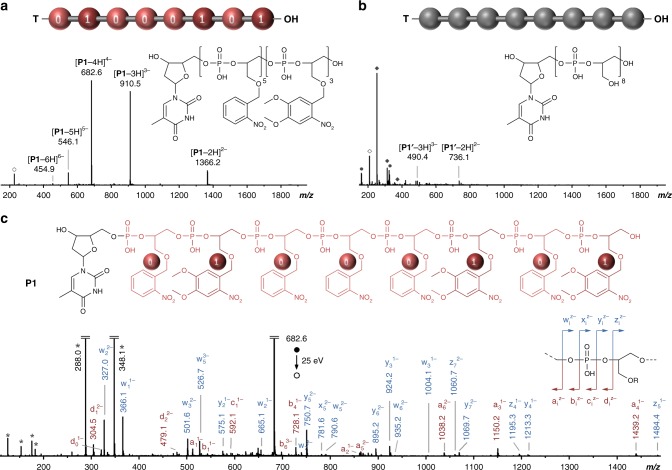
Example of a photo-erasable digital polymer. ESI-HRMS spectra obtained in the negative ion mode for polymer **P1** before (**a**) and after (**b**) light exposure. Open and full dark gray circles indicate clusters of trifluoroacetic acid and trichloroacetic acid, respectively. Open and full dark gray diamonds indicate photo-deprotection by-products. See Supplementary Figs. 6, 7 and 15 for interpretation. **c** MS/MS sequencing of the coded polymer **P1** before photo-erasing. This spectrum was obtained by collision-induced dissociation of the [**P1**-4H]^4-^ precursor ion. The inset schematizes the fragmentation pattern of a phosphate repeat unit. Dark gray stars indicate secondary fragments including deprotonated repeat units at *m/z* 288.0 and *m/z* 348.1

The photo-erasing concept was examined with a series of oligomers (Supplementary Table 1). Although long coded polymers can be prepared by phosphoramidite chemistry^29^, short models containing eight coded residues were studied herein for the sake of demonstration clarity. Since monomers **M1** and **M2** were never tested in solid-phase phosphoramidite chemistry, two homopolymers **H1** and **H2** were first synthesized and characterized by high-performance liquid chromatography (HPLC) (Supplementary Fig. 1), ^1^H NMR spectroscopy (Supplementary Figs. 2 and 3), electrospray ionization-HRMS (ESI-HRMS) (Supplementary Figs. 4–7), and ^31^P NMR spectroscopy (Supplementary Fig. 8). All these measurements evidenced formation of uniform oligomers, thus indicating that monomers **M1** and **M2** can be used in stepwise phosphoramidite protocols. These two homopolymers were also subjected to photo-irradiation (*λ* = 365 nm) in order to verify that *o*-nitrobenzyl and *o*-nitroveratryl units can be quantitatively cleaved from a poly(phosphodiester) backbone. The resulting photo-modified polymers **H1′** and **H2′** were analyzed by ESI-HRMS (Supplementary Figs. 4–7), which suggests full removal of the photo-cleavable side groups. Signals corresponding to partly deprotected oligomers could not be detected in these mass spectra. The formation of intermolecular thymine dimers, which generally occurs in the far-UV^33^, was also not detected. Digitally encoded copolymers **P1**–**P5**, in which monomers **M1** and **M2** were respectively used as 0-bit and 1-bit, were afterwards synthesized. Their molecular uniformity was confirmed by HPLC (Supplementary Fig. 1), ^31^P NMR (Supplementary Fig. 9), and ^1^H NMR (Supplementary Figs. 10–14). Figure 2 shows ESI-HRMS and MS/MS characterization of copolymer **P1** containing the digital sequence 01000101. Before irradiation, **P1** was found as a major species in MS (Fig. 2a) and could be sequenced by MS/MS to recover digital information (Fig. 2c, Supplementary Table 4), applying the same dissociation rules as previously reported for poly(phosphodiester)s^31^ since the presence of the side groups did not induce any significant modification of the backbone fragmentation pattern. Yet, after irradiation at *λ* = 365 nm, the formation of a fully deprotected homopolymer **P1′** was evidenced by ESI-HRMS (Fig. 2b, Supplementary Figs. 6, 7 and 15). As expected, no digital information could be found in this polymer (Supplementary Fig. 16). Comparable results were obtained with **P2**–**P5** (Supplementary Figs. 17–24). Interestingly, the MS relative abundance of the low molecular weight by-products formed upon photo-exposure (Supplementary Figs. 6, 7 and 15) was found to be related to 0/1 comonomer composition in the original copolymers **P1**–**P5** (Supplementary Fig. 25). It shall also be remarked that the irradiated poly(phosphodiester)s contain pendant OH-groups and, if need be, may be further degraded via a hydrolysis mechanism similar to the one of RNA^34^.

### Digital polymers containing photo-revealable sequences

**Fig. 3:**
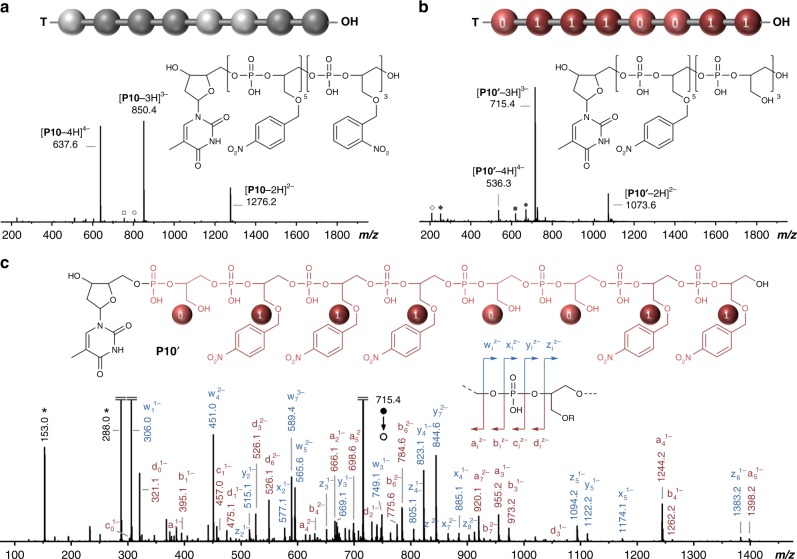
Characterization of a digital polymer coded with an invisible monomer ink. ESI-HRMS spectra obtained in the negative ion mode for polymer **P10** before (**a**) and after (**b**) light exposure. Squares and circles indicate synthesis impurities. Open and full dark gray diamonds indicate photo-deprotection by-products. See Supplementary Fig. 6 for interpretation. **c** MS/MS sequencing of the irradiated polymer **P10′**. This spectrum was obtained by collision-induced dissociation of the [**P10′**-3H]^3−^ precursor ion. The inset schematizes the fragmentation pattern of a phosphate repeat unit. Dark gray stars indicate secondary fragments including deprotonated repeat units at *m/z* 153.0 and *m/z* 288.0

Monomers **M1** and **M3** were then used to build oligomers containing light-revealable information sequences (Supplementary Table 2). In order to confirm that polymers containing *p*-nitrobenzyl ether units are not photo-sensitive, homopolymer **H3** was first synthesized using monomer **M3** and characterized. HPLC (Supplementary Fig. 26) and ^1^H NMR (Supplementary Fig. 27) measurements indicated synthesis of a uniform molecule, thus validating that **M3** leads to high-yields coupling steps in solid-phase iterative synthesis. Furthermore, as shown by MS (Supplementary Fig. 28), the molecular structure of this polymer remained unchanged before and after photo-irradiation, and MS/MS data confirmed the expected monotonic sequence of **H3** (Supplementary Fig. 29). Based on these results, a series of coded oligomers **P6**–**P11** containing different information sequences was prepared and characterized by HPLC (Supplementary Fig. 26), ^1^H NMR spectroscopy (Supplementary Figs. 30–35), ^31^P NMR spectroscopy (data not shown), and mass spectrometry. All these copolymers were photo-irradiated at *λ* = 365 nm, thus leading to modified structures **P6′**–**P11′**. Figure 3 compares the ESI-HRMS spectra obtained for **P10**, which contains a monomer sequence composed of five *p*-nitrobenzyl and three *o*-nitrobenzyl units, and the corresponding photo-irradiated oligomer **P10′**. The dominating species observed before irradiation has a molar mass of 2554 Da, whereas after light exposure an oligomer of 2149 Da is observed. The mass difference of 405 Da (i.e., 3 × 135 Da) observed between both species corresponds to the release of three nitrobenzyl moieties. This suggests that the *o*-nitrobenzyl units are selectively and quantitatively cleaved from the polymer backbone upon irradiation. Moreover, before irradiation, MS/MS sequencing of **P10** (Supplementary Fig. 36) evidenced a monotonic sequence, in which isobaric *o*-nitrobenzyl and *p*-nitrobenzyl units cannot be distinguished from another. The sequence can also not be decrypted by ^1^H NMR spectroscopy (Supplementary Fig. 34), which only gives rough information about *ortho*/*para* comonomer composition. Yet, after photo-exposure, the digital sequence 01110011 can be extracted from the MS/MS spectrum of **P10′**, as shown in Fig. 3c and Supplementary Table 5. These results confirm that monomers **M1** and **M3** can be efficiently used as an invisible binary ink to encrypt synthetic digital polymers. Comparable results were obtained with all other copolymers of the series, namely **P6**–**P9** and **P11** (Supplementary Figs. 37–46).

### Site-directed photo-mutations

**Fig. 4:**
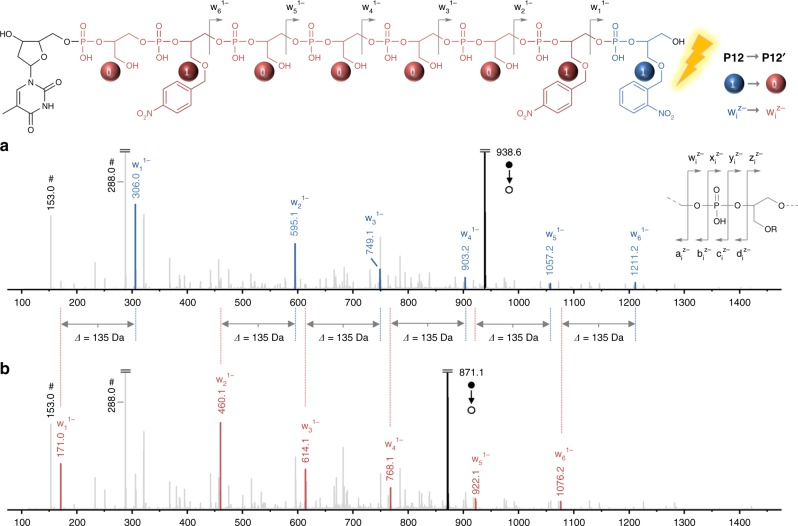
Photo-induced sequence mutation. MS/MS sequencing spectra obtained for polymer **P12** before (**a**) and after (**b**) light exposure. The top and bottom spectra were obtained by collision-induced dissociation of the [**P12**-2H]^2−^ and [**P12′**-2H]^2−^ precursor ions, respectively. In these conditions, a phosphate repeat unit leads to different fragments, as indicated by the gray fragmentation pattern on the right side of panel **a**. However, for clarity, only w_i_^z−^ fragments are highlighted in this figure. The corresponding peaks were intentionally thickened and colored (blue before- and red after irradiation) with the help of the Origin software. All other fragments are intentionally displayed in light gray. A detailed interpretation of all peaks can be found in Supplementary Table 6. Black hashtags indicate deprotonated repeat units at m/z 153.0 and m/z 288.0

The strategy allowing site-directed mutations in synthetic polymers was then examined. A photo-mutable poly(phosphodiester) was synthesized using monomers **M1**, **M3**, and **M4** (Supplementary Table 3). **M4** contains two OH functions that are orthogonally protected by 4,4'-dimethoxytrityl (DMT) and TIPS groups. The DMT-protected functions are selectively deprotected and reacted in stepwise phosphoramidite protocols, while the TIPS-protected pendant groups remain intact. After performing solid-phase synthesis, the TIPS groups are removed, thus resulting in a digital polymer in which OH-functional side groups represents 0-bits while isobaric monomers **M1** and **M3** both code for 1-bits. Using this language, the ASCII sequence 01000011, coding for the letter C, was stored in copolymer **P12**. The formed polymer was characterized by HPLC (Supplementary Fig. 47), ^1^H NMR spectroscopy (Supplementary Fig. 48), and ESI-MS (Supplementary Fig. 49). These measurements evidenced formation of a uniform molecule, in which all TIPS group have been removed. In copolymer **P12**, only one **M1** unit is photo-labile and consequently a target site for directed photo-mutation. Upon photo-exposure, **M1** loses its *o*-nitrobenzyl moiety, thus resulting in the formation of a 0-bit (*i.e*. 1→0 mutation). As a consequence, the photo-modified polymer **P12′** contains the ASCII sequence 01000010 coding for the letter B. Supplementary Fig. 49 and Fig. 4 compare the ESI-MS and MS/MS sequencing data that were acquired for **P12** before and after photo-irradiation. For clarity, only one series of fragments (noted w_i_^z−^) is highlighted in Fig. 4, whereas the full interpretation of the spectra can be found in Supplementary Table 6. These results clearly evidence that the 1→0 mutation occurred and led to a modified information sequence. When a single **M1** site is mutated, the overall molar mass of the oligomer shall decrease from 1879 to 1744 Da (i.e. −135 Da), which is what was observed in ESI-MS. Moreover, the fact that the molar mass of the MS/MS fragment w_1_^1−^ decreases of 135 Da after irradiation (Fig. 4) indicates unequivocally site-directed mutation. Since the mutation site is located at the extremity of the sequence in the studied example, the mass decrease of 135 Da is also echoed in all ions of the w_i_^z−^ fragment series, as highlighted in Fig. 4. This concept is also applicable to longer information sequences. The atomic symbol of copper that is Cu was ASCII-encoded in copolymer **P13** using **M1**, **M3**, and **M4** (Supplementary Table 3). In this sequence, a single 1→0 photo-mutation site was implemented at the seventh monomer position, starting from the thymidine end-group. Thus, upon photo-irradiation, the information sequence is transformed into the ASCII-encoded atomic symbol of gold Au (Supplementary Figs. 50, 51 and 52 and Supplementary Tables 3, 7 and 8).

## Discussion

The experimental results described in this article evidence that the information sequences of synthetic polymers can be easily manipulated by light. Various types of sequence modification (i.e. erasing, revealing, mutation) can be attained using a rather simple set of monomers. As already specified above, the concepts described herein are not restricted to digital poly(phosphodiester)s and can possibly be transposed to many other types of information-containing macromolecules^3^. Overall, these results open up interesting avenues for the design of functional polymer materials. For instance, photo-erasable polymers are interesting structures for data storage applications; e.g., for the development of polymer-based digital micro-arrays^3^. Indeed, such polymers could be exploited to erase specific information zones in a 2D micropattern^35^. Polymers containing photo-revealable information sequences could be of great relevance for anti-counterfeiting applications^36^. Indeed, beyond standard monomer sequence encryption^1^, the fact that monomer sequences can be hidden and revealed gives an additional level of security and secrecy to macromolecular taggants. Perhaps even more importantly, it was shown in this work that specific sequence mutations can be induced in synthetic macromolecules. Such an effect can be used to tune the meaning of polymer-stored information sequences, as evidenced herein. Yet, beyond primary structure modification, site-directed mutations could be used to induce conformational changes, to trigger latent functions, or to generate an evolutionary behavior in synthetic macromolecules.

## Methods

### Materials and reagents

Acetic acid (99.8%, Fischer Chemical), acetone (Carlo Erba), anhydrous acetonitrile (99.8%, anh. MeCN, Sigma-Aldrich), aqueous ammonia (28 w% NH_3_ in H_2_O, VWR), ammonium chloride (99.5%, VWR), carbon tetrabromide (99%, CBr_4_, TCI), 2-cyanoethyl-*N*,*N*-diisopropylchlorophosphoramidite (97%, ABCR), cyclohexane (Carlo Erba), anhydrous dichloromethane (99.8%, anh. DCM, cont. amylene as a stabilizer, Sigma-Aldrich), *N*,*N*-diisopropylethylamine (>99.0%, DIPEA, TCI), 4,4′-dimethoxytrityl chloride (DMTr-Cl, ChemGenes), ethyl acetate (EtOAc, Carlo Erba), glycerol (1,2,3-propanetriol, cont. water, Merck), anhydrous methanol (99.8%, anh. MeOH, Sigma-Aldrich), aqueous methylamine (40 w% MeNH_2_ in H_2_O, Fluka), methyllithium (1.6 M in Et_2_O, Sigma-Aldrich), molecular sieves (3 Å, Merck), 2-nitrobenzyl bromide (98%, Alfa Aesar), 4-nitrobenzyl bromide (97%, Alfa Aesar), 4,5-dimethoxy-2-nitrobenzaldehyde (98%, Alfa Aesar), *para*-toluenesulfonic acid monohydrate (99%, Sigma-Aldrich), anhydrous pyridine (99.8%, anh. pyr, Sigma-Aldrich), silica gel (high-purity grade, pore size 60 Å, 230–400 mesh, for flash chromatography, SiO_2_, Fluka), sodium borohydride (98%, Aldrich), sodium chloride (for brine solution, ESCO), sodium hydride (60 w% suspension in mineral oil, Aldrich), anhydrous sodium sulfate (99.6%, VWR), anhydrous toluene (99.8%, anh. toluene, Sigma-Aldrich), triisopropylsilyl chloride (97%, TIPSCl, Sigma-Aldrich), triethylamine trihydrofluoride (98%, Et_3_N.3HF, Sigma-Aldrich), triethylamine (97%, Et_3_N, Acros Organics), and triphenylphosphine (PPh_3_, 95%, TCI) were used as received without further purification. (2,2-Dimethyl-1,3-dioxolan-4-yl)methanol and 4,5-dimethoxy-2-nitrobenzyl alcohol were synthesized following published procedures^37,38^. Reagents for automated phosphoramidite synthesis: anhydrous acetonitrile (phosphoramidite diluent & dry washings, ChemGenes), acetonitrile (≥99.9%, washings, Roth), activation reagent (0.25 M 5-ethylthio tetrazole in MeCN, ChemGenes), Cap A (acetic anhydride/pyridine/THF, ChemGenes), Cap B (10% *N*-methylimidazole in THF, ChemGenes), DMT removal reagent (3 w% TCA in DCM, Roth), drying traps (small, 10–15 mL, ChemGenes), dT-CPG 1000 (1 μmol in cartridge, 1000 Å pore size, Glen Research), oxidizer (0.02 M iodine/pyridine/H_2_O/THF, ChemGenes) were used as received.

### Synthesis of phosphoramidite monomer M1

Monomer **M1** was synthesized in four steps as shown in Supplementary Fig. 53. Synthesis details are described as follows. **2,2-dimethyl-4-(((2-nitrobenzyl)oxy)methyl)-1,3-dioxolane 1**. To a stirred solution of (2,2-dimethyl-1,3-dioxolan-4-yl)methanol (801 mg, 6.1 mmol, 1.2 eq.) and 2-nitrobenzyl bromide (1088 mg, 5.0 mmol, 1.0 eq.) in 40 mL MeCN (anh.) at 0 °C was added NaH (479 mg, 60 w%, 12.0 mmol, 2.4 eq.) at 0 °C under argon. The mixture was stirred for 30 min at 0 °C. Then, it was allowed to reach RT and the solvent was removed. Fifty milliliters NH_4_Cl (aq., sat.) was added and it was extracted with EtOAc (3 × 50 mL). The combined organic layer was washed with brine (2 × 50 mL), dried over Na_2_SO_4_, filtered, and the solvent was removed. The yellow crude oil was purified by column chromatography (SiO_2_ vs 10 v% → 20 v% EtOAc in cyclohexane) to obtain 658 mg (2.5 mmol, 49%) of **1** as a pale yellow oil. HRMS *m/z*: [M + H]^+^ calculated for C_13_H_18_NO_5_^+^ 268.1179; found 268.1181; ^1^H NMR (400.1 MHz, CDCl_3_, *δ*, ppm): 1.38 (s, 3H), 1.44 (s, 3H,), 3.60–3.69 (m, 2H), 3.79 (m, 1H), 4.10 (m, 1H), 4.36 (m, 1H), 4.96 (s, 2H), 7.42–7.47 (m, 1H, ArH), 7.62–7.67 (m, 1H, ArH), 7.80 (m, 1H), 8.06 (m, 1H); ^13^C NMR (100.6 MHz, CDCl_3_, δ, ppm): 25.5, 26.8, 66.7, 70.1, 72.2, 74.7, 109.6, 124.7, 128.1, 128.8, 133.7, 134.8, 147.3. **3-((2-nitrobenzyl)oxy)propane-1,2-diol 2**. Dioxolan **1** (860 mg, 3.2 mmol) was dissolved in 43 mL AcOH (aq., 10 w%) and the solution was stirred at 60 °C for 5 h. The mixture was extracted with EtOAc (3 × 50 mL). The combined organic layer was dried over Na_2_SO_4_, filtered, and the solvent was removed. The crude was purified by column chromatography (SiO_2_ vs 50 v% → 100 v% EtOAc in hexane) to obtain 680 mg (3.0 mmol, 93%) of the product as a yellow oil. HRMS *m/z*: [M + Na]^+^ calculated for C_10_H_13_NO_5_Na^+^ 250.0686; found 250.0679; ^1^H NMR (400.1 MHz, CDCl_3_, *δ*, ppm): 2.21 (bs, 2H), 3.63–3.78 (m, 4H), 3.94–3.99 (m, 1H), 4.92 (s, 2H), 7.43–7.49 (m, 1H), 7.62–7.67 (m, 1H), 7.71 (m, 1H), 8.04 (m, 1H); ^13^C NMR (100.6 MHz, CDCl_3_, δ, ppm): 63.9, 70.1, 70.9, 72.5, 124.8, 128.3, 128.9, 133.7, 134.3, 147.5. **1-(bis(4-methoxyphenyl)(phenyl)methoxy)-3-((2-nitrobenzyl)oxy)propan-2-ol 3**. To a stirred solution of diol **2** (241 mg, 1.1 mmol, 1.0 eq.) in 5 mL THF (anh.):pyridine (anh.) = 3: 2 (v:v) 4,4′-dimethoxytrityl chloride (360 mg, 1.1 mmol, 1.0 eq.) was added in three intervals of each 40 min at RT under argon atmosphere and stirred overnight. The solvent was removed, 20 mL NaHCO_3_ (sat., aq.) was added, and it was extracted with EtOAc (3 × 50 mL). The combined organic layer was washed with brine (2 × 25 mL), dried over Na_2_SO_4_, filtered, and the solvent was removed. The yellow crude oil was purified by column chromatography (SiO_2_ vs 20 v% → 33 v% EtOAc in cyclohexane + 3 v% DIPEA) to obtain 493 mg (0.9 mmol, 88%) of the product as a yellowish foam. HRMS *m/z*: [M + Na]^+^ calculated for C_31_H_31_NO_7_Na^+^ 552.1993; found, 552.1996; ^1^H NMR (400.1 MHz, CDCl_3_, *δ*, ppm): 2.39 (m, 1H), 3.27 (m, 2H), 3.66–3.71 (m, 2H), 3.78 (s, 6H), 4.02 (m, 1H), 4.91 (s, 2H), 6.82 (m, 4H), 7.18–7.23 (m, 1H), 7.27–7.34 (m, 6H), 7.40–7.46 (m, 3H), 7.57–7.63 (m, 1H), 7.69 (m, 1H), 8.07 (m, 1H). ^13^C NMR (100.6 MHz, CDCl_3_, δ, ppm): 55.3, 64.4, 70.0, 70.1, 72.6, 86.3, 113.3, 124.8, 126.9, 128.0, 128.1, 128.2, 128.7, 130.2, 133.8, 135.0, 136.0, 144.9, 147.2, 158.6. **1-(bis(4-methoxyphenyl)(phenyl)methoxy)-3-((2-nitrobenzyl)oxy)propan-2-yl (2-cyanoethyl) diisopropylphosphoramidite M1**. 1136 mg of **3** (2.1 mmol, 1.0 eq.) were dissolved in 10 mL anhydrous DCM. Then, DIPEA (1660 mg, 13.7 mmol, 6.0 eq.), molecular sieves (3 Å) and 2-cyanoethyl-*N*,*N*-diisopropylchlorophosphoramidite (581 mg, 2.5 mmol, 1.15 eq.) were added under argon atmosphere at 0 °C. The reaction mixture was stirred at 0 °C for 30 min and then allowed to reach RT. DCM was removed and the residue was purified by column chromatography (SiO_2_ vs 33 v% EtOAc in cyclohexane + 3 v% DIPEA) to obtain 1508 mg (2.1 mmol, 97%) of **M1** as a yellowish foam. HRMS *m/z*: [M + H]^+^ calculated for C_40_H_49_N_3_O_8_P^+^ 730.3252; found 730.3250; ^1^H NMR (400.1 MHz, CDCl_3_, *δ*, ppm): 1.04–1.20 (m, 12H), 2.42–2.60 (m, 2H), 3.18–3.37 (m, 2H), 3.50–3.65 (m, 2H), 3.69–3.89 (m, 4H), 3.78 (s, 6H), 4.20 (m, 1H), 4.87–4.95 (m, 2H), 6.77–6.84 (m, 4H), 7.17–7.22 (m, 1H), 7.24–7.28 (m, 2H), 7.30–7.36 (m, 4H), 7.40–7.48 (m, 3H), 7.54–7.62 (m, 1H), 7.70–7.76 (m, 1H), 8.05–8.10 (m, 1H); ^13^C NMR (100.6 MHz, CDCl_3_, δ, ppm): 20.4, 24.5, 24.7, 43.3, 55.3, 58.5, 63.9, 69.9, 72.1, 72.6, 86.1, 113.1, 117.7, 124.7, 126.8, 127.9, 128.3, 128.4, 128.7, 130.2, 133.7, 135.5, 136.2, 145.0, 147.0, 158.5; ^31^P NMR (161.9 MHz, CDCl_3_, δ, ppm): 149.4.

### Synthesis of phosphoramidite monomer M2

Monomer **M2** was synthesized in four steps as shown in Supplementary Fig. 54. In the first step, (2,2-dimethyl-1,3-dioxolan-4-yl)methanol was reacted with 4,5-dimethoxy-2-nitrobenzyl bromide. The latter compound was synthesized in one step as shown in Supplementary Fig. 53b. **4,5-dimethoxy-2-nitrobenzyl bromide 4**. PPh_3_ (2620 mg, 10.0 mmol, 1.5 eq.) was added at 0 °C and under argon atmosphere to a stirred solution of 4,5-dimethoxy-2-nitrobenzyl alcohol (1424 mg, 6.7 mmol, 1.0 eq.) and CBr_4_ (3324 mg, 10 mmol, 1.5 eq.) in 40 mL anhydrous THF. The reaction mixture was stirred overnight and then allowed to reach RT. After reaction, the mixture was filtered and evaporated. The crude was purified by column chromatography (SiO_2_ vs 10 v% → 40 v% EtOAc in hexane) to obtain 1690 mg (6.1 mmol, 92%) of the product as a pale yellow crystalline solid. ^1^H NMR (400.1 MHz, CDCl_3_, *δ*, ppm): 3.96 (s, 3H, ArOCH_3_), 4.00 (s, 3H, ArOCH_3_), 4.87 (s, 2H, PhCH_2_Br), 6.94 (s, 1H, ArH), 7.67 (s, 1H, ArH); ^13^C NMR (100.6 MHz, CDCl_3_, δ, ppm): 30.2, 56.6, 56.7, 108.7, 113.8, 127.6, 140.4, 149.1, 153.3. **4-(((4,5-dimethoxy-2-nitrobenzyl)oxy)methyl)-2,2-dimethyl-1,3-dioxolane 5**. To a stirred solution of (2,2-dimethyl-1,3-dioxolan-4-yl)methanol (620 mg, 4.7 mmol, 1.0 eq.) in 12 mL MeCN (anh.) at 0 °C, NaH (226 mg, 60 w%, 5.7 mmol, 1.2 eq.) was added under argon atmosphere. The suspension was stirred 10 min at 0 °C and a solution of **4** (1690 mg, 6.1 mmol, 1.3 eq.) dissolved in 5 mL DCM (anh.) and 5 mL MeCN (anh.) was added. The reaction mixture was stirred for 15 min at 0 °C and for further 3 h allowed to reach RT. The solvent was removed, 20 mL H_2_O was added and it was extracted with DCM (3 × 50 mL). The combined organic layer was washed with brine (1 × 50 mL), dried over Na_2_SO_4_, filtered and the solvent was removed. The orange crude solid was purified by column chromatography (SiO_2_ vs 20 v% → 30 v% EtOAc in hexane) to obtain 840 mg (2.6 mmol, 54%) of the product as a yellow solid. 1.5 mmol of bromide **4** were also recovered. HRMS *m/z*: [M + NH_4_]^+^ calculated for C_15_H_25_N_2_O_7_^+^ 345.1656; found 345.1657; ^1^H NMR (400.1 MHz, CDCl_3_, *δ*, ppm): 1.39 (s, 3H), 1. 44 (s, 3H), 3.68 (m, 2H), 3.82 (m, 1H), 3.95 (s, 3H), 3.97 (s, 3H), 4.10 (m, 1H), 4.39 (m, 1H), 4.98 (s, 2H), 7.33 (s, 1H), 7.71 (s, 1H, ArH); ^13^C NMR (100.6 MHz, CDCl_3_, δ, ppm): 25.5, 26.8, 56.4, 56.5, 66.5, 70.2, 72.0, 74.9, 108.0, 109.5, 109.7, 130.8, 139.3, 147.7, 153.9. **3-((4,5-dimethoxy-2-nitrobenzyl)oxy)propane-1,2-diol 6**. 840 mg of **5** (2.6 mmol) was dissolved in 5 mL THF and 34 mL AcOH (aq., 10 w%). The solution was stirred at 60 °C for 2.5 h. The mixture was extracted with EtOAc (3 × 50 mL). The combined organic layer was dried over Na_2_SO_4_, filtered and the solvent was removed. The crude was purified by column chromatography (SiO_2_ vs EtOAc) to obtain 720 mg (2.5 mmol, 98%) of the product as a yellow solid. HRMS *m/z*: [M + NH_4_]^+^ calculated for C_12_H_21_N_2_O_7_^+^ 305.1343; found, 305.1349; ^1^H NMR (400.1 MHz, CD_3_OD, *δ*, ppm): 3.56–3.69 (m, 4H), 3.83–3.88 (m, 1H), 3.90 (s, 3H), 3.97 (s, 3H), 4.91 (s, 2H), 7.43 (s, 1H), 7.72 (s, 1H); ^13^C NMR (100.6 MHz, CD_3_OD, δ, ppm): 56.8, 56.9, 64.4, 71.0, 72.3, 73.5, 109.1, 111.2, 131.7, 140.7, 149.2, 155.3. **1-(bis(4-methoxyphenyl)(phenyl)methoxy)-3-((4,5-dimethoxy-2-nitrobenzyl)oxy)propan-2-ol 7**. To a stirred solution of diol **6** (670 mg, 2.3 mmol, 1.0 eq.) in 10 mL THF (anh.):pyridine (anh.) = 3:2 (v:v) 4,4′-dimethoxytrityl chloride (790 mg, 2.3 mmol, 1.0 eq.) was added in three intervals of each 40 min at RT, under argon atmosphere and stirred overnight. The solvent was removed, 20 mL NaHCO_3_ (sat., aq.) were added and it was extracted with EtOAc (3 × 50 mL). The combined organic layer was washed with brine (2 × 50 mL), dried over Na_2_SO_4_, filtered and the solvent was removed. The yellow crude oil was purified by column chromatography (SiO_2_ vs 30 v% → 50 v% EtOAc in cyclohexane + 3 v% DIPEA) to obtain 1050 mg (1.8 mmol, 76%) of the product as a yellowish foam. HRMS *m/z*: [M + Na]^+^ calculated for C_33_H_35_NO_9_Na^+^ 612.2204; found, 612.2203; ^1^H NMR (400.1 MHz, CDCl_3_, *δ*, ppm): 2.43 (m, 1H), 3.27 (m, 2H), 3.65–3.73 (m, 2H), 3.77 (s, 6H), 3.88 (s, 3H), 3.95 (s, 3H), 4.07 (m, 1H), 4.92 (m, 2H), 6.81 (m, 4H), 7.17–7.24 (m, 2H), 7.27–7.33 (m, 6H), 7.42 (m, 2H), 7.71 (s, 1H); ^13^C NMR (100.6 MHz, CDCl_3_, δ, ppm): 55.3, 56.4, 64.6, 70.1, 70.2, 72.6, 86.3, 108.0, 109.5, 113.2, 127.0, 128.0, 128.2, 130.1, 130.9, 135.9, 139.1, 144.8, 147.6, 153.9, 158.6. **1-(bis(4-methoxyphenyl)(phenyl)methoxy)-3-((4,5-dimethoxy-2-nitrobenzyl)oxy)propan-2-yl (2-cyanoethyl) diisopropylphosphoramidite M2**. DIPEA (584 mg, 4.5 mmol, 6.0 eq.) and molecular sieves (3 Å) were added at 0 °C under argon atmosphere to a solution of **7** (420 mg, 0.7 mmol, 1.0 eq.) in 5 mL anhydrous DCM. Then, 2-cyanoethyl-*N*,*N*-diisopropylchlorophosphoramidite (189 mg, 0.8 mmol, 1.12 eq.) was added. The reaction mixture was stirred at 0 °C for 30 min and then allowed to reach RT. The solvent was removed and the residue purified by column chromatography (SiO_2_ vs 30 v% EtOAc in cyclohexane + 3 v% DIPEA) to obtain 527 mg (0.7 mmol, 94%) of the product as a yellowish foam. HRMS *m/z*: [M + H]^+^ calculated for C_42_H_53_N_3_O_10_P^+^ 790.3463; found 790.3471; ^1^H NMR (400.1 MHz, CDCl_3_, *δ*, ppm): 1.04–1.20 (m, 12H), 2.42–2.60 (m, 2H), 3.17–3.34 (m, 2H), 3.50–3.67 (m, 2H), 3.70–3.92 (m, 4H), 3.77 (m, 6H), 3.85 (m, 3H), 3.95 (m, 3H), 4.24 (m, 1H), 4.88–4.98 (m, 2H), 6.75–6.83 (m, 4H), 7.16–7.24 (m, 2H), 7.27–7.35 (m, 6H), 7.41–7.47 (m, 2H), 7.71 (m, 1H); ^13^C NMR (100.6 MHz, CDCl_3_, δ, ppm): 20.3, 24.6, 43.3, 55.3, 56.4, 58.4, 64.1, 70.2, 72.5, 86.1, 107.8, 109.5, 113.1, 117.7, 126.8, 127.8, 128.3, 130.2, 131.3, 136.1, 138.8, 145.0, 147.5, 153.9, 158.5; ^31^P NMR (161.9 MHz, CDCl_3_, δ, ppm): 149.4.

### Synthesis of phosphoramidite monomer M3

Monomer **M3** was synthesized in four steps as shown in Supplementary Fig. 55. Synthesis details are described as follows. **2,2-dimethyl-4-(((4-nitrobenzyl)oxy)methyl)-1,3-dioxolane 8**. 441 mg of NaH (60 w%, 11.0 mmol, 1.1 eq.) was added at 0 °C and under argon atmosphere to a stirred solution of (2,2-dimethyl-1,3-dioxolan-4-yl)methanol (1328 mg, 10.0 mmol, 1.0 eq.) and 4-nitrobenzyl bromide (3247 mg, 15.0 mmol, 1.5 eq.) in 40 mL of anhydrous MeCN. The reaction mixture was stirred for 30 min at 0 °C and then allowed to reach RT for 30 min. The reaction mixture was filtered. The solvent was removed from the filtrate and the crude directly purified by column chromatography (SiO_2_ vs 10 v% → 20 v% EtOAc in cyclohexane) to obtain 1077 mg (4.0 mmol, 40%) of the product as a yellow oil. HRMS *m/z*: [M + H]^+^ calculated for C_13_H_18_NO_5_^+^ 268.1179; found, 268.1180; ^1^H NMR (400.1 MHz, CDCl_3_, *δ*, ppm): 1.38 (s, 3H), 1.44 (s, 3H), 3.53–3.64 (m, 2H), 3.77 (m, 1H), 4.09 (m, 1H), 4.34 (m, 1H), 4.68 (s, 2H), 7.51 (m, 2H), 8.21 (m, 2H); ^13^C NMR (100.6 MHz, CDCl_3_, δ, ppm): 25.5, 26.9, 66.7, 71.9, 72.4, 74.8, 109.7, 123.8, 127.8, 145.8, 147.5. **3-((4-nitrobenzyl)oxy)propane-1,2-diol 9**. 1030 mg of **8** (3.8 mmol) was dissolved in 50 mL AcOH (aq., 10 w%). The solution was stirred at 60 °C for 2.5 h. The mixture was extracted with EtOAc (3 × 55 mL). The combined organic layer was dried over Na_2_SO_4_, filtered and the solvent was removed. The crude was purified by column chromatography (SiO_2_ vs EtOAc) to obtain 840 mg (3.7 mmol, 96%) of the product as a yellow oil. HRMS *m/z*: [M + Na]^+^ calculated for C_10_H_13_NO_5_Na^+^ 250.0686; found, 250.0687; ^1^H NMR (400.1 MHz, CDCl_3_, *δ*, ppm): 2.00 (bs, 1H), 2.53 (bs, 1H), 3.57–3.80 (m, 4H), 3.92–4.00 (m, 1H), 4.67 (s, 2H), 7.50 (m, 2H), 8.22 (m, 2H); ^13^C NMR (100.6 MHz, CDCl_3_, δ, ppm): 64.0, 70.9, 72.4, 123.8, 127.9, 145.4, 147.6. **1-(bis(4-methoxyphenyl)(phenyl)methoxy)-3-((4-nitrobenzyl)oxy)propan-2-ol 10**. To a stirred solution of diol **9** (790 mg, 3.5 mmol, 1.0 eq.) in 20 mL THF (anh.):pyridine (anh.) = 3:2 (v:v) 4,4′-dimethoxytrityl chloride (1180 mg, 3.5 mmol, 1.0 eq.) was added in three intervals of each 40 min at RT, under argon atmosphere and stirred overnight. The solvent was removed, 40 mL NaHCO_3_ (sat., aq.) was added and it was extracted with EtOAc (3 × 50 mL). The combined organic layer was washed with brine (2 × 30 mL), dried over Na_2_SO_4_, filtered, and the solvent was removed. The yellow crude oil was purified by column chromatography (SiO_2_ vs 20 v% → 40 v% EtOAc in cyclohexane + 3 v% DIPEA) to obtain 1590 mg (3.0 mmol, 86%) of the product as yellowish foam. HRMS *m/z*: [M + Na]^+^ calculated for C_31_H_31_NO_7_Na^+^ 552.1993; found, 552.1992; ^1^H NMR (400.1 MHz, CDCl_3_, *δ*, ppm): 2.37 (m, 1H), 3.25 (m, 2H), 3.58–3.66 (m, 2H), 3.79 (s, 6H), 4.00 (m, 1H), 4.62 (s, 2H), 6.81 (m, 4H), 7.16–7.24 (m, 1H), 7.27–7.33 (m, 6H), 7.39–7.44 (m, 4H), 8.17 (m, 1H); ^13^C NMR (100.6 MHz, CDCl_3_, δ, ppm): 55.3, 64.4, 70.1, 72.1, 72.2, 86.3, 113.3, 123.7, 127.0, 127.8 128.0, 128.2, 130.2, 136.0, 144.8, 145.8, 147.5, 158.7. **1-(bis(4-methoxyphenyl)(phenyl)methoxy)-3-((4-nitrobenzyl)oxy)propan-2-yl (2-cyanoethyl) diisopropylphosphoramidite M3**. DIPEA (1850 mg, 14.3 mmol, 6.6 eq.) and 100 mg of molecular sieves (3 Å) were added under argon atmosphere at 0 °C to a solution of DMT precursor **10** (1148 mg, 2.2 mmol, 1.0 eq.) in 10 mL of anhydrous DCM. Then, 2-cyanoethyl-*N*,*N*-diisopropylchlorophosphoramidite (600 mg, 2.5 mmol, 1.15 eq.) was added. The reaction mixture was stirred at 0 °C for 30 min and then allowed to reach RT. The solvent was removed and the residue was purified by column chromatography (SiO_2_ vs 33 v% EtOAc in cyclohexane + 3 v% DIPEA) to obtain 1521 mg (2.1 mmol, 96%) of the product as a yellowish foam. HRMS *m/z*: [M + H]^+^ calculated for C_40_H_49_N_3_O_8_P^+^ 730.3252; found, 730.3253; ^1^H NMR (400.1 MHz, CDCl_3_, *δ*, ppm): 1.04–1.20 (m, 12H), 2.42–2.61 (m, 2H), 3.17–3.35 (m, 2H), 3.50–3.65 (m, 2H), 3.66–3.89 (m, 4H), 3.78 (s, 6H), 4.18 (m, 1H), 4.57–4.65 (m, 2H), 6.76–6.84 (m, 4H), 7.17–7.24 (m, 1H), 7.24–7.27 (m, 2H), 7.28–7.35 (m, 4H), 7.36–7.46 (m, 4H), 8.12–8.18 (m, 2H); ^13^C NMR (100.6 MHz, CDCl_3_, δ, ppm): 20.4, 24.8, 43.3, 55.3, 58.4, 63.9, 71.8, 72.1, 72.6, 86.1, 113.2, 117.8, 123.6, 126.9, 127.6, 127.9, 128.3, 130.2, 136.2, 145.0, 146.2, 147.4, 158.6; ^31^P NMR (161.9 MHz, CDCl_3_, δ, ppm): 149.4.

### Synthesis of phosphoramidite monomer M4

Monomer **M4** was synthesized in four steps as shown in Supplementary Fig. 56. Synthesis details are described as follows. Intermediates **11** and **12** were synthesized following reported procedures^39^. **1-(bis(4-methoxyphenyl)(phenyl)methoxy)-3-((triisopropylsilyl)oxy)propan-2-ol 13**. To a stirred solution of TIPS-protected glycerol (3641 mg, 14.7 mmol, 1.0 eq.) in 30 mL of 3:2 (v:v) mixture of THF (anh.):pyridine (anh.), was added at RT under argon atmosphere, 4,4′-dimethoxytrityl chloride (4968 mg, 14.7 mmol, 1.0 eq.) in three intervals of each 40 min. The mixture was then stirred overnight. The solvent was removed and 50 mL NaHCO_3_ (sat., aq.) was added. The mixture was extracted with EtOAc (3 × 100 mL), the combined organic layer was washed with brine (2 × 50 mL), dried over Na_2_SO_4_, filtered, and the solvent was removed. The obtained yellow crude oil was purified by column chromatography (SiO_2_ vs 40 v% EtOAc in cyclohexane + 2 v% DIPEA) to obtain 6279 mg (11.4 mmol, 78%) of **13** as a colorless oil. HRMS *m/z*: [M + Na]^+^ calculated for C_33_H_46_O_5_SiNa^+^ 573.3007; found 573.3013; ^1^H NMR (400.1 MHz, CDCl_3_, δ, ppm): 1.01–1.15 (m, 21H), 2.50 (m, 1H), 3.14–3.25 (m, 1H), 3.65 (m, 2H), 3.76 (m, 2H), 3.79 (s, 6H), 6.82 (m, 4H), 7.16–7.18 (m, 1H), 7.27 (m, 2H), 7.31 (m, 4H), 7.42 (m, 2H); ^13^C NMR (100.6 MHz, CDCl_3_, δ, ppm): 11.8, 17.9, 55.2, 64.2, 64.5, 71.2, 86.1, 113.1, 126.6, 127.7, 128.1, 130.0, 136.1, 144.9, 158.4. **1-(bis(4-methoxyphenyl)(phenyl)methoxy)-3-((triisopropylsilyl)oxy)propan-2-yl (2-cyanoethyl) diisopropylphosphoramidite M4**. In total, 2050 mg of **13** (3.7 mmol, 1.0 eq.) were dissolved in 10 mL anhydrous DCM. Then, DIPEA (4 mL, 6.0 eq.), molecular sieves (3 Å) and 2-cyanoethyl-*N,N*-diisopropylchlorophosphoramidite (970 mg, 4.08 mmol, 1.10 eq.) were added under argon atmosphere at 0 °C. The reaction mixture was stirred at 0 °C for 30 min and then allowed to reach RT. DCM was removed and the residue was purified by column chromatography (SiO_2_ vs 33 v% EtOAc in cyclohexane + 2 v% DIPEA) to obtain **M4** as a colorless foam in 95% yield. HRMS *m/z*: [M + H]^+^ calculated for C_42_H_64_N_2_O_3_PSi^+^ 751.4266; found 751.4279; ^1^H NMR (400.1 MHz, CDCl_3_, δ, ppm): 0.96–1.02 (m, 21H), 1.04–1.20 (m, 12H), 2.47–2.60 (m, 2H), 3.58–3.68 (m, 2H), 3.69–3.89 (m, 4H), 3.76 (m, 2H), 3.78 (s, 6H), 4.02 (m, 1H), 6.78–6.82 (m, 4H), 7.17–7.22 (m, 1H), 7.24–7.28 (m, 2H), 7.32–7.36 (m, 4H), 7.44–7.48 (m, 2H); ^13^C NMR (100.6 MHz, CDCl_3_, δ, ppm): 11.86, 17.10, 17.97, 20.67, 24.53, 24.60, 39.15, 43.23, 48.57, 55.18, 64.02, 74.59, 112.96, 126.5, 126.6, 127.6, 128.2, 128.3, 130.1; ^31^P NMR (161.9 MHz, CDCl_3_, δ, ppm): 149.3.

### Oligomer synthesis

Oligo(phosphodiester)s **H1**–**H3** and **P1**–**P13** were synthesized by solid-phase automated phosphoramidite chemistry on an Expedite oligonucleotide synthesizer (Perseptive Biosystem 8900), as previously reported^29^. **H1**–**H2** and **P1**–**P5** were synthesized using **M1** (0-bit) and **M2** (1-bit) as a binary alphabet (Supplementary Table 1). **H3** and **P6**–**P11** were synthesized using **M1** and **M3** as an invisible binary ink (Supplementary Table 2). **P12** and **P13** were synthesized using **M1**, **M3**, and **M4** as molecular alphabet (Supplementary Table 3). All the phosphoramidite monomers were dissolved in anhydrous acetonitrile (100 mM solution) under argon, placed in the synthesizer with all the reagents and primed twice. The solid support-filled column (1 μmol scale) was placed in the synthesizer and the automated syntheses of the different sequences were started with DMT-ON mode, using the standard protocol except for the sequences containing the monomer **M4**, for which the coupling of the corresponding phosphoramidite was repeated twice. The DMT-protected polymers were cleaved from the solid support using a 1:1 (v:v) solution of 28 w% ammonia and 40 w% methylamine: two syringes, one of which contains 0.5 mL, were placed at the two ends of the columns, pushed from one side, and pulled from the other for 1h. The cleaved polymers were collected in a microtube, the columns were afterwards washed with 0.5 mL of ammonia/methylamine solution, then collected in the same microtube, to which a volume of 1 mL of 100 mg/mL sodium chloride solution was added. The DMT-protected polymers were then purified on PolyPak II reverse-phase cartridges, which had been previously flushed with 0.5 mL MeCN and then with 1 mL of 2 M triethylammonium acetate (TEAA) buffer. For all polymers except **P12** and **P13**, the polymer/salt mixtures were loaded on the cartridges and the failure sequences were washed with twice 0.5 mL of a salt wash solution (5% MeCN/100 mg NaCl/1 mL water). The cartridges were then washed with twice 1 mL of aqueous 2% TFA to remove the last protecting DMT group and twice 1 mL of deionized water. The polymers were washed out with 1 mL of 50% MeCN in water containing 0.5% ammonium hydroxide, then collected in a microtube and lyophilized. In all cases, the sequences were obtained as triethylammonium salts. The formed oligomers were characterized by ^1^H NMR, ^31^P NMR, HPLC, ESI-HRMS and MS/MS. Yields: 99.2% (**H1**), 78.3% (**H2**), 99.2% (**H3**), 89.3% (**P1**), 96.0% (**P2**), 87.9% (**P3**), 96.0% (**P4**), 99.2% (**P5**), 90.0% (**P6**), 85.7% (**P7**), 82.3% (**P8**), 93.0% (**P9**), 91.5% (**P10**), 82.3% (**P11**), 97.6% (**P12**), 53.0% (**P13**). Purity from HPLC: 88% (**H1**), 92% (**H2**), 90% (**H3**), 91% (**P1**), 90% (**P2**), 92% (**P3**), 95% (**P4**), 93% (**P5**), 92% (**P6**), 91% (**P7**), 87% (**P8**), 92% (**P9**), 92% (**P10**), 92% (**P11**), 81% (**P12**), and 85% (**P13**).

### TIPS deprotection for polymers containing M4

The DMT-protected copolymer **P12** was dissolved in 115 μL DMSO, then 60 μL of Et_3_N was added and the solution was mixed gently. Afterwards, 75 μL of Et_3_N.3HF was added and the mixture was heated at 65 °C for 2.5 h. The deprotected polymer solution was cooled, and then 1.75 mL of aq. sat. NH_4_Cl solution was added. The mixture was loaded on PolyPak cartridge previously treated with 0.5 mL MeCN and 1 mL of 2 M TEAA buffer and purified as described above in the section 'Oligomer synthesis'.

### Photomodification

Polymers **H1–H3** and **P1–P13** were photo-irradiated, thus resulting in polymers **H1′**–**H3′** and **P1′**–**P13′** (Supplementary Tables 1–3). The general procedure is as follows: the oligo(phosphodiester) was dissolved in methanol-d4 (1.0 mM) under argon atmosphere. Then, 100 µL of the formed solution were filled into a small glass vial under exclusion of air and this vial was placed on the glass surface of a UV lamp (Vilber Lourmat; *λ* = 365 nm). The solution was irradiated for 60 min in a closed box and the solvent was afterwards removed to recover the modified sequences. The modified oligomers were characterized by ^1^H NMR, ^31^P NMR, ESI-HRMS and MS/MS.

### Nuclear magnetic resonance spectroscopy

All NMR spectra were recorded on a Bruker Avance 400 spectrometer equipped with an Ultrashield magnet. Chemical shifts (*δ*) are reported in parts per million (ppm) against solvent residual signal (^1^H NMR, CDCl_3_: *δ* = 7.26 ppm; ^13^C NMR, CDCl_3_: *δ* = 77.16 ppm; ^1^H NMR, CD_3_OD: *δ* = 3.31 ppm; ^13^C NMR, CD_3_OD: *δ* = 49.00 ppm.) ^1^H NMR spectra were recorded at 400.13 MHz, ^13^C NMR spectra at 100.62 MHz. The NMR solvents deuterated chloroform (99.8%, chloroform-d1) and deuterated methanol (99.8%, methanol-d4) were purchased from Aldrich.

### High-performance liquid chromatography

Ion exchange HPLC (IE-HPLC) analyses were performed using Agilent 1220 Infinity LC system with manual injector and single wavelength UV detector. The system was equipped with ion-exchange column (DIONEX, DNA Pac PA 100, 4 × 250 mm). The chromatograms were recorded at *λ* = 260 nm. Experimental conditions: phase A: 10% MeCN 20% 2 M NH_3_ in water, phase B: 2.5 M NaCl in water; gradient elution: 0–3 min 5% B, 3–23 min from 5% B to 30% B, 23–28 min 30% B, 28–35 min from 30 to 5% B; flow rate: 1 mL/min. For IE-HPLC analysis MilliQ water, NaCl (BioXtra, ≥ 99.5%, Sigma-Aldrich) and acetonitrile (ACN, HPLC grade, ≥99.93%, Sigma-Aldrich) were used. Oligomer purity was calculated based on signal area integrals using formula:

$${\mathrm{{Purity}}}\,[{\mathrm{\% }}] = \frac{{I_{\mathrm{P}} \times 100}}{{{\sum} {I_n} }},$$

where *I*_P_ is the surface area of the signal representing product and ${\sum} {I_n}$ is a sum of signals areas of other signals present in the HPLC chromatogram occurring in range 5–35 min of elution time. The integrations were calculated in OpenLab software using automatic integration mode.

### Electrospray ionization mass spectrometry

High-resolution MS and MS/MS experiments were performed using a QStar Elite mass spectrometer (Applied Biosystems SCIEX, Concord, ON, Canada) equipped with an electrospray ionization (ESI) source operated in the negative mode. The capillary voltage was set at –4200 V and the cone voltage at –75 V. In this hybrid instrument, ions were measured using an orthogonal acceleration time-of-flight (oa-TOF) mass analyzer. In the MS/MS mode, a quadrupole was used for selection of precursor ions to be further submitted to collision-induced dissociation (CID) in a collision cell. Accurate mass measurements were performed using internal calibration. In this instrument, air was used as the nebulizing gas (10 psi) while nitrogen was used as the curtain gas (20 psi) as well as the collision gas. Instrument control, data acquisition, and data processing of all experiments were achieved using Analyst software (QS 2.0) provided by Applied Biosystems. Polymer solutions were prepared in methanol supplemented with ammonium acetate (3 mM) and introduced in the ionization source using a syringe pump (flow rate: 10 μL/min). Dissociation reactions proceed via cleavages of backbone bonds of the poly(phosphodiester) chains that give rise to α-containing (i.e., a_i_^z−^, b_i_^z−^, c_i_^z−^, d_i_^z−^) or ω-containing (i.e., w_i_^z−^, x_i_^z−^, y_i_^z−^, z_i_^z−^) fragments typically employed to reconstruct the sequence from the left- or from the right-hand side of the chain, respectively.

## Supplementary information

Supplementary Information

## Data Availability

All data generated and analyzed during this study are included in this article and its Supplementary Information, and are also available from the authors upon reasonable request.
