# Supplementary material for: Photo-editable macromolecular information
Source: Nat Commun. 2019 Sep 4;10:3774. doi: 10.1038/s41467-019-11566-2 (PMC6726599; doi:10.1038/s41467-019-11566-2)
Supplement: Supplementary file 1 — Supplementary Information [file 41467_2019_11566_MOESM1_ESM.pdf]

Supplementary Figures.

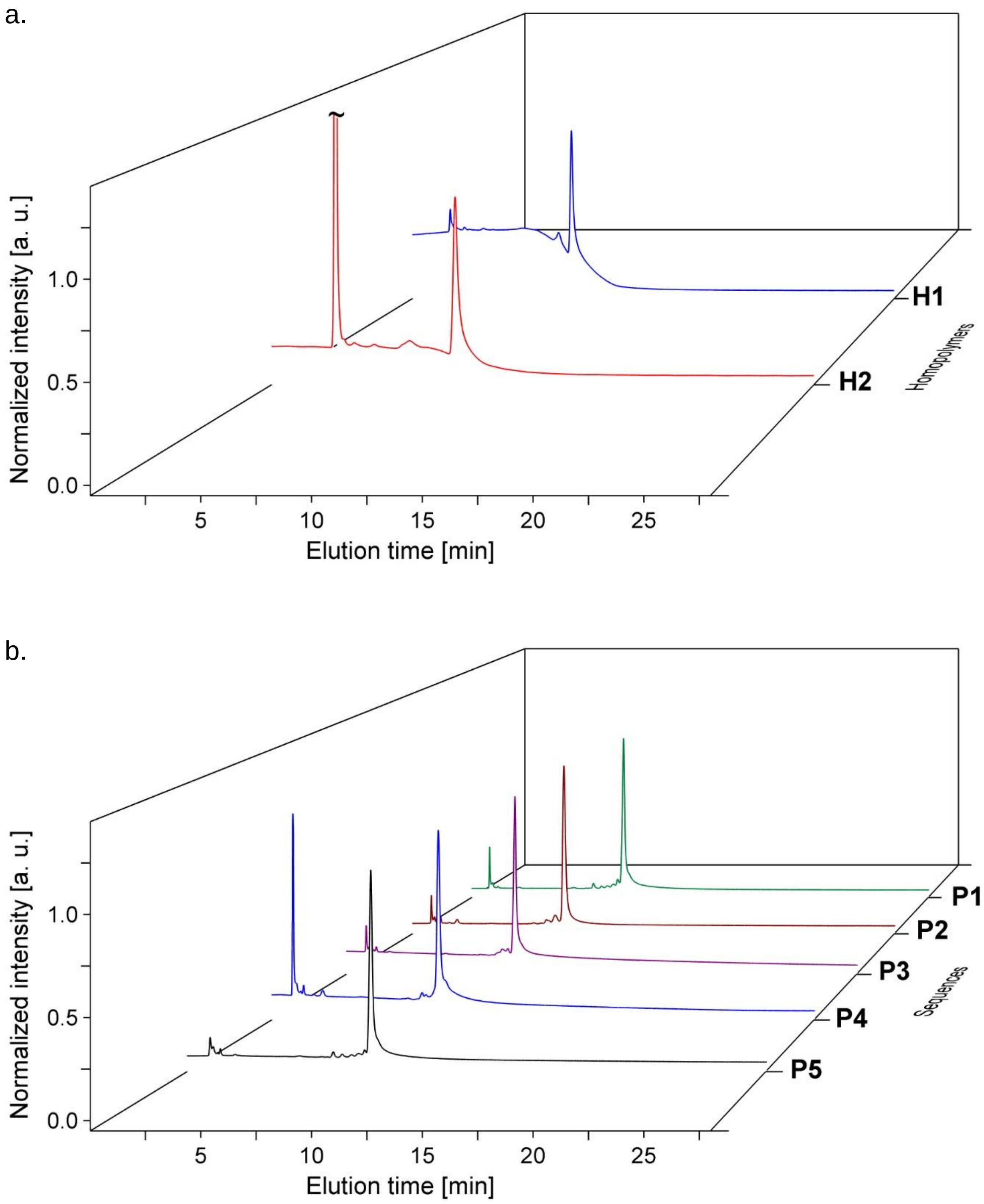

Supplementary Figure 1. IE-HPLC traces measured for (**a**) homopolymers **H1-H2** and (**b**) copolymers **P1-P5**. The chromatograms are recorded at λ =260 nm. Experimental conditions: phase A: 10 % MeCN 20 % 2M NH_3 in water, phase B: 2.5 M NaCl in water; gradient: 0-3 min 5 % B, 3-23 min 5 % B-30 % B, 23-28 min 30 %B, 28-35 min 30%→5 % B; flow rate: 1 mL·min^{-1}.

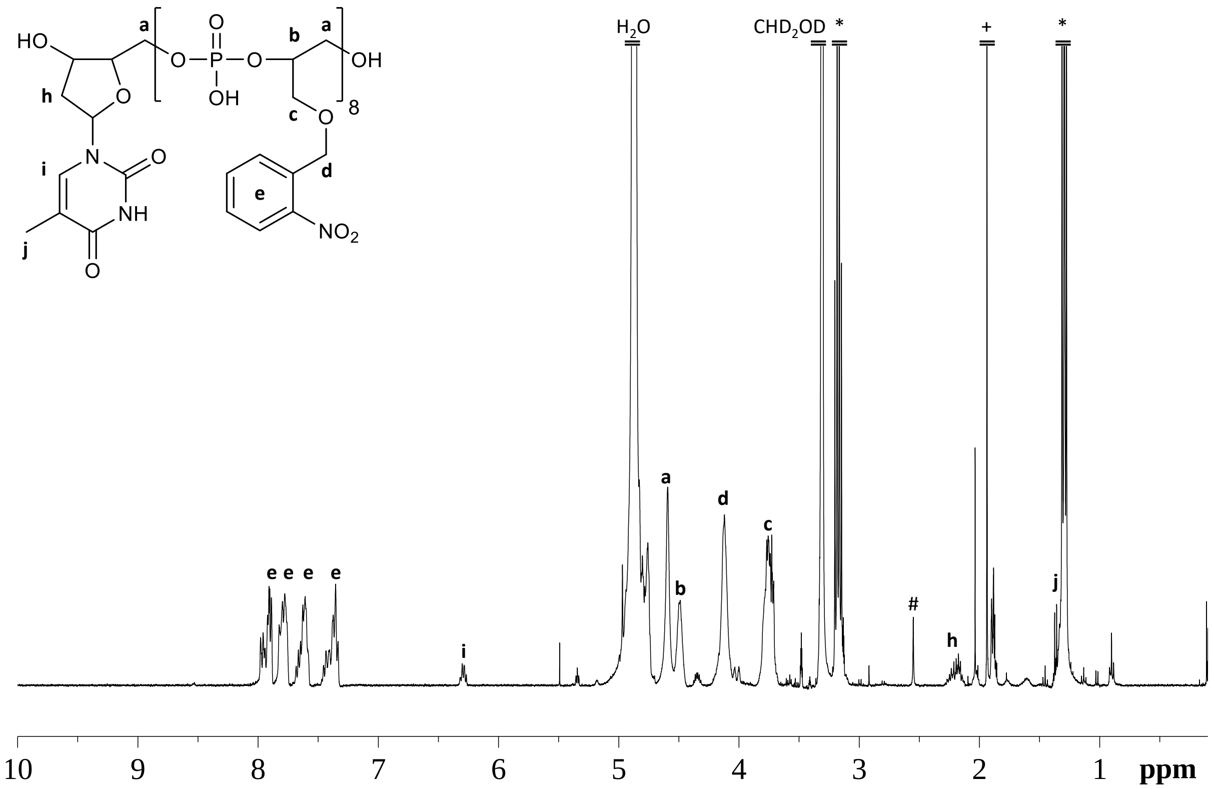

Supplementary Figure 2. ^{1}H NMR spectrum recorded at RT in CD3OD for homopolymer **H1**. The star symbols indicate signals of triethylammonium counterions. (#) Methylamine, (+) acetonitrile.

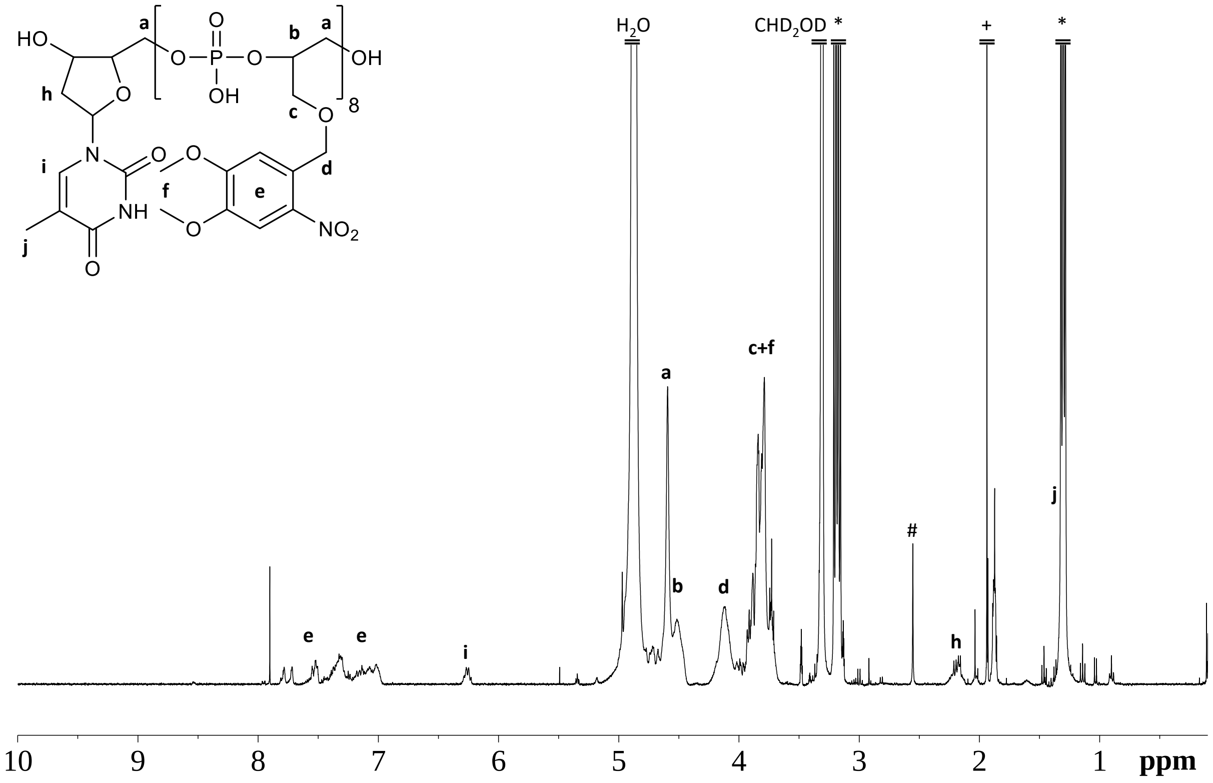

Supplementary Figure 3. ^{1}H NMR spectrum recorded at RT in CD3OD for homopolymer **H2**. The star symbols indicate signals of triethylammonium counterions. (#) Methylamine, (+) acetonitrile.

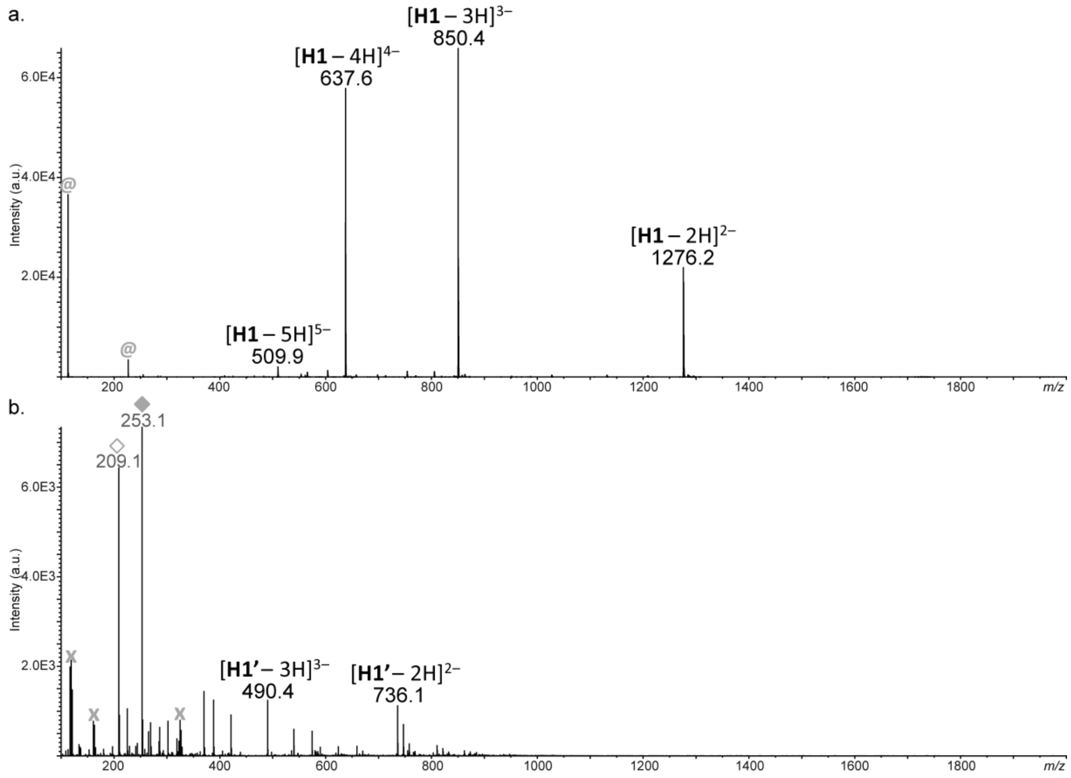

Supplementary Figure 4. ESI HRMS spectra recorded for (**a**) homopolymer **H1** before irradiation and (**b**) the resulting polymer **H1’** after photo-exposure. Grey symbols designate clusters of trifluoroacetic acid (@), of trichloroacetic acid (x) or photo-deprotection by-products (diamond).

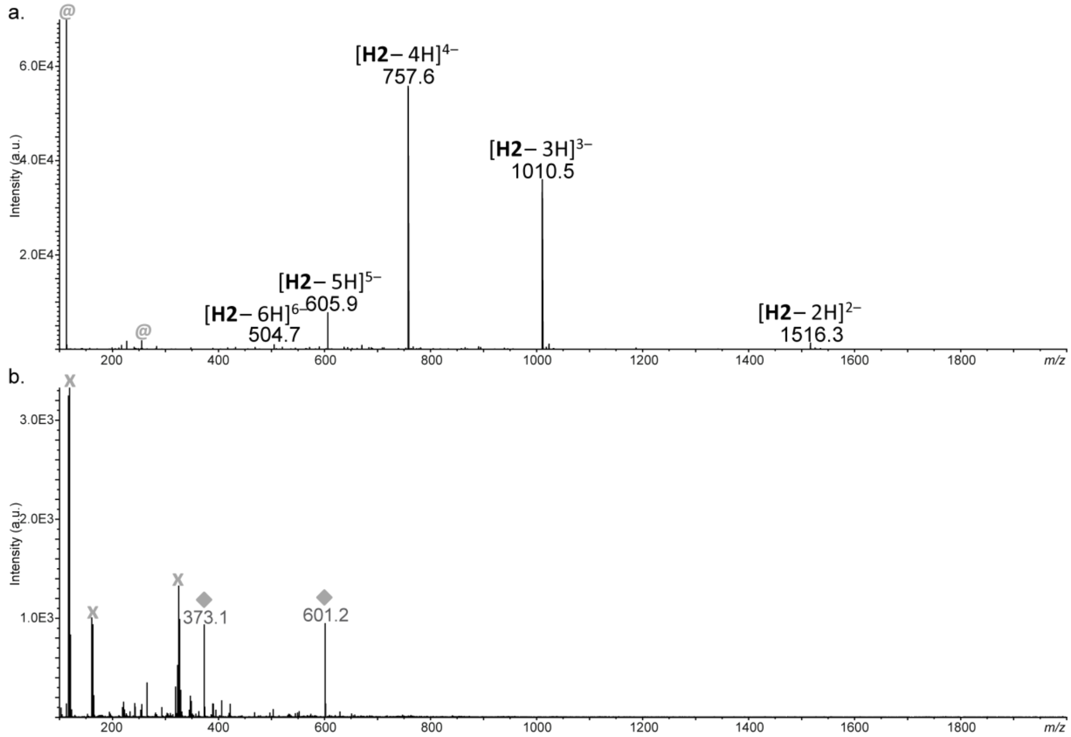

Supplementary Figure 5. ESI HRMS spectra recorded for (**a**) homopolymer **H2** before irradiation and (**b**) the resulting polymer **H2’** after photo-exposure. Grey symbols designate clusters of trifluoroacetic acid (@), of trichloroacetic acid (x) or photo-deprotection by-products (diamond).

Supplementary discussion.

Interpretation of the photo-deprotection by-products detected by ESI HRMS. The peaks indicated by filled grey diamonds in ESI HRMS spectra of photo-exposed samples correspond to photo-deprotection by-products. As indicated in Fig. 1 of the main manuscript, nitrosobenzaldehyde intermediates are usually formed upon *o*-nitrobenzyl and *o*-nitroveratryl photocleavage.[1] However, it has been reported that these intermediates can further photo-dimerize into azobenzene diacid derivatives.[2,3] In the present photo-deprotection and analytical conditions, the formation of dimers containing carboxylic acid and aldehyde groups was observed,[4] as evidenced below in Supplementary Figures 6 and 7.

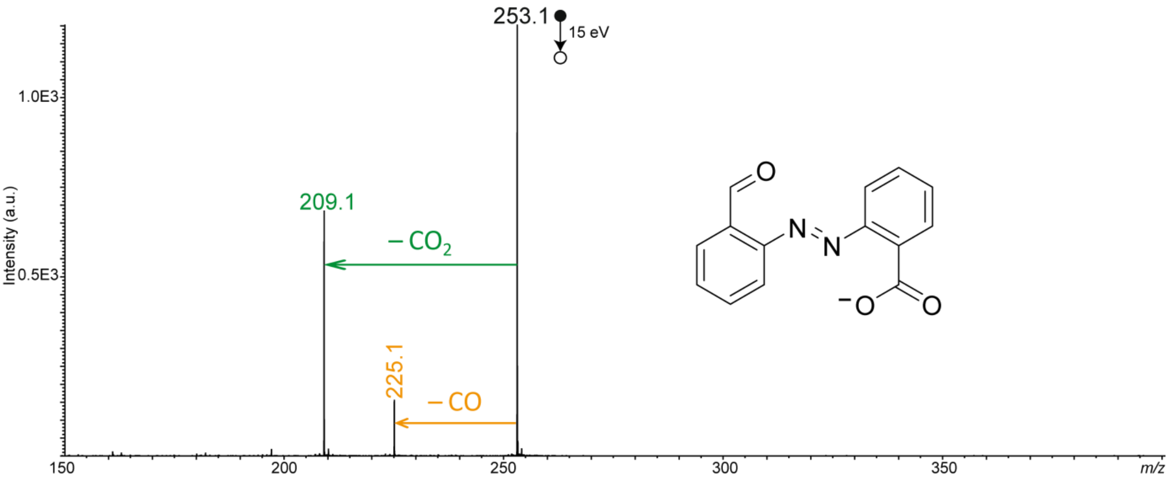

Supplementary Figure 6. MS/MS spectrum recorded for the photo-deprotection by-product observed at *m/z* 253.1 ($C_{14}H_9N_2O_3^-$, m/z_{th} 253.0619, m/z_{exp} 253.0614) after photo-irradiation of homopolymer **H1**, showing competitive losses of CO and CO_2 consistent with the proposed structure shown in inset. Of note, the *m/z* 209.1 ion labelled with an empty diamond in Supplementary Figure 4 indicates that the decarboxylation reaction also occurred during ion transfer through the interface of the mass spectrometer.

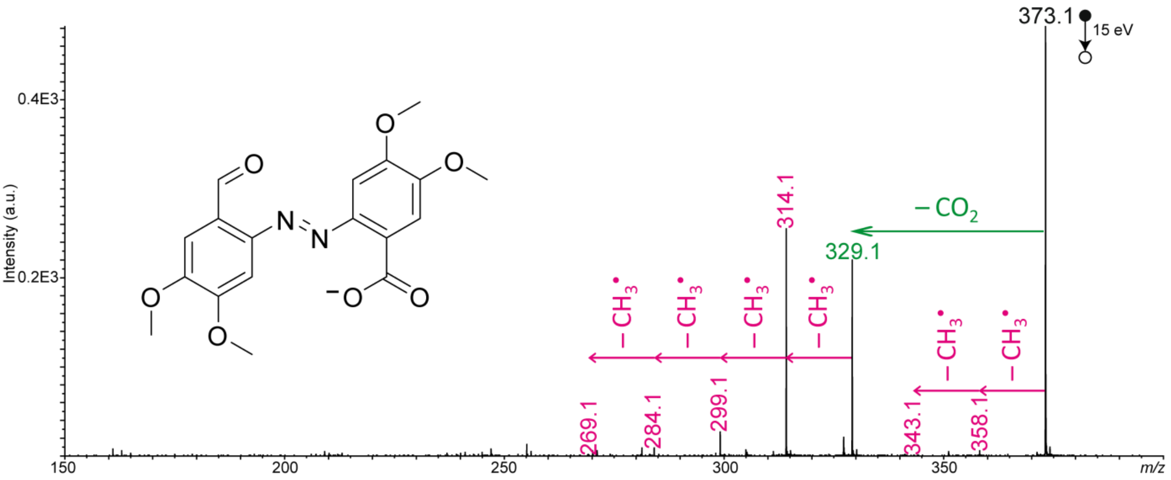

Supplementary Figure 7. MS/MS spectrum recorded for the photo-deprotection by-product observed at *m/z* 373.1 ($C_{18}H_{17}N_2O_7^-$, m/z_{th} 373.1041, m/z_{exp} 373.1022) after photo-irradiation of homopolymer **H2**, showing that the decarboxylation product (*m/z* 329.1) successively eliminates four methyl radicals, consistent with the four methoxy groups of the precursor ion (see inset structure).

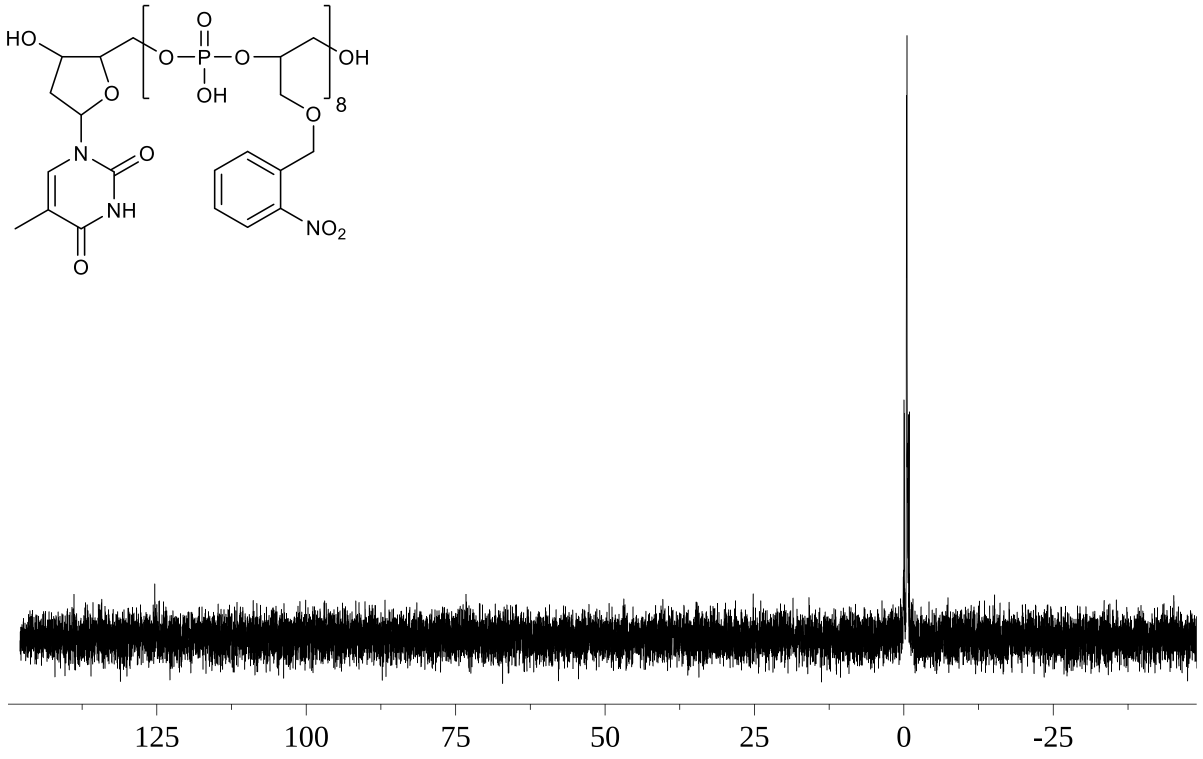

Supplementary Figure 8. ^{31}P NMR spectrum recorded at RT in CD_3OD for homopolymer **H1**. Comparable spectra were obtained for all other homopolymers studied in this work.

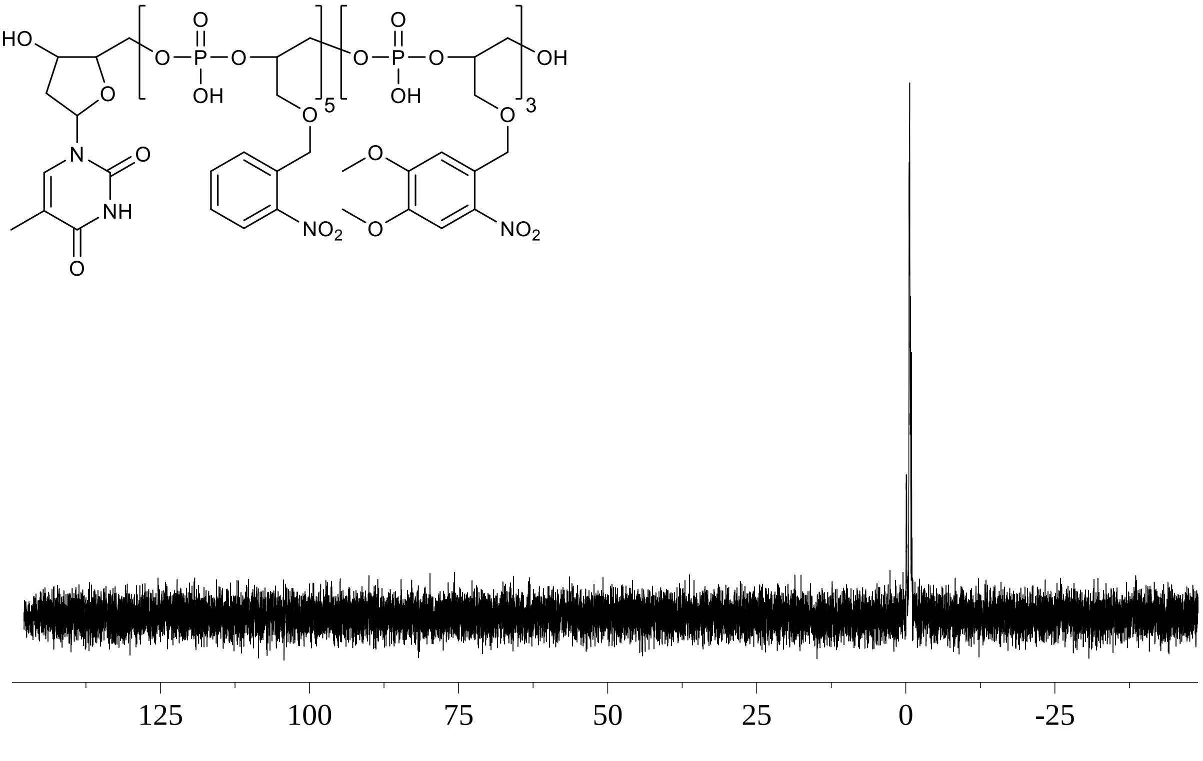

Supplementary Figure 9. ^{31}P NMR spectrum recorded at RT in CD_3OD for copolymer **P1**. Comparable spectra were obtained for all other copolymers studied in this work.

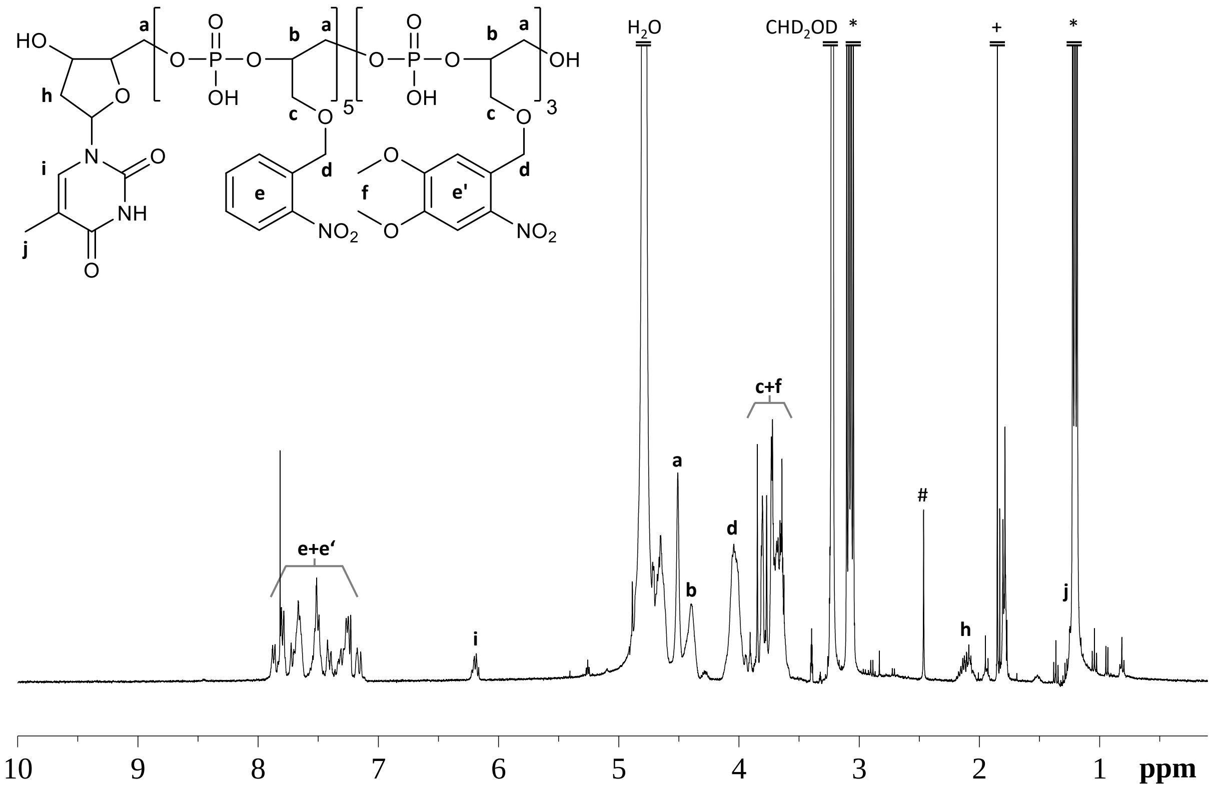

Supplementary Figure 10. ^{1}H NMR spectrum recorded at RT in CD_3OD for copolymer **P1**. The star symbols indicate signals of triethylammonium counterions. (#) Methylamine, (+) acetonitrile.

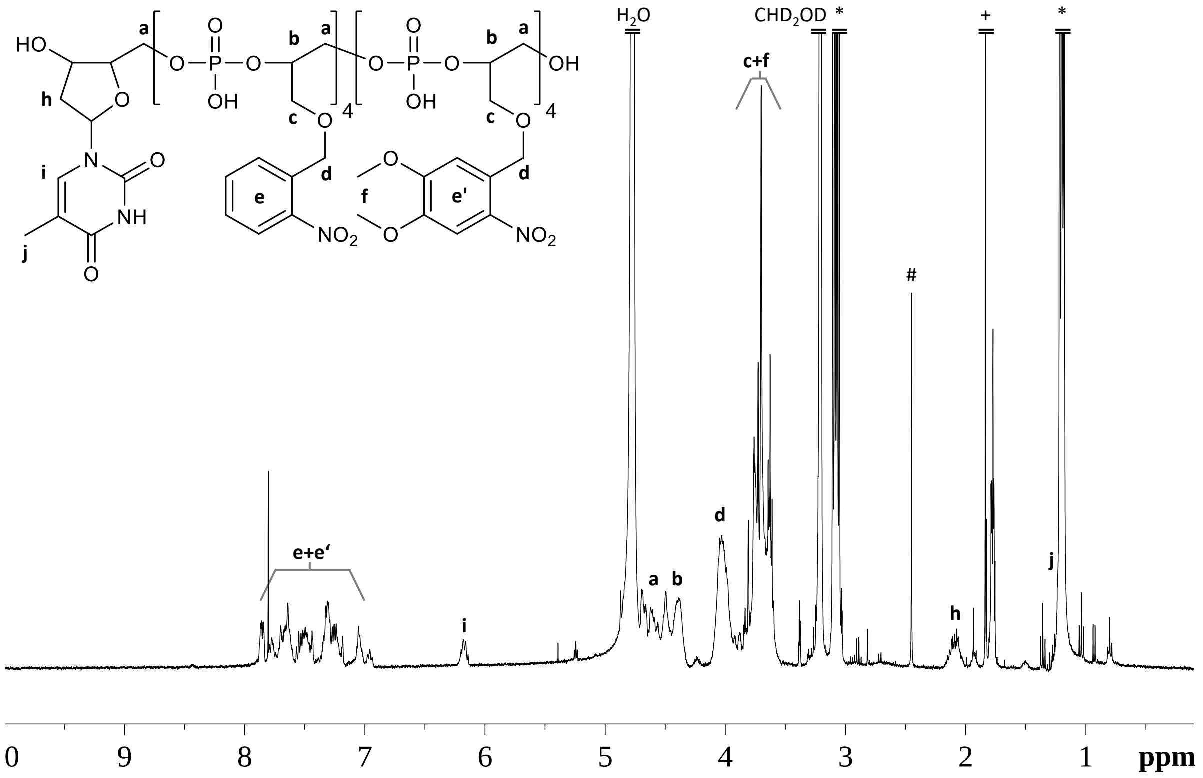

Supplementary Figure 11. ^{1}H NMR spectrum recorded at RT in CD_3OD for copolymer **P2**. The star symbols indicate signals of triethylammonium counterions. (#) Methylamine, (+) acetonitrile.

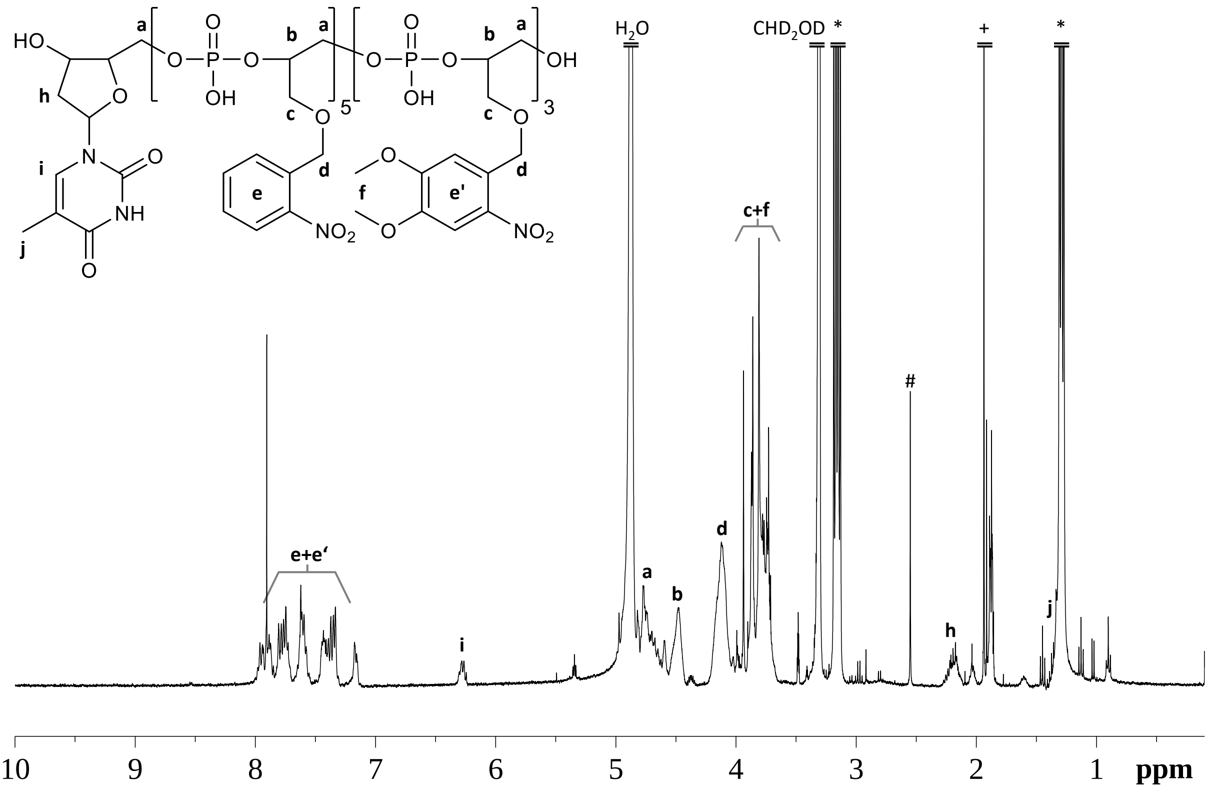

Supplementary Figure 12. ^{1}H NMR spectrum recorded at RT in CD_3OD for copolymer **P3**. The star symbols indicate signals of triethylammonium counterions. (#) Methylamine, (+) acetonitrile.

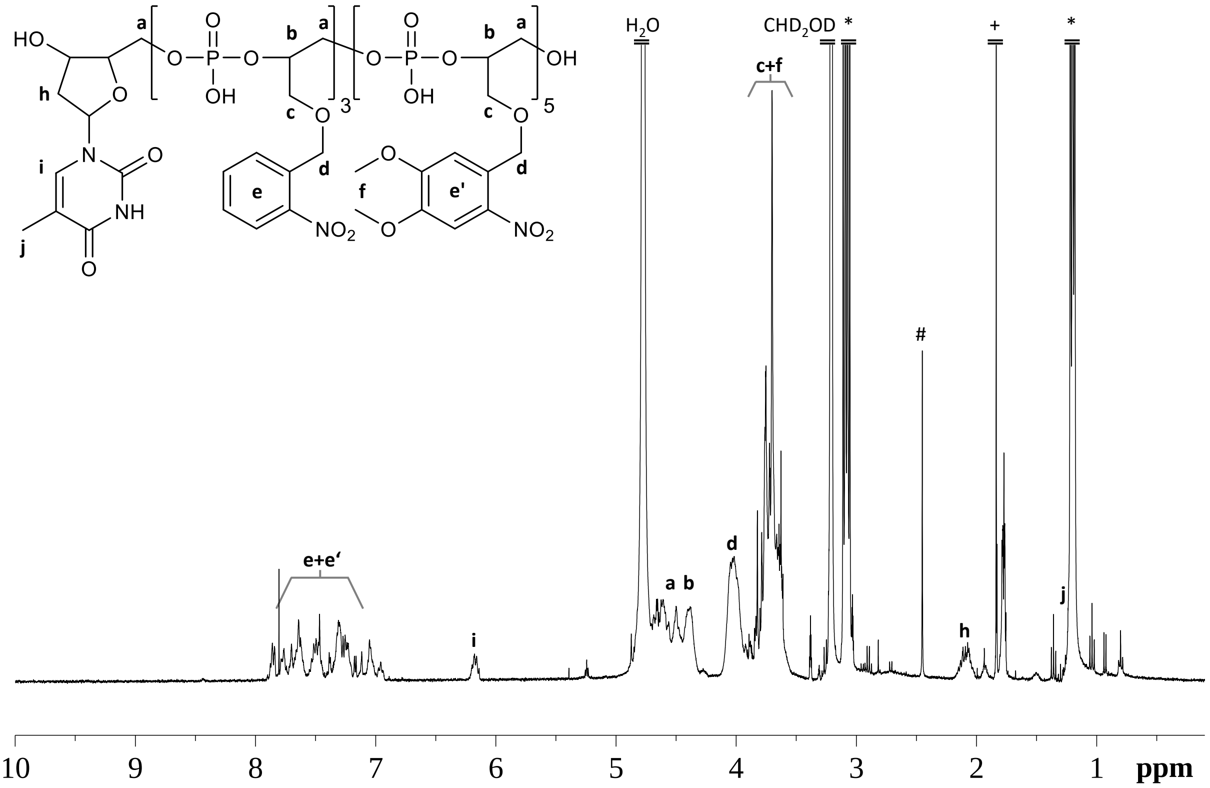

Supplementary Figure 13. ^{1}H NMR spectrum recorded at RT in CD_3OD for copolymer **P4**. The star symbols indicate signals of triethylammonium counterions. (#) Methylamine, (+) acetonitrile.

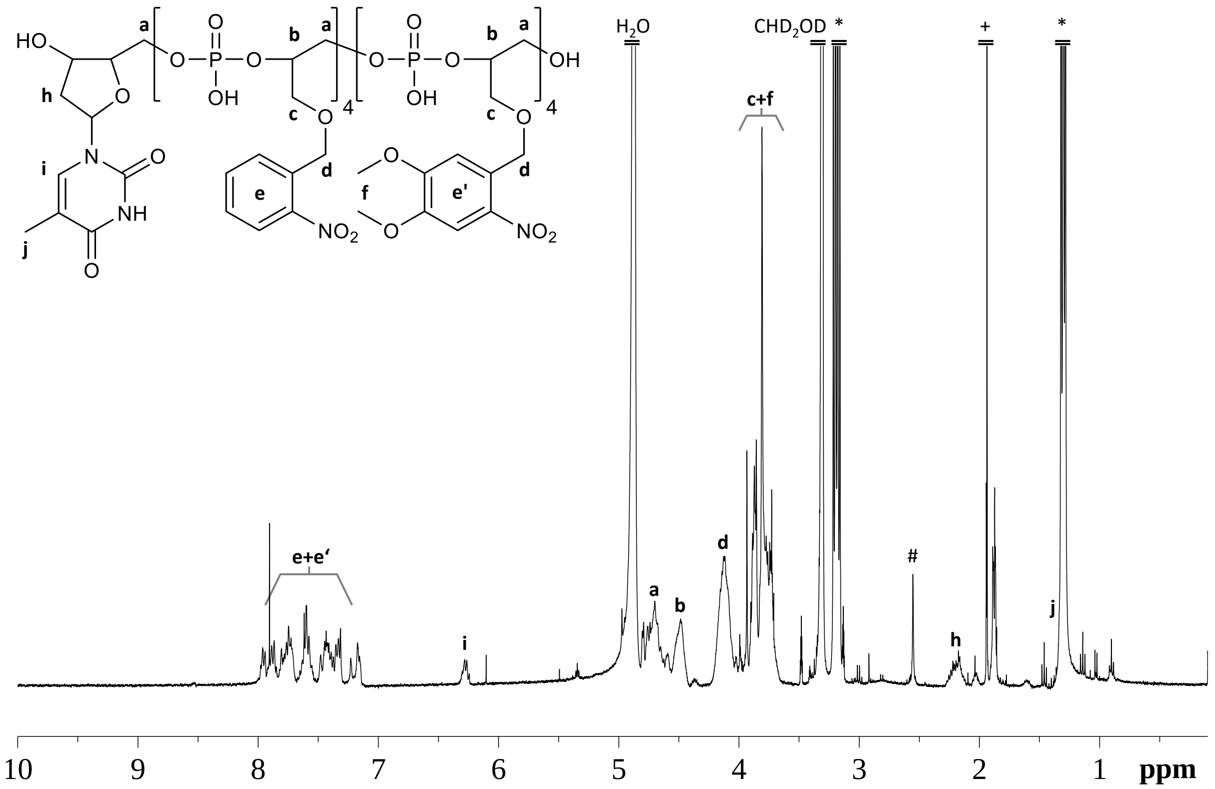

Supplementary Figure 14. ^1H NMR spectrum recorded at RT in CD_3OD for copolymer **P5**. The star symbols indicate signals of triethylammonium counterions. (#) Methylamine, (+) acetonitrile.

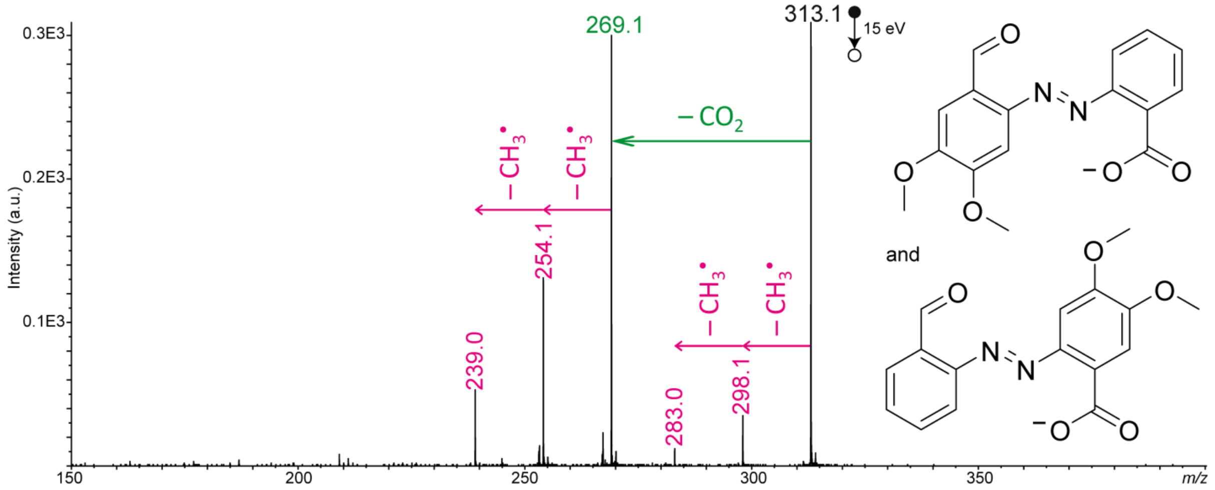

Supplementary Figure 15. MS/MS spectrum recorded for the photo-deprotection by-product observed at *m/z* 313.1 ($C_{16}H_{13}N_2O_5^-$, m/z_{th} 313.0830, m/z_{exp} 313.0835) after photo-irradiation of copolymer **P1**, showing that the decarboxylation product (*m/z* 269.1) successively eliminates two methyl radicals, consistent with the two methoxy groups in the isomeric precursors (see inset structure).

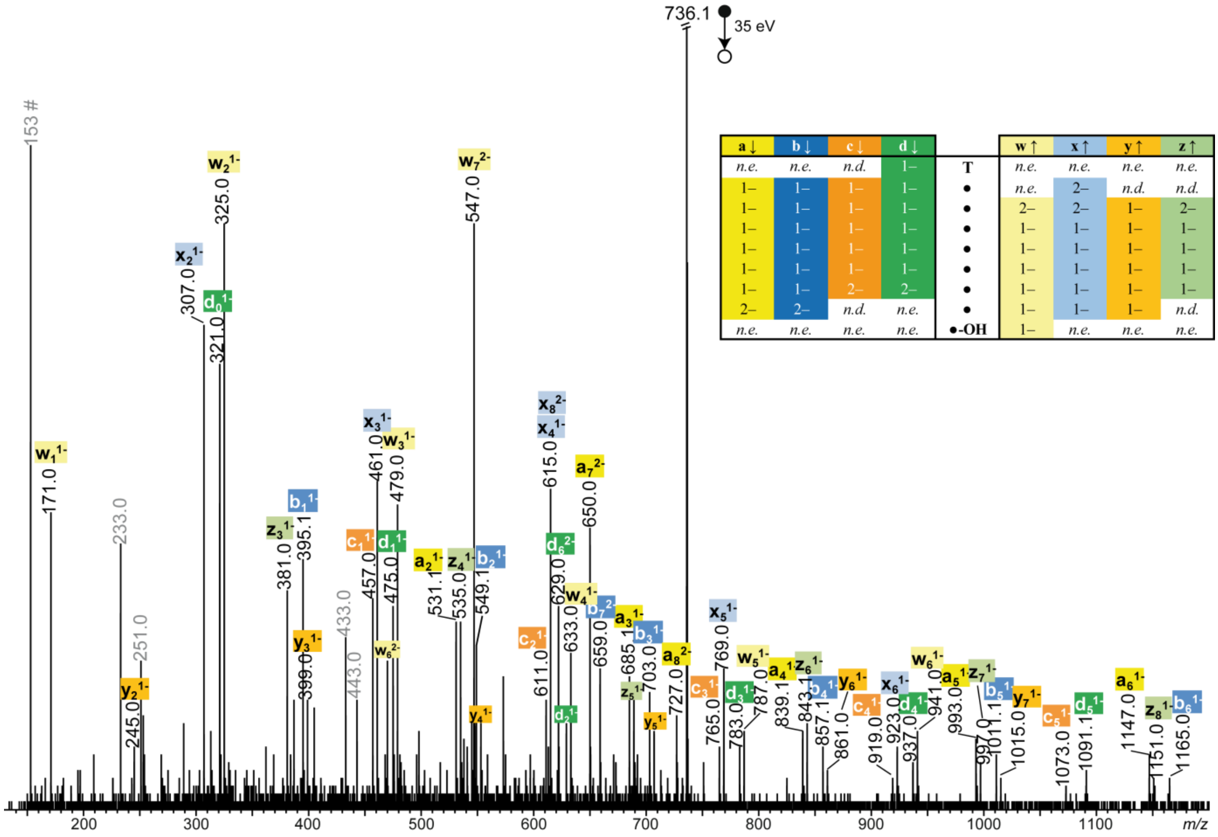

a↓	b↓	c↓	d↓		w↑	x↑	y↑	z↑
n.e.	*n.e.*	*n.d.*	1–	**T**	*n.e.*	*n.e.*	*n.e.*	*n.e.*
1–	1–	1–	1–	•	*n.e.*	2–	*n.d.*	*n.d.*
1–	1–	1–	1–	•	2–	2–	1–	2–
1–	1–	1–	1–	•	1–	1–	1–	1–
1–	1–	1–	1–	•	1–	1–	1–	1–
1–	1–	1–	1–	•	1–	1–	1–	1–
1–	1–	2–	2–	•	1–	1–	1–	1–
2–	2–	*n.d.*	*n.e.*	•	1–	1–	1–	*n.d.*
n.e.	*n.e.*	*n.e.*	*n.e.*	**•-OH**	1–	*n.e.*	*n.e.*	*n.e.*

Supplementary Figure 16. MS/MS spectrum recorded for copolymer **P1’** after photo-exposure, using a 35 eV collision energy (laboratory frame) to activate the doubly deprotonated precursor at *m/z* 736.1. Peaks annotated in grey correspond to secondary fragments, including the deprotonated unit (*m/z* 153.0) designated by #. The inset table shows that most expected members of the eight fragment series are detected and evidence the monotonic sequence of **P1’**. *n.d.*: not detected. *n.e.*: not expected.

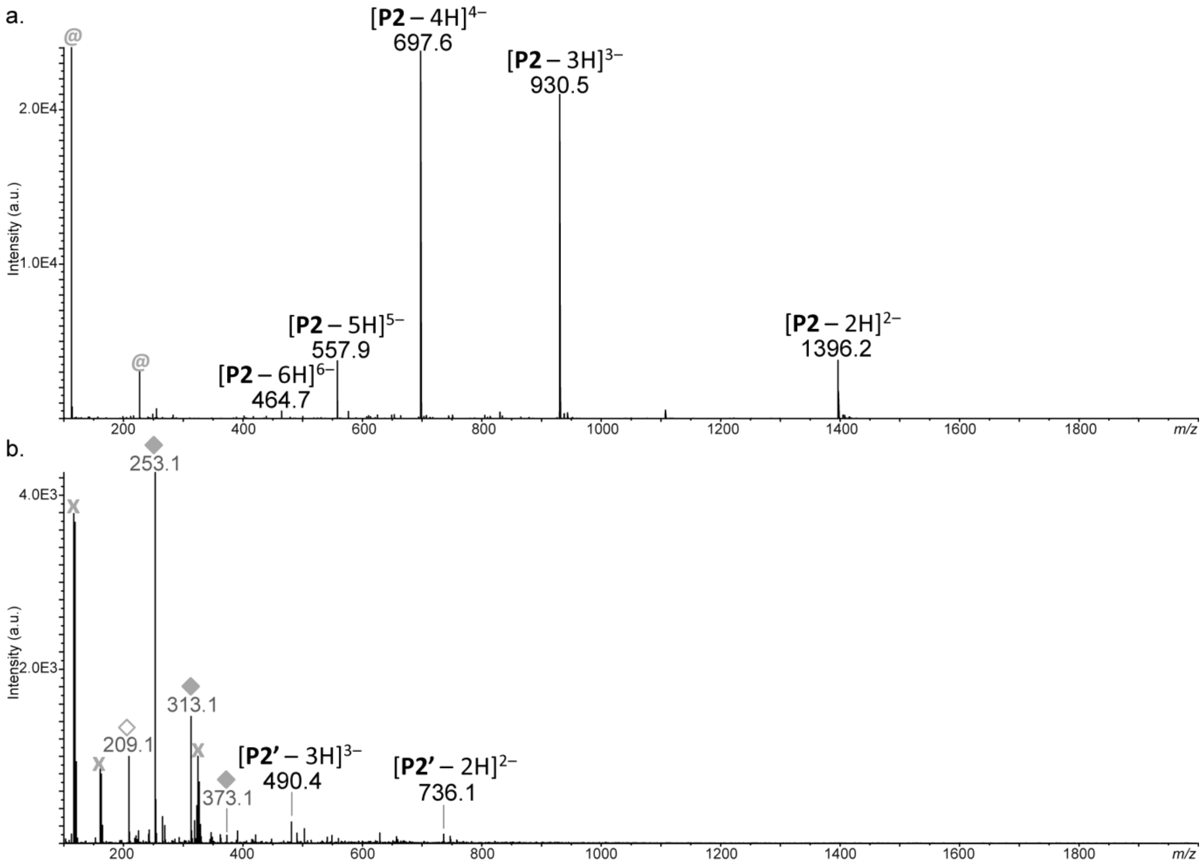

Supplementary Figure 17. ESI HRMS spectra recorded for (**a**) copolymer **P2** before irradiation and (**b**) the resulting polymer **P2'** after photo-exposure. Grey symbols designate clusters of trifluoroacetic acid (@), clusters of trichloroacetic acid (x) or photo-deprotection by-products (diamond).

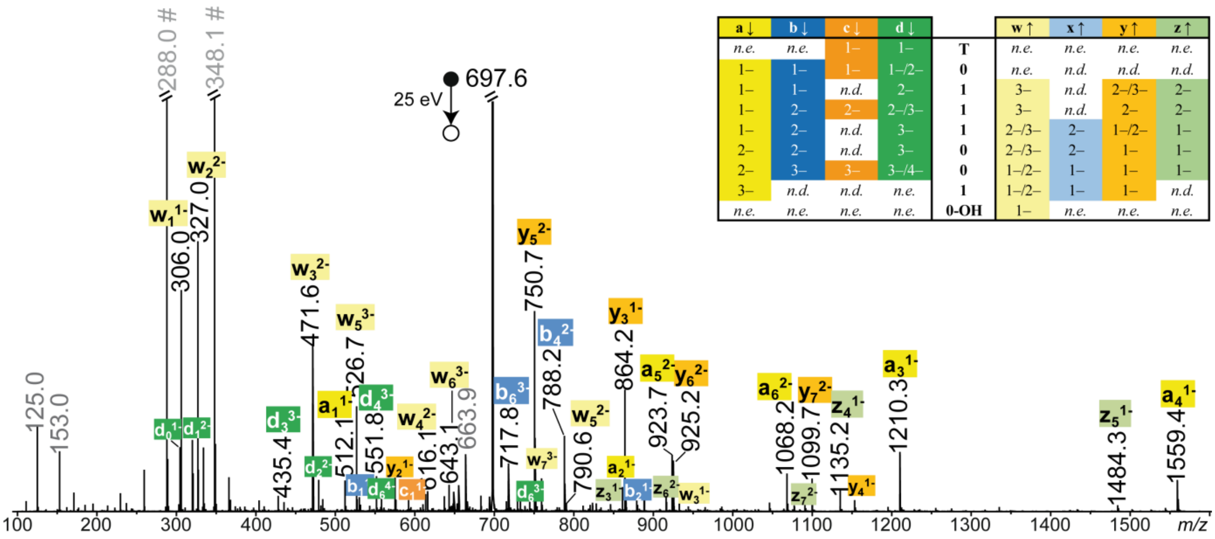

a↓	b↓	c↓	d↓		w↑	x↑	y↑	z↑
n.e.	*n.e.*	1–	1–	**T**	*n.e.*	*n.e.*	*n.e.*	*n.e.*
1–	1–	1–	1–/2–	**0**	*n.e.*	*n.d.*	*n.d.*	*n.d.*
1–	1–	*n.d.*	2–	**1**	3–	*n.d.*	2–/3–	2–
1–	2–	2–	2–/3–	**1**	3–	*n.d.*	2–	2–
1–	2–	*n.d.*	3–	**1**	2–/3–	2–	1–/2–	1–
2–	2–	*n.d.*	3–	**0**	2–/3–	2–	1–	1–
2–	3–	3–	3–/4–	**0**	1–/2–	1–	1–	1–
3–	*n.d.*	*n.d.*	*n.e.*	**1**	1–/2–	1–	1–	*n.d.*
n.e.	*n.e.*	*n.e.*	*n.e.*	**0-OH**	1–	*n.e.*	*n.e.*	*n.e.*

Supplementary Figure 18. MS/MS spectrum recorded for copolymer **P2** before irradiation, using a 25 eV collision energy (laboratory frame) to activate the quadruply deprotonated precursor at *m/z* 697.6. Peaks annotated in grey correspond to secondary fragments, including the deprotonated units, [0 – H]$^-$ at *m/z* 288.0 and [1 – H]$^-$ at *m/z* 348.1, both designated by #. Inset Table: Sequence coverage of **P2** (*n.d.*: not detected; *n.e.*: not expected). After photo-exposure, the resulting polymer **P2'** exhibits the same MS/MS spectrum as **P1'** in Supplementary Figure 16.

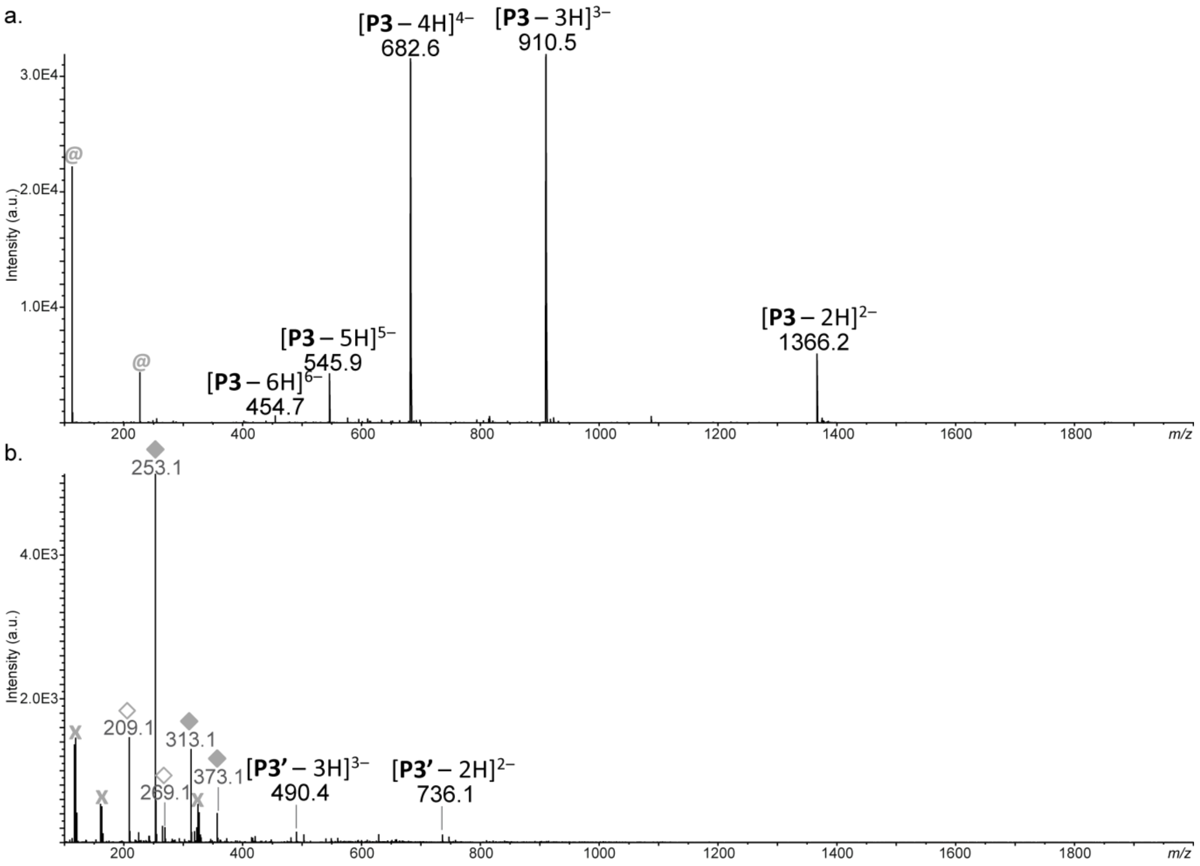

Supplementary Figure 19. ESI HRMS spectra recorded for (**a**) copolymer **P3** before irradiation and (**b**) the resulting polymer **P3’** after photo-exposure. Grey symbols designate clusters of trifluoroacetic acid (@), clusters of trichloroacetic acid (x) or photo-deprotection by-products (diamond).

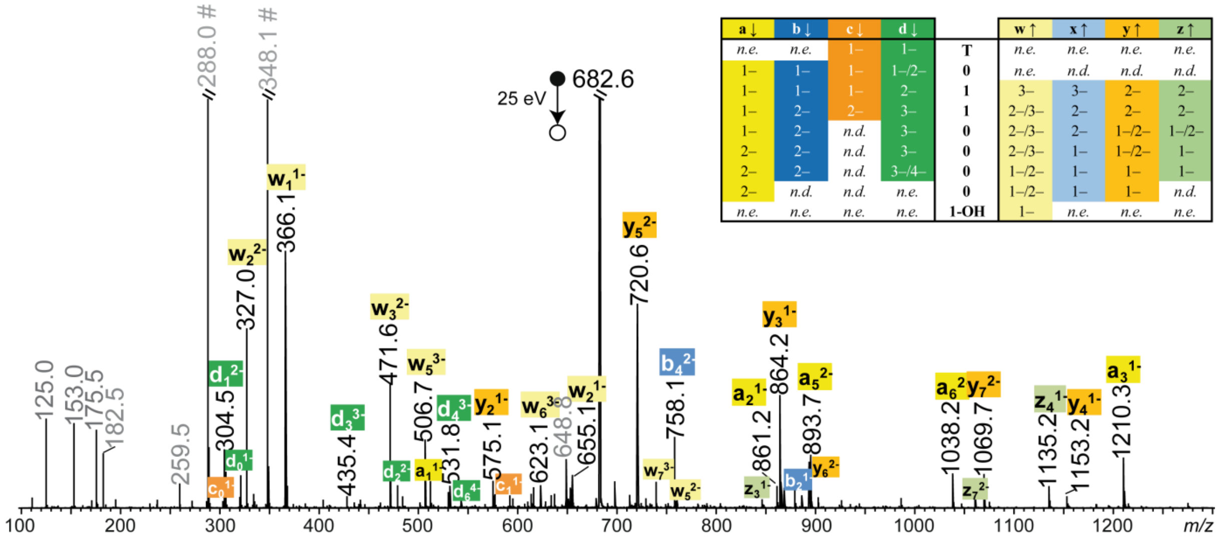

a↓	b↓	c↓	d↓		w↑	x↑	y↑	z↑
n.e.	*n.e.*	1–	1–	**T**	*n.e.*	*n.e.*	*n.e.*	*n.e.*
1–	1–	1–	1–/2–	**0**	*n.e.*	*n.d.*	*n.d.*	*n.d.*
1–	1–	1–	2–	**1**	3–	3–	2–	2–
1–	2–	2–	3–	**1**	2–/3–	2–	2–	2–
1–	2–	*n.d.*	3–	**0**	2–/3–	2–	1–/2–	1–/2–
2–	2–	*n.d.*	3–	**0**	2–/3–	1–	1–/2–	1–
2–	2–	*n.d.*	3–/4–	**0**	1–/2–	1–	1–	1–
2–	*n.d.*	*n.d.*	*n.e.*	**0**	1–/2–	1–	1–	*n.d.*
n.e.	*n.e.*	*n.e.*	*n.e.*	**1-OH**	1–	*n.e.*	*n.e.*	*n.e.*

Supplementary Figure 20. MS/MS spectrum recorded for copolymer **P3** before irradiation, using a 25 eV collision energy (laboratory frame) to activate the quadruply deprotonated precursor at *m/z* 682.6. Peaks annotated in grey correspond to secondary fragments, including the deprotonated units, $[0 – H]^–$ at *m/z* 288.0 and $[1 – H]^–$ at *m/z* 348.1, both designated by #. Inset Table: Sequence coverage of **P3** (*n.d.*: not detected; *n.e.*: not expected). After photo-exposure, the resulting polymer **P3’** exhibits the same MS/MS spectrum as **P1’** in Supplementary Figure 16.

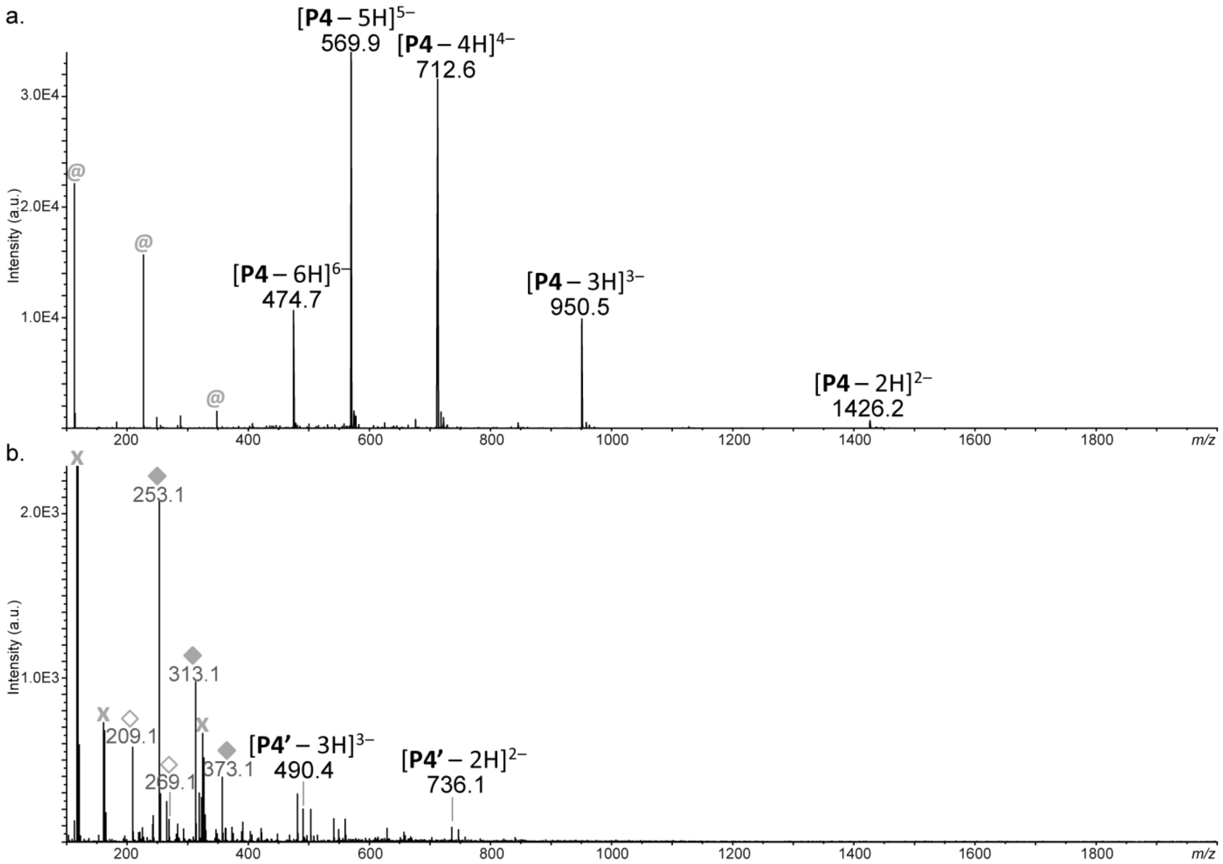

Supplementary Figure 21. ESI HRMS spectra recorded for (**a**) copolymer **P4** before irradiation and (**b**) the resulting polymer **P4'** after photo-exposure. Grey symbols designate clusters of trifluoroacetic acid (@), clusters of trichloroacetic acid (x) or photo-deprotection by-products (diamond).

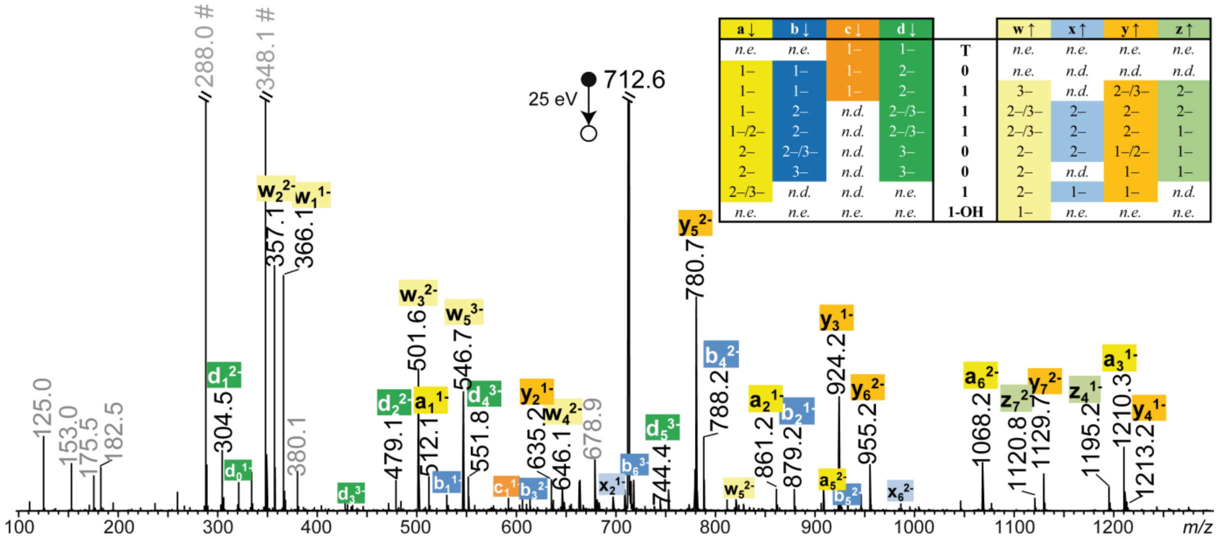

a↓	b↓	c↓	d↓		w↑	x↑	y↑	z↑
n.e.	*n.e.*	1–	1–	**T**	*n.e.*	*n.e.*	*n.e.*	*n.e.*
1–	1–	1–	2–	**0**	*n.e.*	*n.d.*	*n.d.*	*n.d.*
1–	1–	1–	2–	**1**	3–	*n.d.*	2–/3–	2–
1–	2–	*n.d.*	2–/3–	**1**	2–/3–	2–	2–	2–
1–/2–	2–	*n.d.*	2–/3–	**1**	2–/3–	2–	2–	1–
2–	2–/3–	*n.d.*	3–	**0**	2–	2–	1–/2–	1–
2–	3–	*n.d.*	3–	**0**	2–	*n.d.*	1–	1–
2–/3–	*n.d.*	*n.d.*	*n.e.*	**1**	2–	1–	1–	*n.d.*
n.e.	*n.e.*	*n.e.*	*n.e.*	**1-OH**	1–	*n.e.*	*n.e.*	*n.e.*

Supplementary Figure 22. MS/MS spectrum recorded for copolymer **P4** before irradiation, using a 25 eV collision energy (laboratory frame) to activate the quadruply deprotonated precursor at *m/z* 712.6. Peaks annotated in grey correspond to secondary fragments, including the deprotonated units, $[0 - H]^-$ at *m/z* 288.0 and $[1 - H]^-$ at *m/z* 348.1, both designated by #. Inset Table: Sequence coverage of **P4** (*n.d.*: not detected; *n.e.*: not expected). After photo-exposure, the resulting polymer **P4'** exhibits the same MS/MS spectrum as **P1'** in Supplementary Figure 16.

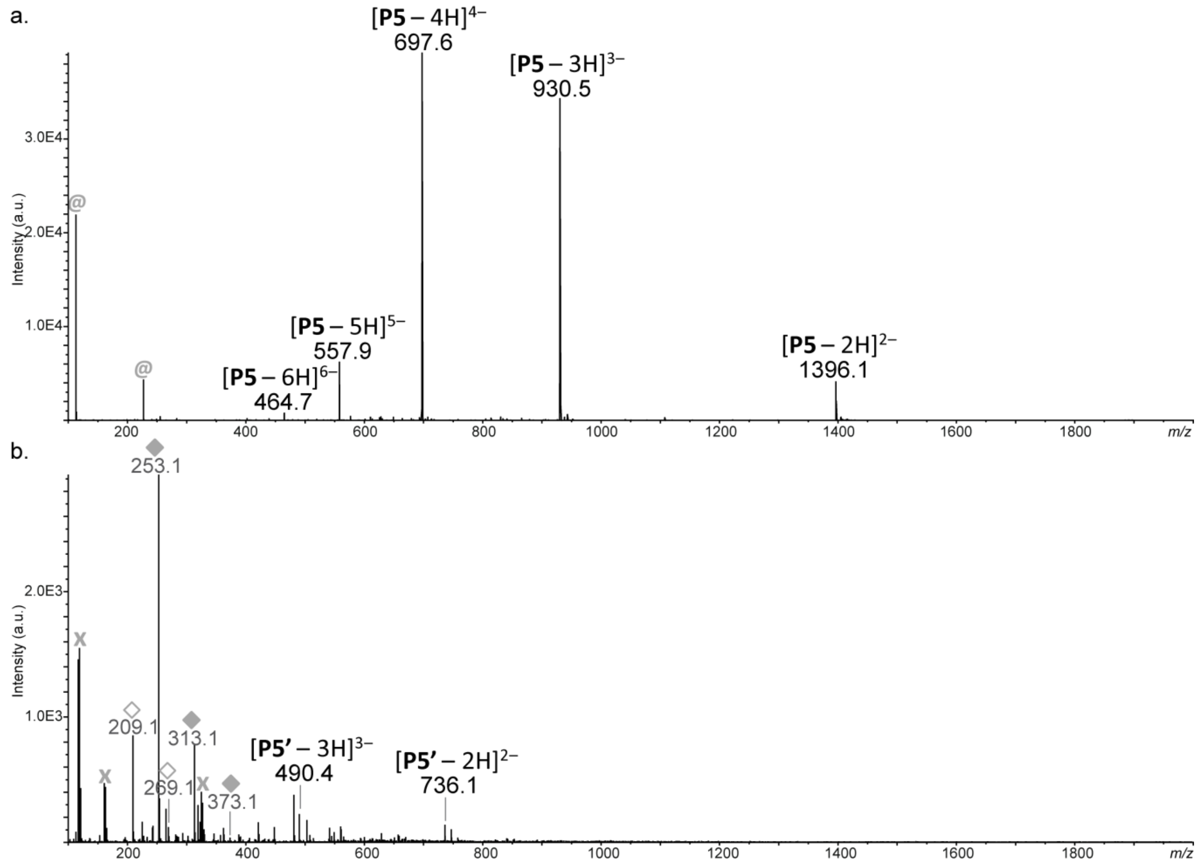

Supplementary Figure 23. ESI HRMS spectra recorded for (**a**) copolymer **P5** before irradiation and (**b**) the resulting polymer **P5’** after photo-exposure. Grey symbols designate clusters of trifluoroacetic acid (@), clusters of trichloroacetic acid (x) or photo-deprotection by-products (diamond).

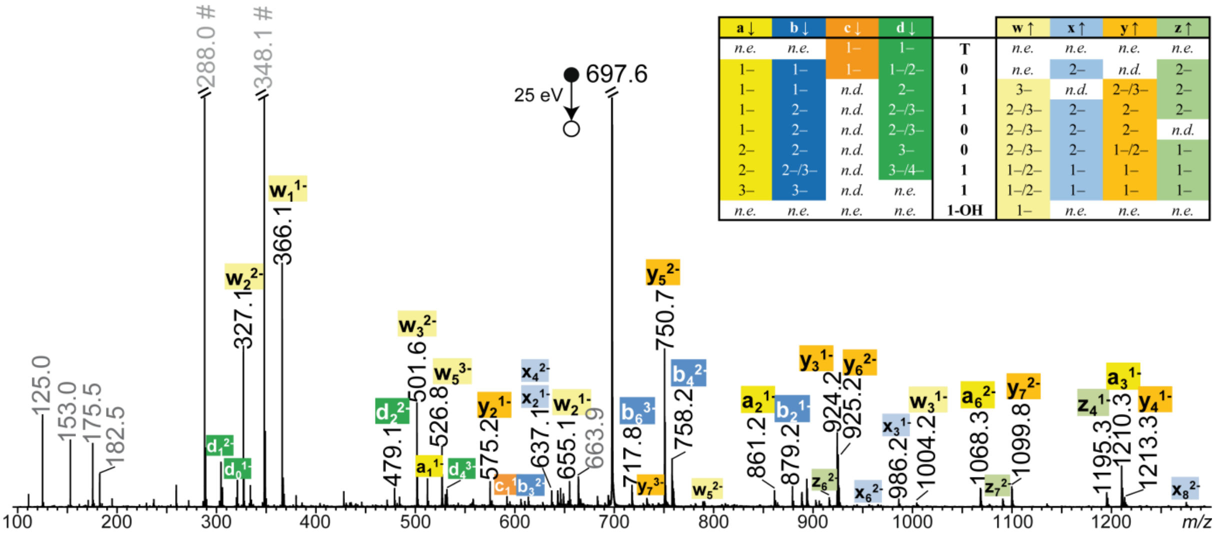

a↓	b↓	c↓	d↓		w↑	x↑	y↑	z↑
n.e.	*n.e.*	1–	1–	**T**	*n.e.*	*n.e.*	*n.e.*	*n.e.*
1–	1–	1–	1–/2–	**0**	*n.e.*	2–	*n.d.*	2–
1–	1–	*n.d.*	2–	**1**	3–	*n.d.*	2–/3–	2–
1–	2–	*n.d.*	2–/3–	**1**	2–/3–	2–	2–	2–
1–	2–	*n.d.*	2–/3–	**0**	2–/3–	2–	2–	*n.d.*
2–	2–	*n.d.*	3–	**0**	2–/3–	2–	1–/2–	1–
2–	2–/3–	*n.d.*	3–/4–	**1**	1–/2–	1–	1–	1–
3–	3–	*n.d.*	*n.e.*	**1**	1–/2–	1–	1–	1–
n.e.	*n.e.*	*n.e.*	*n.e.*	**1-OH**	1–	*n.e.*	*n.e.*	*n.e.*

Supplementary Figure 24. MS/MS spectrum recorded for copolymer **P5** before irradiation, using a 25 eV collision energy (laboratory frame) to activate the quadruply deprotonated precursor at *m/z* 697.6. Peaks annotated in grey correspond to secondary fragments, including the deprotonated units, $[0 - H]^-$ at *m/z* 288.0 and $[1 - H]^-$ at *m/z* 348.1, both designated by #. Inset Table: Sequence coverage of **P5** (*n.d.*: not detected; *n.e.*: not expected). After photo-exposure, the resulting polymer **P5’** exhibits the same MS/MS spectrum as **P1’** in Supplementary Figure 16.

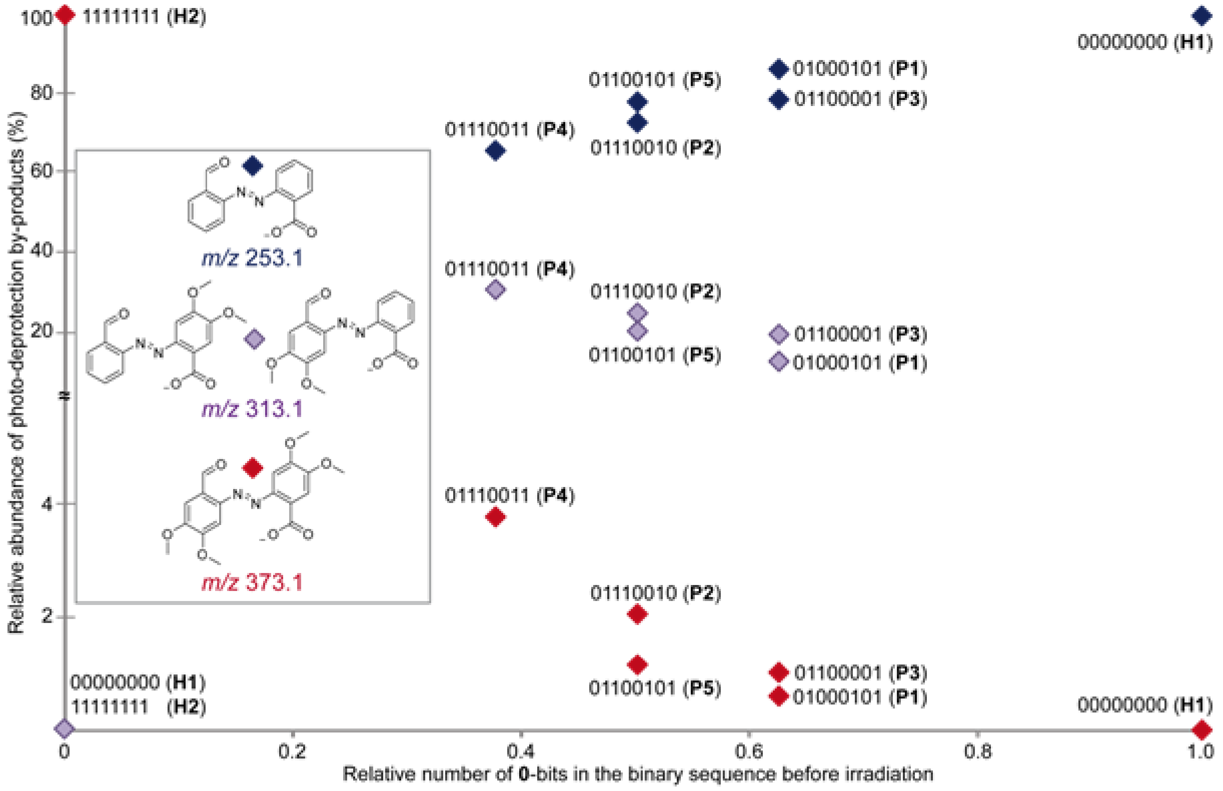

Supplementary Figure 25. Relative abundance of photolysis by-products (structure shown in inset) as a function of 0/1 comonomer composition of the polymer before photo-irradiation. Each data point is annotated by the sequence of the corresponding polymer before irradiation. The relative amount of the homodimer detected at *m/z* 253.1 increases with the fraction of 0-bits in the original sequence. Similarly, the relative abundance of the homodimer detected at *m/z* 373.1 increase with the fraction of 1-bits (i.e., decreasing fraction of 0-bits) in the original sequence. Consistent with the decreasing amount of photocleaved veratryl units in the irradiated solution, the relative intensity of the *m/z* 313.1 signal, assigned to a heterodimer, decreased as the fraction of 0-bits increased in the original sequence.

a.

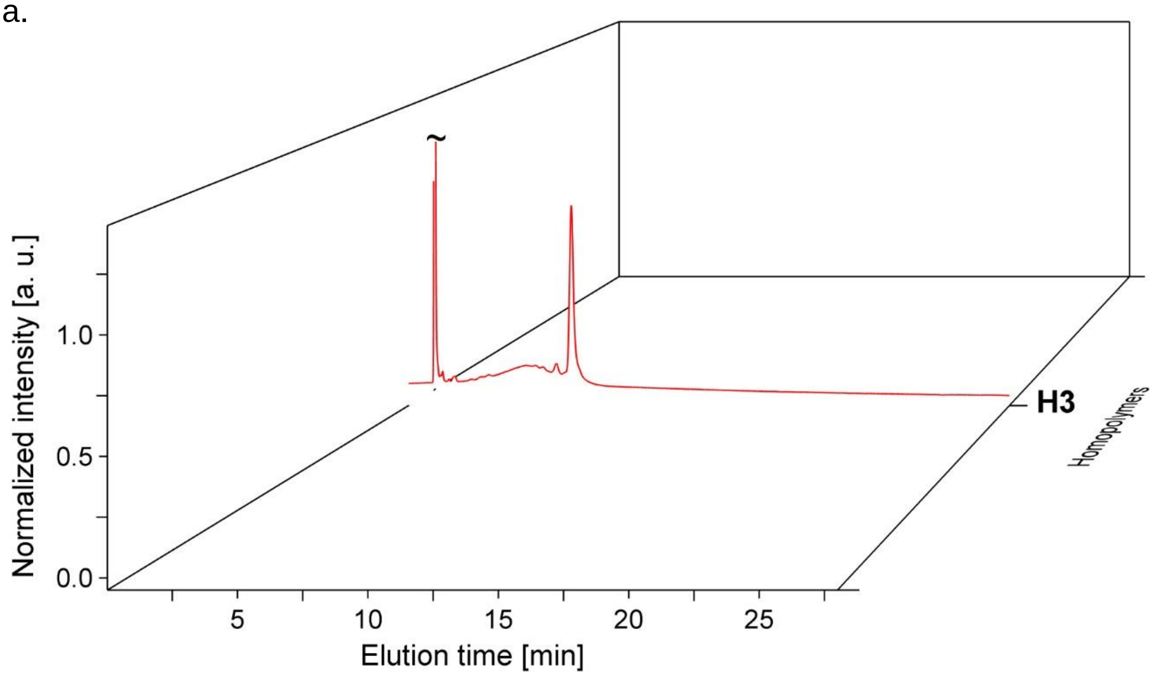

b.

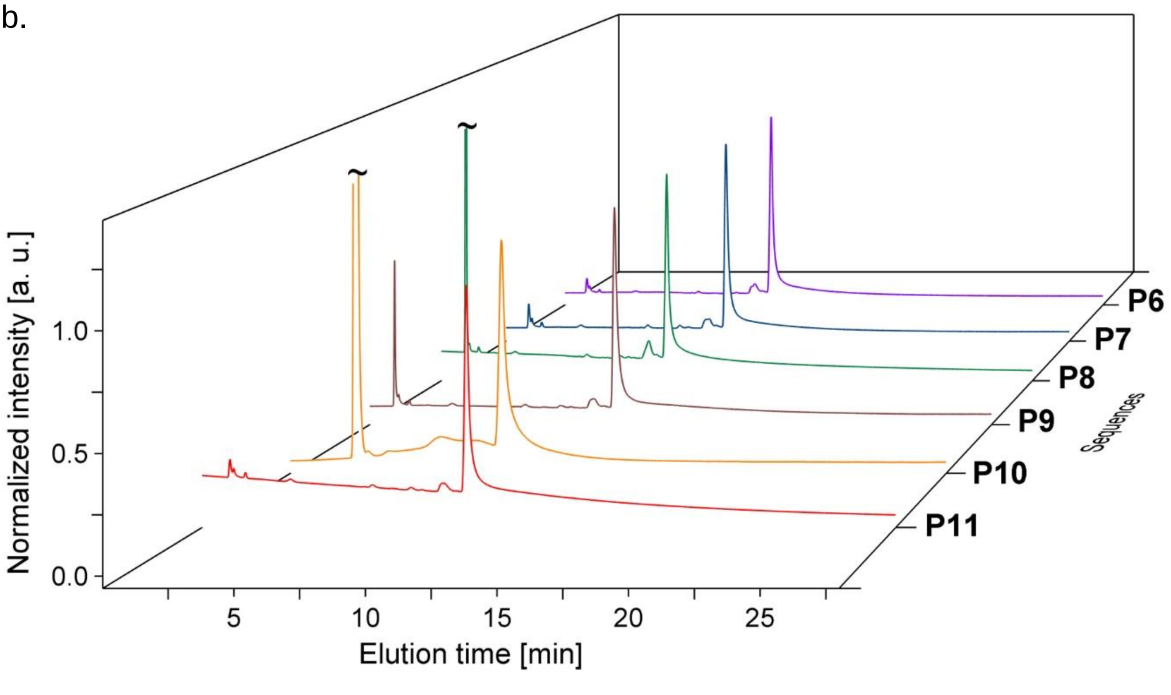

Supplementary Figure 26. HPLC traces measured for (**a**) homopolymers **H3** and (**b**) copolymers **P6-P11**. The chromatograms are recorded at λ =260 nm. Experimental conditions: phase A: 10 % MeCN 20 % 2M NH_3 in water, phase B: 2.5 M NaCl in water; gradient: 0-3 min 5 % B, 3-23 min 5 % B-30 % B, 23-28 min 30 %B, 28-35 min 30%→5 % B; flow rate: 1 mL·min^{-1}.

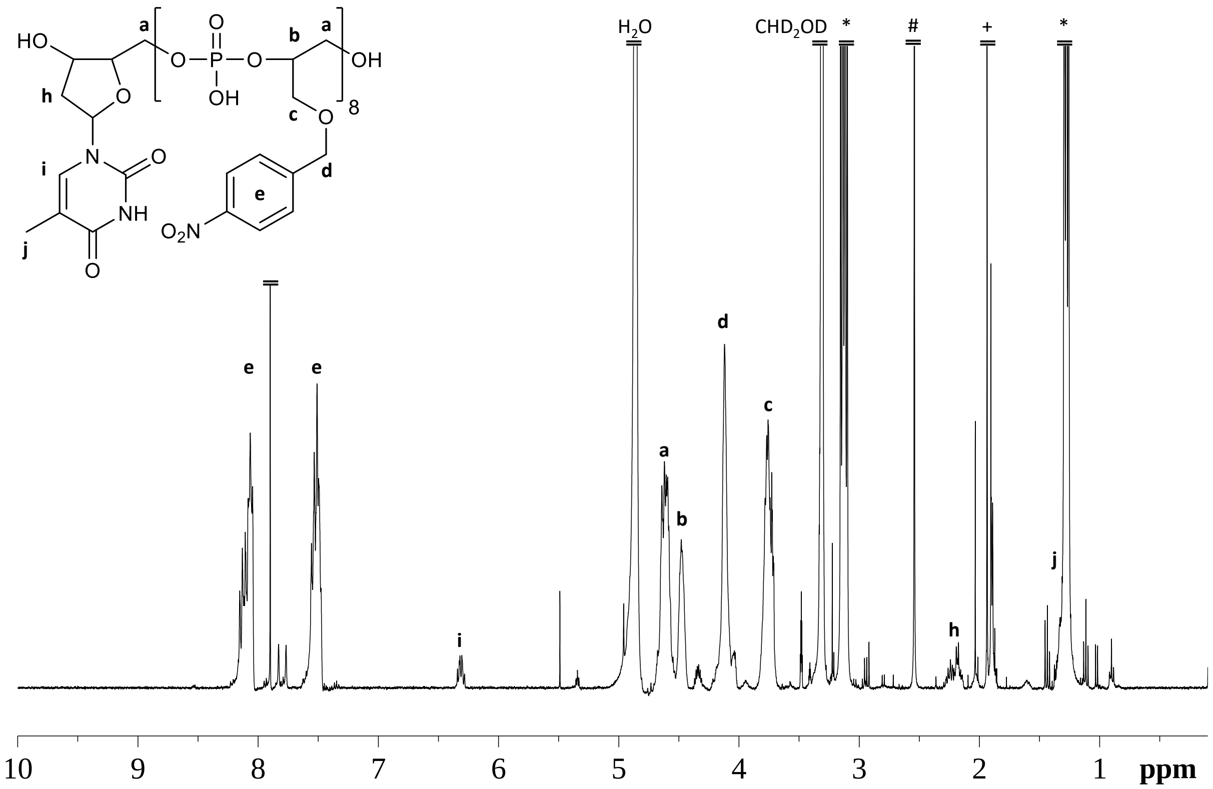

Supplementary Figure 27. ^{1}H NMR spectrum recorded at RT in CD_3OD for homopolymer **H3**. The star symbols indicate signals of triethylammonium counterions. (#) Methylamine, (+) acetonitrile.

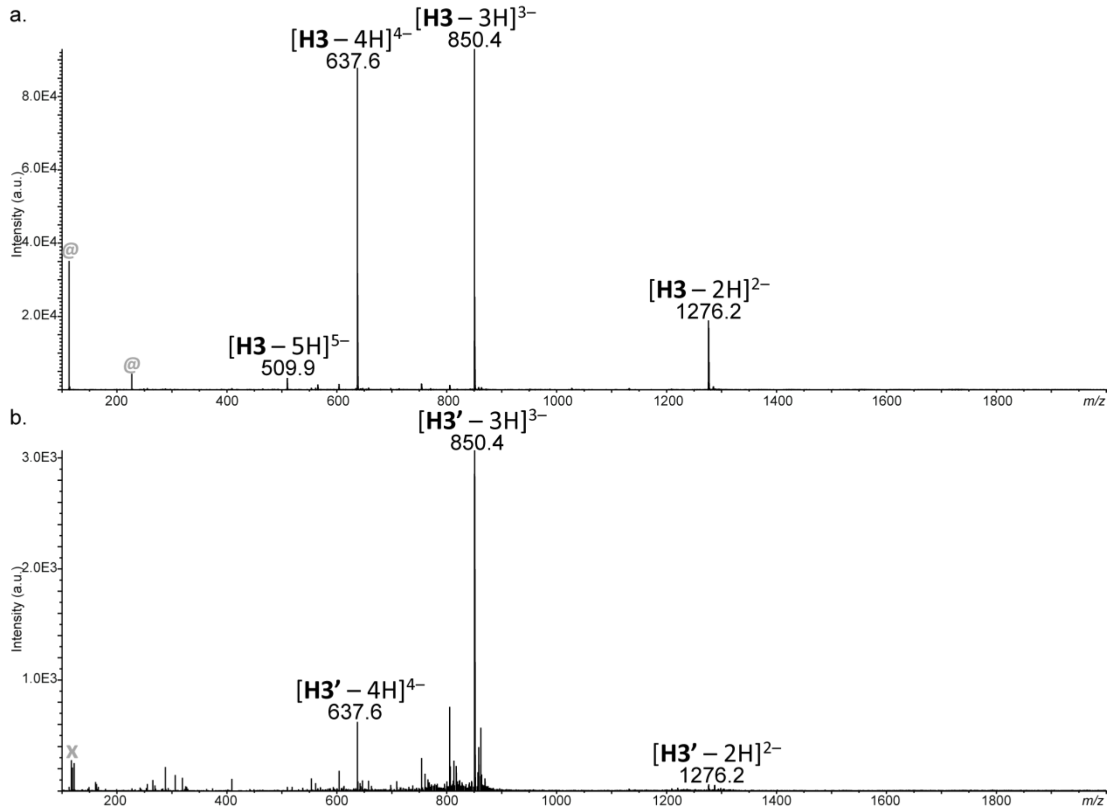

Supplementary Figure 28. ESI HRMS spectra recorded for (**a**) homopolymer **H3** before irradiation and (**b**) the resulting polymer **H3’** after photo-exposure. Grey symbols designate clusters of trifluoroacetic acid (@) and clusters of trichloroacetic acid (x).

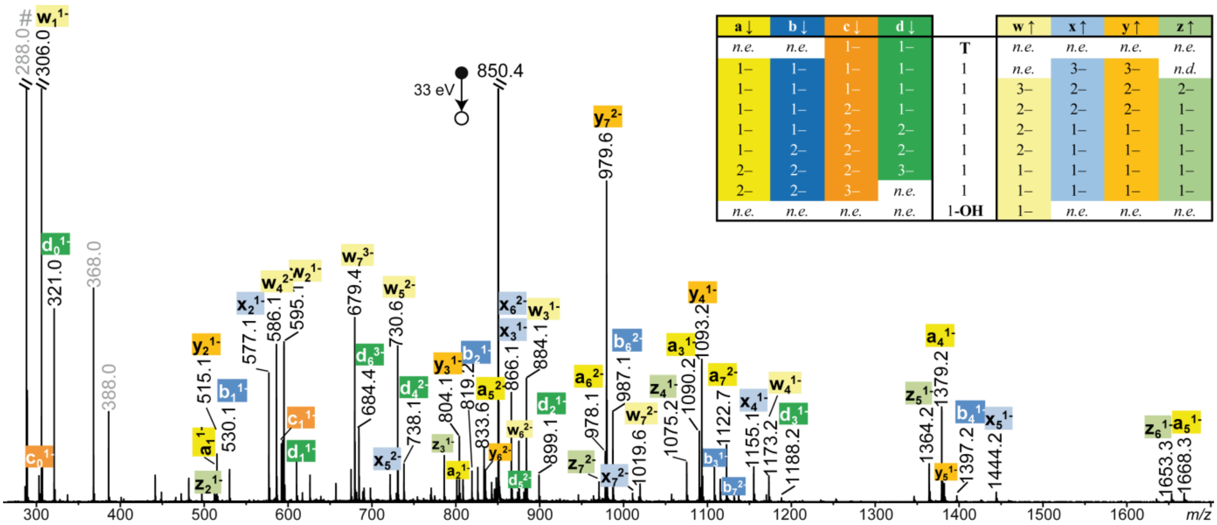

a↓	b↓	c↓	d↓		w↑	x↑	y↑	z↑
n.e.	*n.e.*	1–	1–	**T**	*n.e.*	*n.e.*	*n.e.*	*n.e.*
1–	1–	1–	1–	1	*n.e.*	3–	3–	*n.d.*
1–	1–	1–	1–	1	3–	2–	2–	2–
1–	1–	2–	1–	1	2–	2–	2–	1–
1–	1–	2–	2–	1	2–	1–	1–	1–
1–	2–	2–	2–	1	2–	1–	1–	1–
2–	2–	2–	3–	1	1–	1–	1–	1–
2–	2–	3–	*n.e.*	1	1–	1–	1–	1–
n.e.	*n.e.*	*n.e.*	*n.e.*	**1-OH**	1–	*n.e.*	*n.e.*	*n.e.*

Supplementary Figure 29. MS/MS spectrum recorded for homopolymer **H3** before photo-exposure, using a 33 eV collision energy (laboratory frame) to activate the triply deprotonated precursor at *m/z* 850.4. Peaks annotated in grey correspond to secondary fragments, including the deprotonated unit (*m/z* 288.0) designated by #. The inset table shows that most expected members of the eight fragment series are detected and evidence the monotonic sequence of **H3**. *n.d.*: not detected. *n.e.*: not expected.

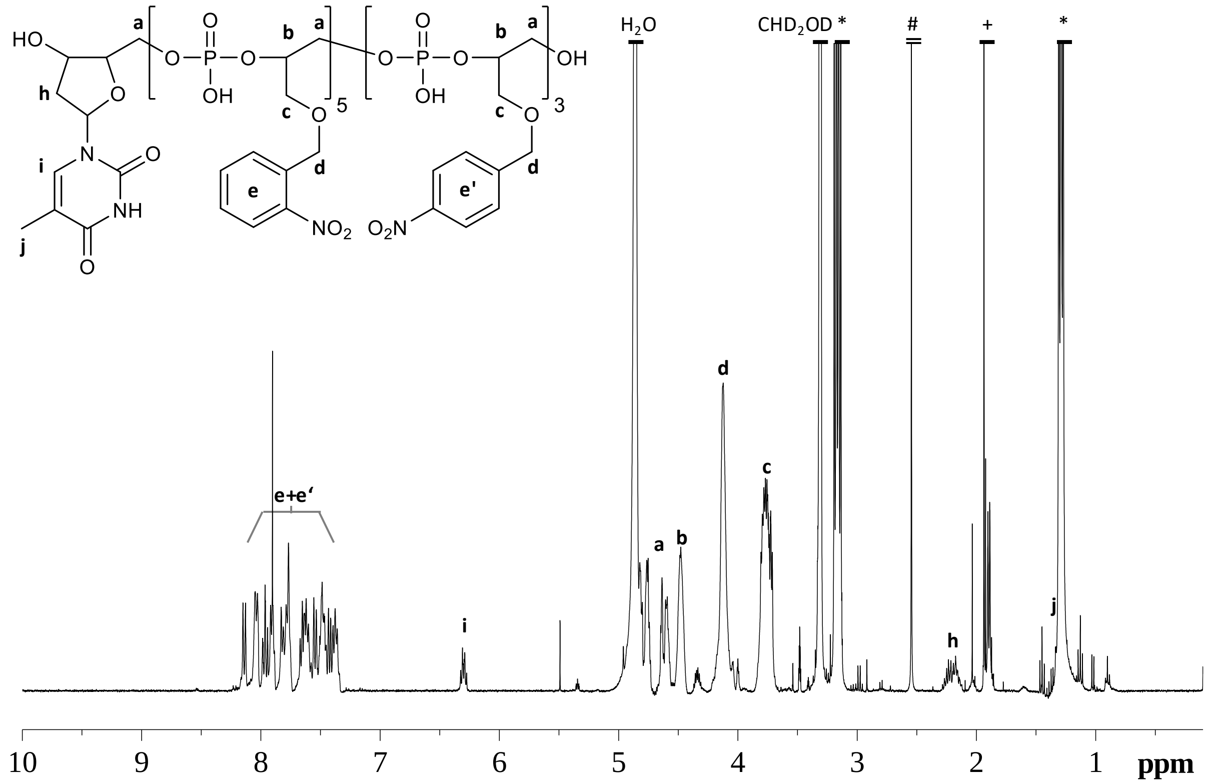

Supplementary Figure 30. ^{1}H NMR spectrum recorded at RT in CD_3OD for copolymer **P6**. The star symbols indicate signals of triethylammonium counterions. (#) Methylamine, (+) acetonitrile.

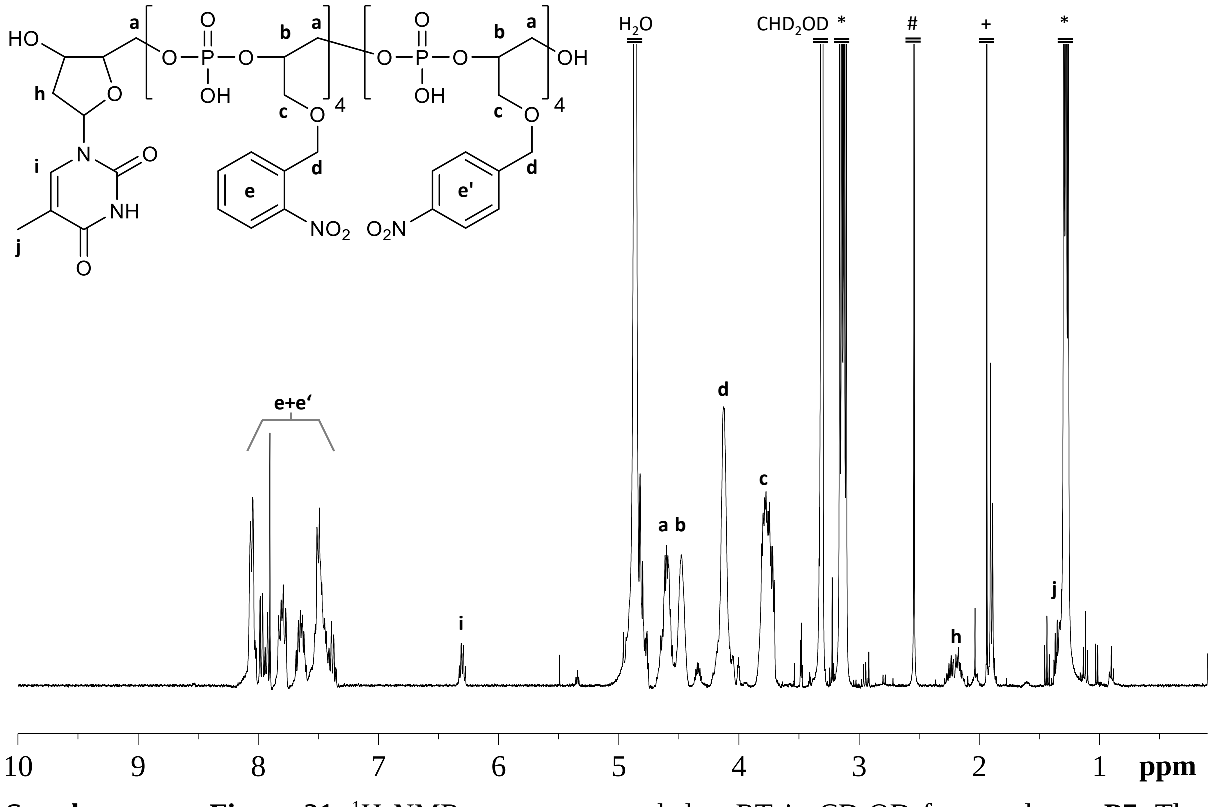

Supplementary Figure 31. ^{1}H NMR spectrum recorded at RT in CD_3OD for copolymer **P7**. The star symbols indicate signals of triethylammonium counterions. (#) Methylamine, (+) acetonitrile.

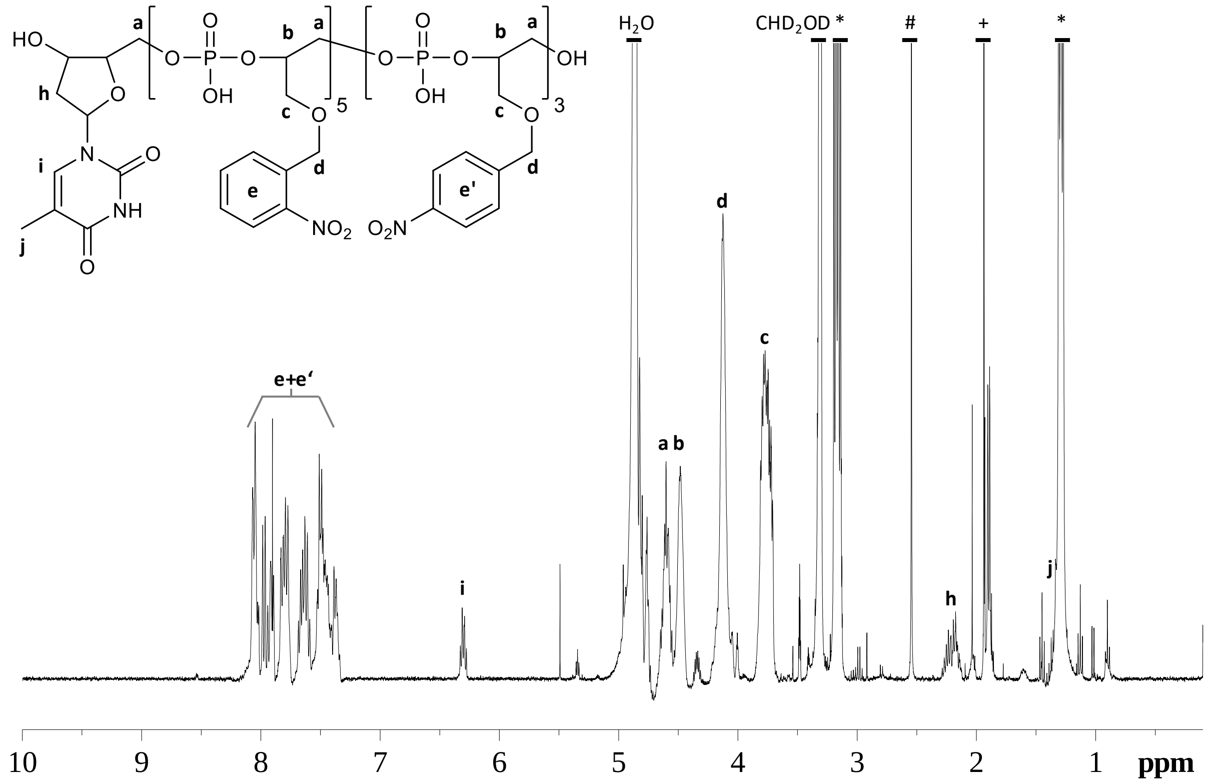

Supplementary Figure 32. ^{1}H NMR spectrum recorded at RT in CD_3OD for copolymer **P8**. The star symbols indicate signals of triethylammonium counterions. (#) Methylamine, (+) acetonitrile.

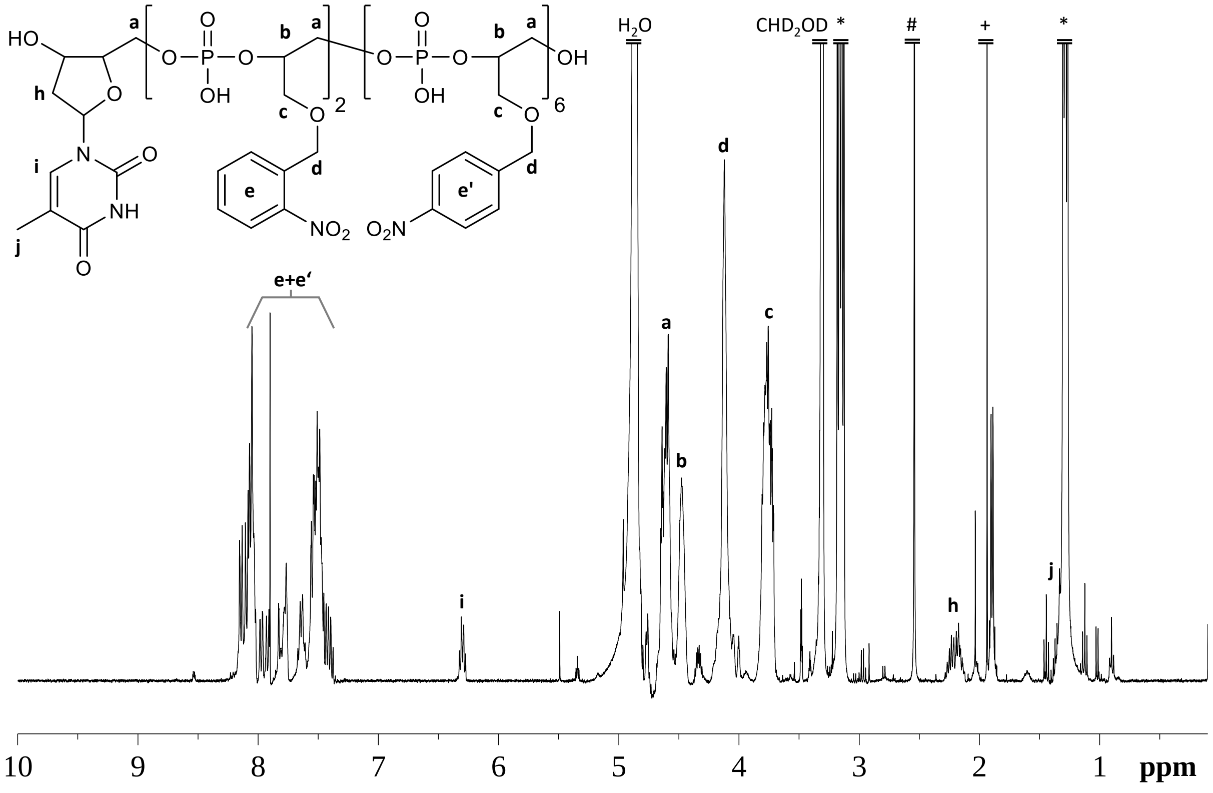

Supplementary Figure 33. ^{1}H NMR spectrum recorded at RT in CD_3OD for copolymer **P9**. The star symbols indicate signals of triethylammonium counterions. (#) Methylamine, (+) acetonitrile.

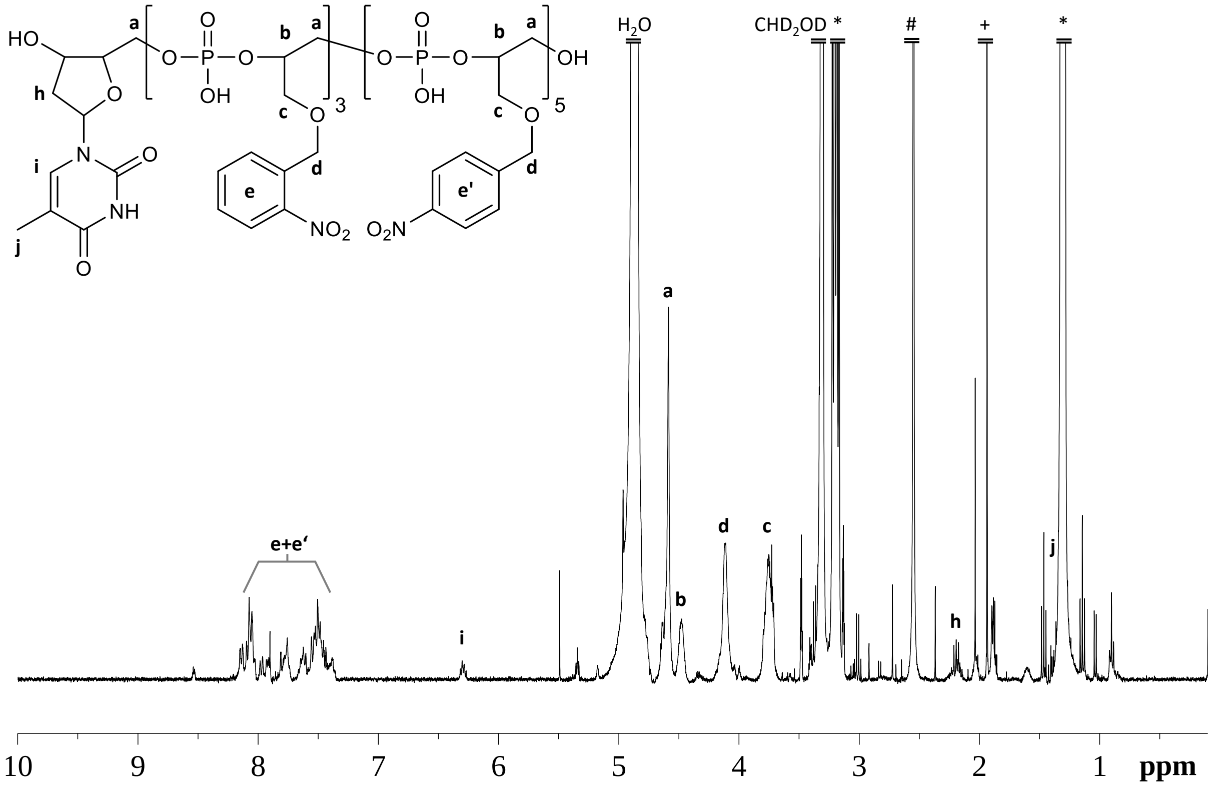

Supplementary Figure 34. ^{1}H NMR spectrum recorded at RT CD_3OD for copolymer **P10**. The star symbols indicate signals of triethylammonium counterions. (#) Methylamine, (+) acetonitrile.

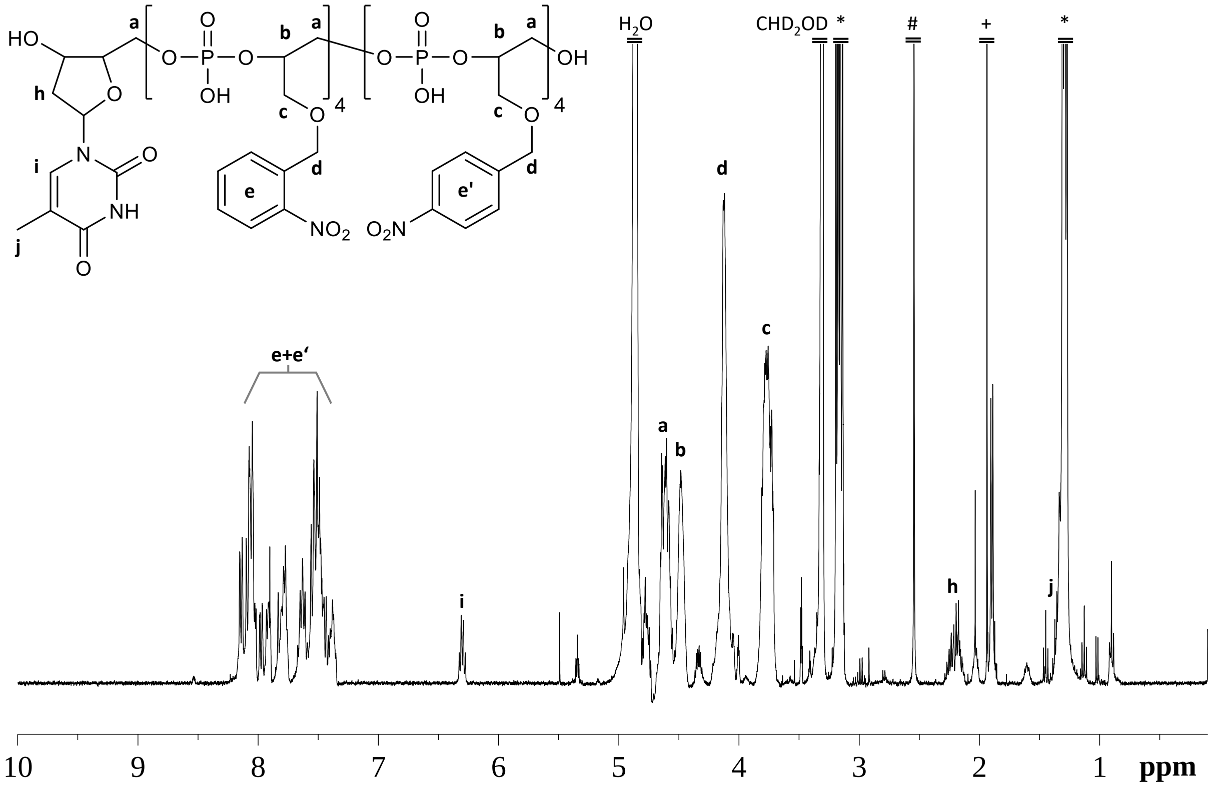

Supplementary Figure 35. ^{1}H NMR spectrum recorded at RT CD_3OD for copolymer **P11**. The star symbols indicate signals of triethylammonium counterions. (#) Methylamine, (+) acetonitrile.

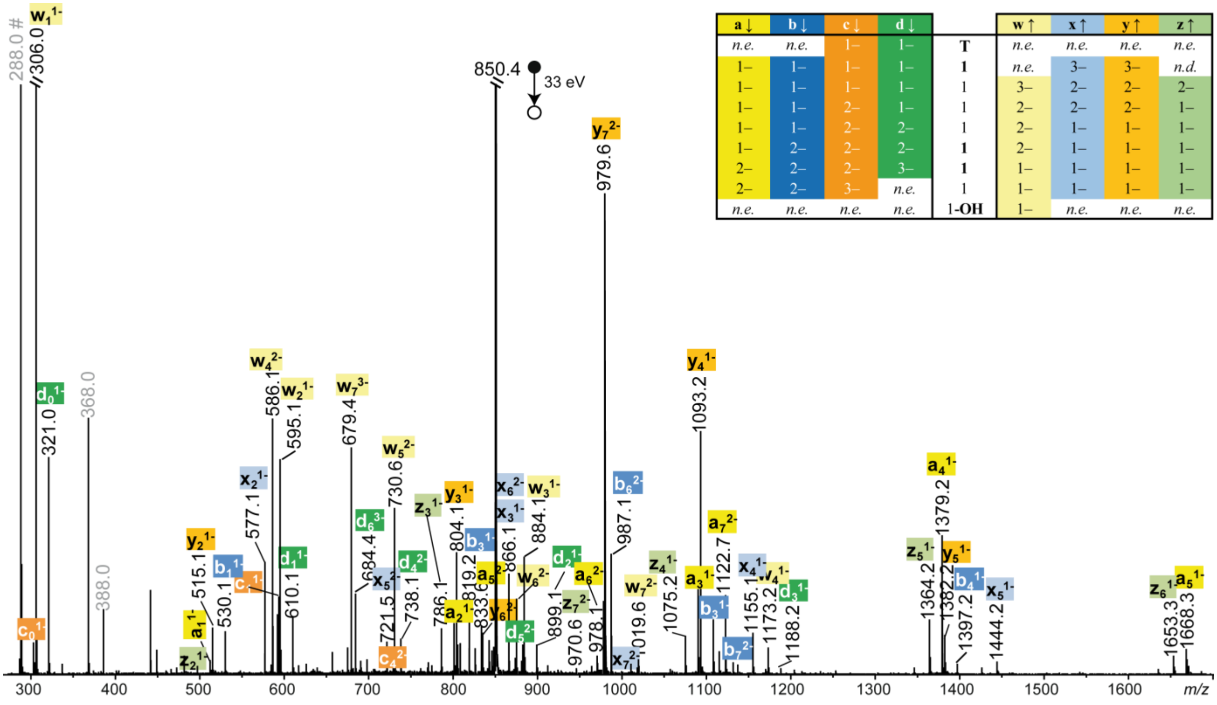

a↓	b↓	c↓	d↓		w↑	x↑	y↑	z↑
n.e.	*n.e.*	1–	1–	**T**	*n.e.*	*n.e.*	*n.e.*	*n.e.*
1–	1–	1–	1–	**1**	*n.e.*	3–	3–	*n.d.*
1–	1–	1–	1–	1	3–	2–	2–	2–
1–	1–	2–	1–	1	2–	2–	2–	1–
1–	1–	2–	2–	1	2–	1–	1–	1–
1–	2–	2–	2–	**1**	2–	1–	1–	1–
2–	2–	2–	3–	**1**	1–	1–	1–	1–
2–	2–	3–	*n.e.*	1	1–	1–	1–	1–
n.e.	*n.e.*	*n.e.*	*n.e.*	1-**OH**	1–	*n.e.*	*n.e.*	*n.e.*

Supplementary Figure 36. MS/MS spectrum recorded for copolymer **P10** before irradiation, using a 33 eV collision energy (laboratory frame) to activate the triply deprotonated precursor at *m/z* 850.4. Peaks annotated in grey correspond to secondary fragments, including deprotonated units (both at *m/z* 288.0) designated by #. The inset table shows that all (but one) expected members of the eight fragment series are detected and evidence the monotonic sequence of **P10** (*n.d.*: not detected; *n.e.*: not expected).

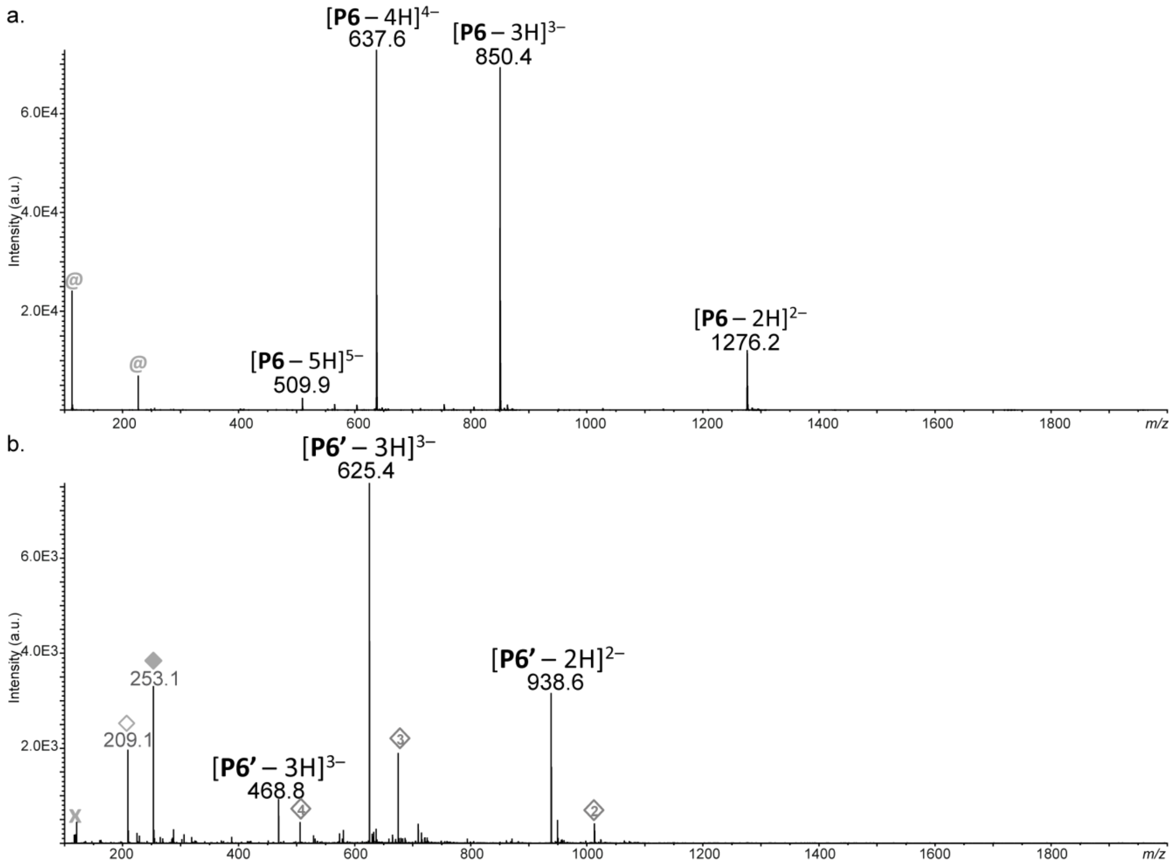

Supplementary Figure 37. ESI HRMS spectra recorded for (**a**) copolymer **P6** before irradiation and (**b**) the resulting polymer **P6'** after photo-exposure. Grey symbols designate clusters of trifluoroacetic acid (@), clusters of trichloroacetic acid (x) or photo-deprotection by-products (diamonds).

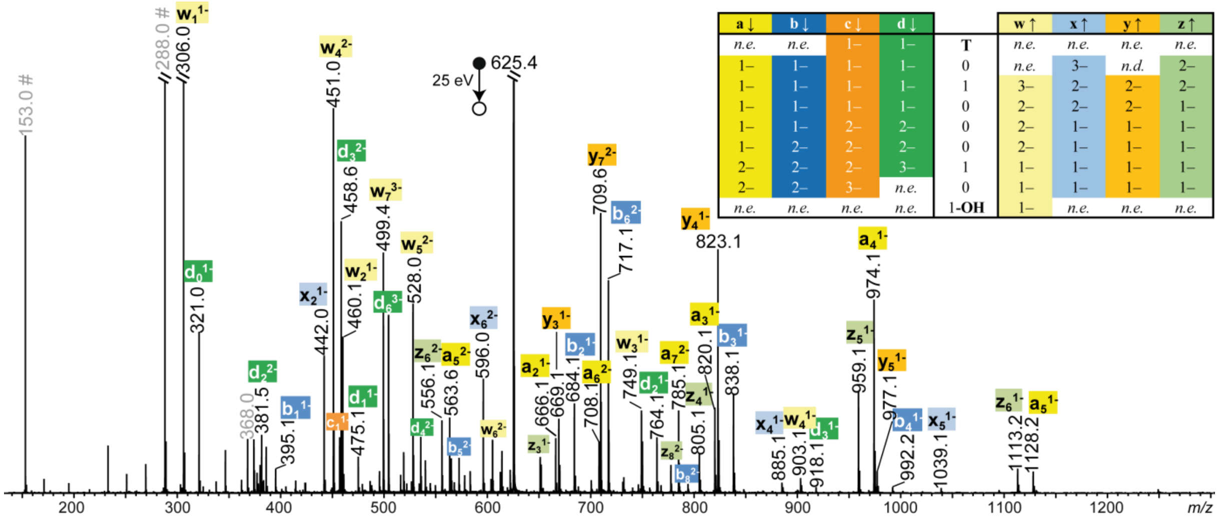

a↓	b↓	c↓	d↓		w↑	x↑	y↑	z↑
n.e.	*n.e.*	1–	1–	**T**	*n.e.*	*n.e.*	*n.e.*	*n.e.*
1–	1–	1–	1–	0	*n.e.*	3–	*n.d.*	2–
1–	1–	1–	1–	1	3–	2–	2–	2–
1–	1–	1–	1–	0	2–	2–	2–	1–
1–	1–	2–	2–	0	2–	1–	1–	1–
1–	2–	2–	2–	0	2–	1–	1–	1–
2–	2–	2–	3–	1	1–	1–	1–	1–
2–	2–	3–	*n.e.*	0	1–	1–	1–	1–
n.e.	*n.e.*	*n.e.*	*n.e.*	**1-OH**	1–	*n.e.*	*n.e.*	*n.e.*

Supplementary Figure 38. MS/MS spectrum recorded for copolymer **P6'** obtained after irradiation of **P6**, using a 25 eV collision energy (laboratory frame) to activate the triply deprotonated precursor at *m/z* 625.4. Peaks annotated in grey correspond to secondary fragments, including the deprotonated units, $[0 - H]^-$ at *m/z* 153.0 and $[1 - H]^-$ at *m/z* 288.0, both designated by #. Inset Table: Sequence coverage of **P6'** (*n.d.*: not detected; *n.e.*: not expected). Before photo-exposure, copolymer **P6** exhibits the same MS/MS spectrum as **P10** in Supplementary Figure 35.

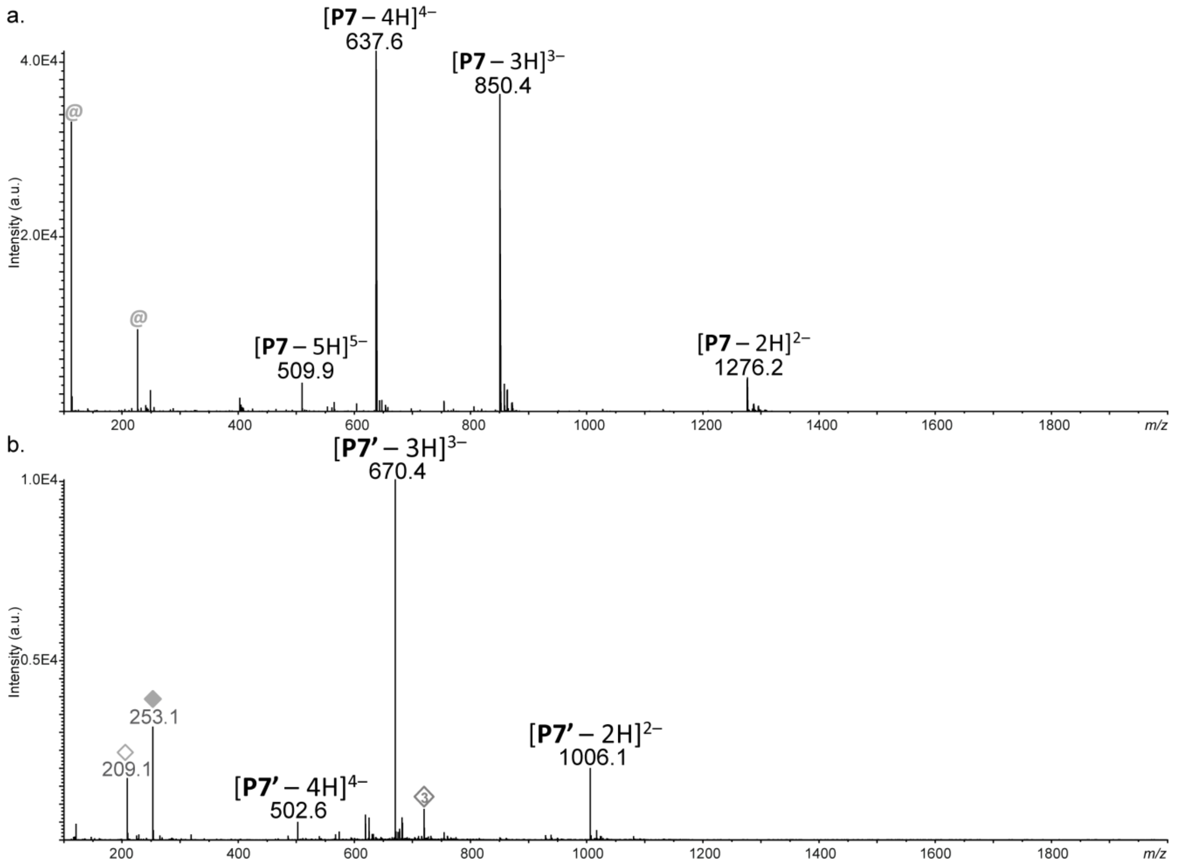

Supplementary Figure 39. ESI HRMS spectra recorded for (**a**) copolymer **P7** before irradiation and (**b**) the resulting polymer **P7’** after photo-exposure. Grey symbols designate clusters of trifluoroacetic acid (@) or photo-deprotection by-products (diamonds).

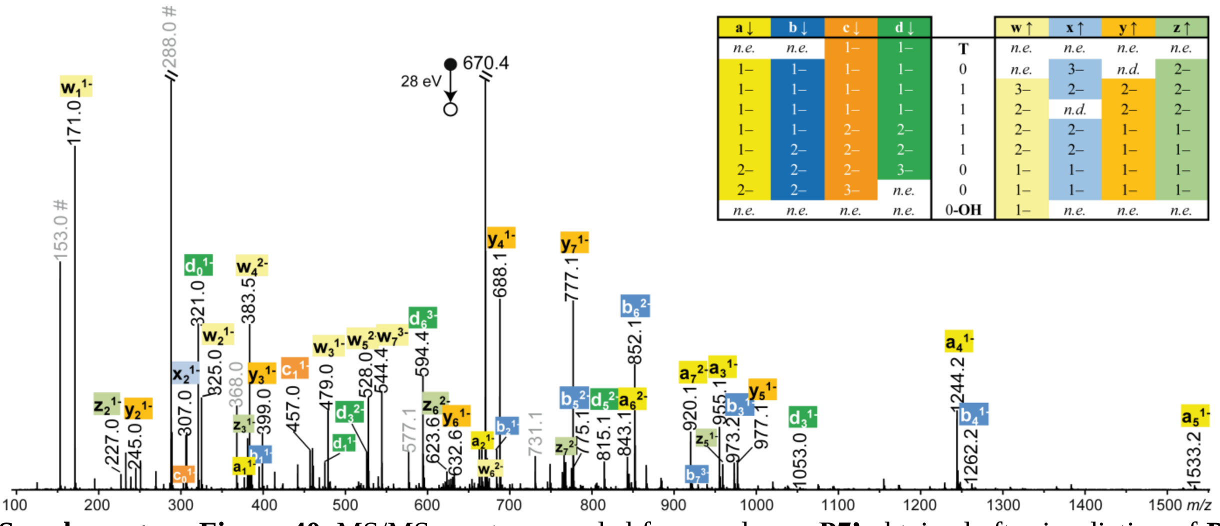

a↓	b↓	c↓	d↓		w↑	x↑	y↑	z↑
n.e.	*n.e.*	1–	1–	**T**	*n.e.*	*n.e.*	*n.e.*	*n.e.*
1–	1–	1–	1–	0	*n.e.*	3–	*n.d.*	2–
1–	1–	1–	1–	1	3–	2–	2–	2–
1–	1–	1–	1–	1	2–	*n.d.*	2–	2–
1–	1–	2–	2–	1	2–	2–	1–	1–
1–	2–	2–	2–	1	2–	2–	1–	1–
2–	2–	2–	3–	0	1–	1–	1–	1–
2–	2–	3–	*n.e.*	0	1–	1–	1–	1–
n.e.	*n.e.*	*n.e.*	*n.e.*	0-**OH**	1–	*n.e.*	*n.e.*	*n.e.*

Supplementary Figure 40. MS/MS spectra recorded for copolymer **P7’** obtained after irradiation of **P7**, using a 28 eV collision energy (laboratory frame) to activate the triply deprotonated precursor at *m/z* 670.4. Peaks annotated in grey correspond to secondary fragments, including the deprotonated units, $[0 - H]^-$ at *m/z* 153.0 and $[1 - H]^-$ at *m/z* 288.0, both designated by #. Inset Table: Sequence coverage of **P7’** (*n.d.*: not detected; *n.e.*: not expected). Before photo-exposure, copolymer **P7** exhibits the same MS/MS spectrum as **P10** in Supplementary Figure 35.

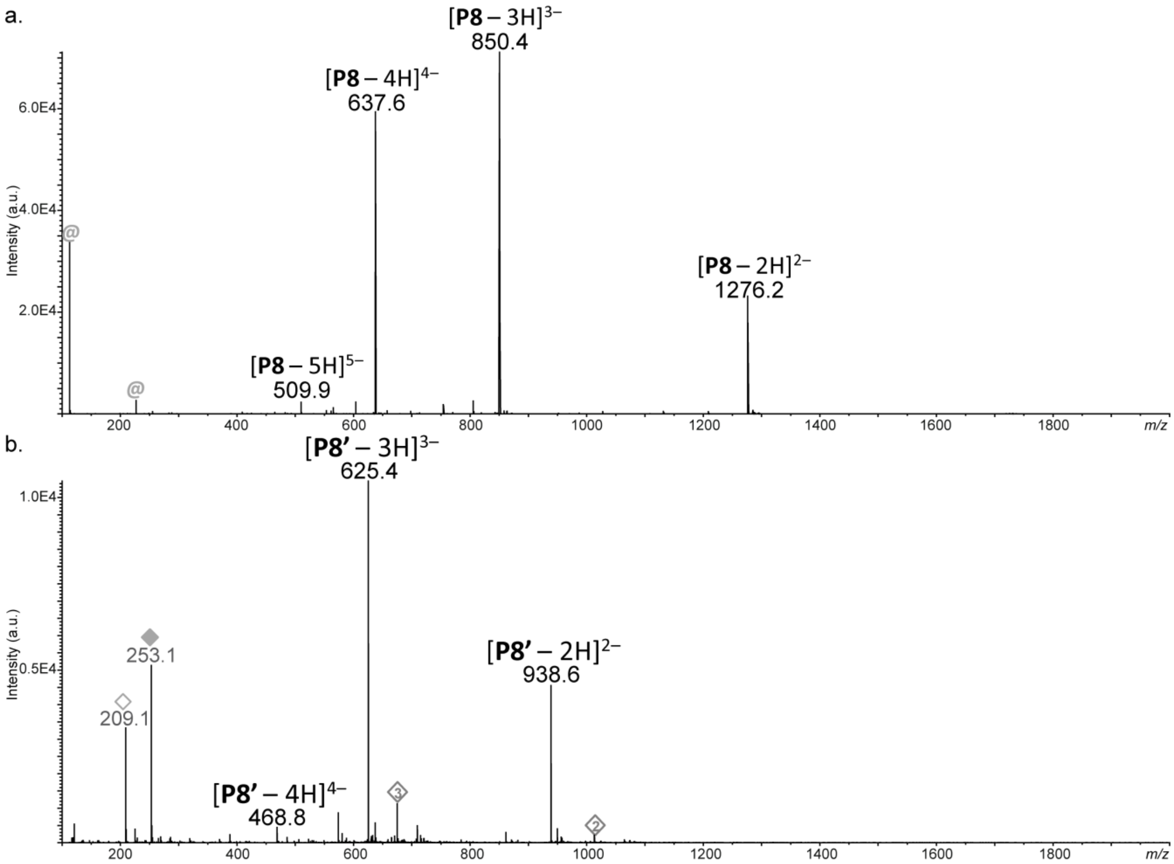

Supplementary Figure 41. ESI HRMS spectra recorded for (**a**) copolymer **P8** before irradiation and (**b**) the resulting polymer **P8'** after photo-exposure. Grey symbols designate clusters of trifluoroacetic acid (@) or photo-deprotection by-products (diamonds).

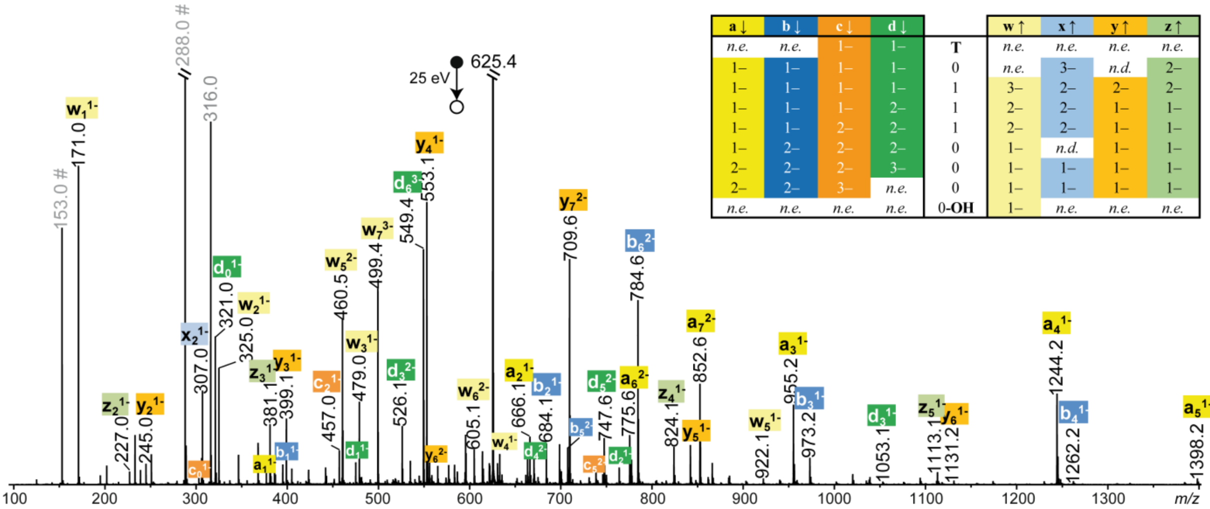

a↓	b↓	c↓	d↓		w↑	x↑	y↑	z↑
n.e.	*n.e.*	1–	1–	**T**	*n.e.*	*n.e.*	*n.e.*	*n.e.*
1–	1–	1–	1–	0	*n.e.*	3–	*n.d.*	2–
1–	1–	1–	1–	1	3–	2–	2–	2–
1–	1–	1–	2–	1	2–	2–	1–	1–
1–	1–	2–	2–	1	2–	2–	1–	1–
1–	2–	2–	2–	0	1–	*n.d.*	1–	1–
2–	2–	2–	3–	0	1–	1–	1–	1–
2–	2–	3–	*n.e.*	0	1–	1–	1–	1–
n.e.	*n.e.*	*n.e.*	*n.e.*	0-**OH**	1–	*n.e.*	*n.e.*	*n.e.*

Supplementary Figure 42. MS/MS spectra recorded for copolymer **P8'** obtained after irradiation of **P8**, using a 25 eV collision energy (laboratory frame) to activate the triply deprotonated precursor at *m/z* 625.4. Peaks annotated in grey correspond to secondary fragments, including the deprotonated units, $[0 - H]^-$ at *m/z* 153.0 and $[1 - H]^-$ at *m/z* 288.0, both designated by #. Inset Table: Sequence coverage of **P8'** (*n.d.*: not detected; *n.e.*: not expected). Before photo-exposure, copolymer **P8** exhibits the same MS/MS spectrum as **P10** in Supplementary Figure 35.

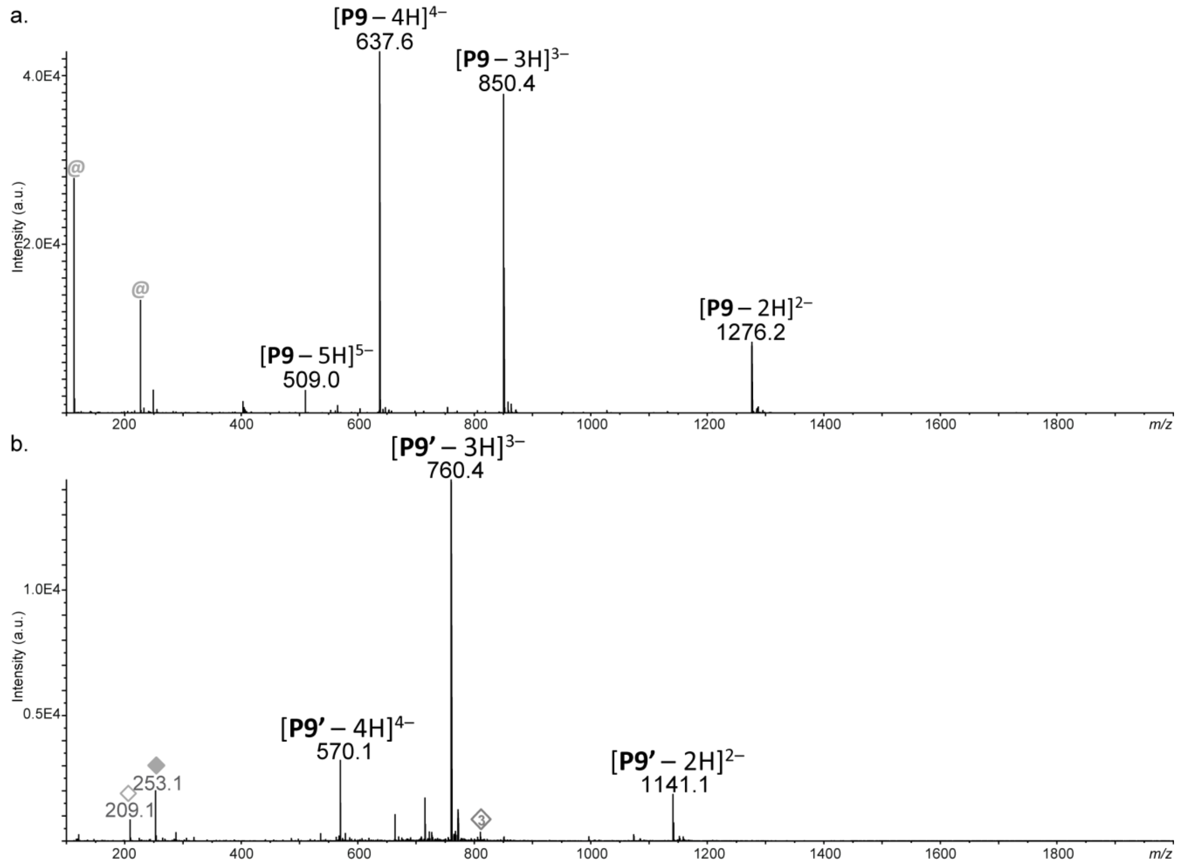

Supplementary Figure 43. ESI HRMS spectra recorded for (**a**) copolymer **P9** before irradiation and (**b**) the resulting polymer **P9'** after photo-exposure. Grey symbols designate clusters of trifluoroacetic acid (@) or photo-deprotection by-products (diamonds).

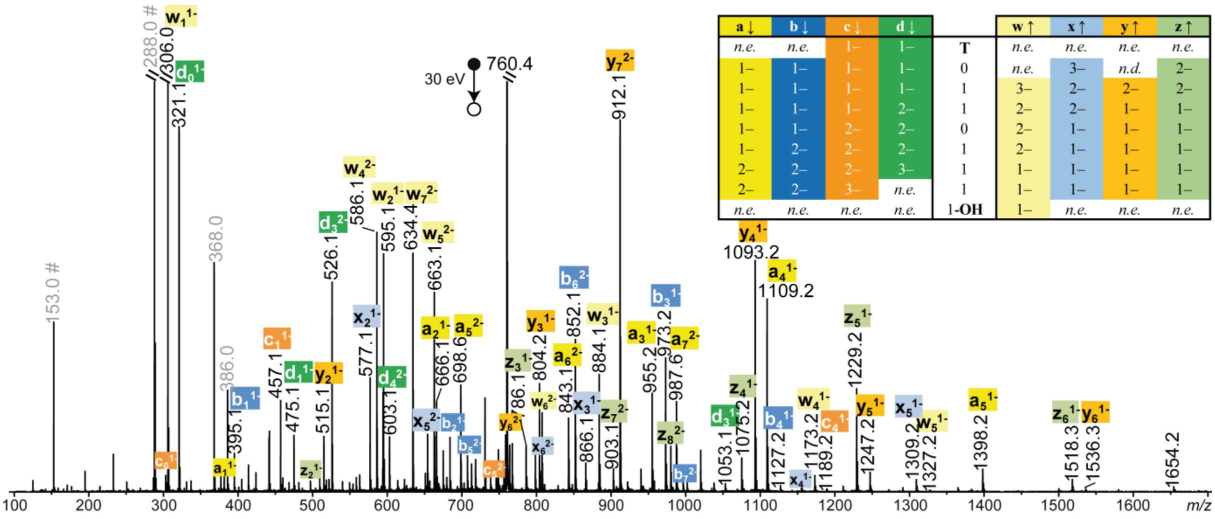

a ↓	b ↓	c ↓	d ↓		w ↑	x ↑	y ↑	z ↑
n.e.	*n.e.*	1–	1–	**T**	*n.e.*	*n.e.*	*n.e.*	*n.e.*
1–	1–	1–	1–	0	*n.e.*	3–	*n.d.*	2–
1–	1–	1–	1–	1	3–	2–	2–	2–
1–	1–	1–	2–	1	2–	2–	1–	1–
1–	1–	2–	2–	0	2–	1–	1–	1–
1–	2–	2–	2–	1	2–	1–	1–	1–
2–	2–	2–	3–	1	1–	1–	1–	1–
2–	2–	3–	*n.e.*	1	1–	1–	1–	1–
n.e.	*n.e.*	*n.e.*	*n.e.*	1**-OH**	1–	*n.e.*	*n.e.*	*n.e.*

Supplementary Figure 44. MS/MS spectra recorded for copolymer **P9'** obtained after irradiation of **P9**, using a 30 eV collision energy (laboratory frame) to activate the triply deprotonated precursor at *m/z* 760.4. Peaks annotated in grey correspond to secondary fragments, including the deprotonated units, $[0 – H]^–$ at *m/z* 153.0 and $[1 – H]^–$ at *m/z* 288.0, both designated by #. Inset Table: Sequence coverage of **P9'** (*n.d.*: not detected; *n.e.*: not expected). Before photo-exposure, copolymer **P9** exhibits the same MS/MS spectrum as **P10** in Supplementary Figure 35.

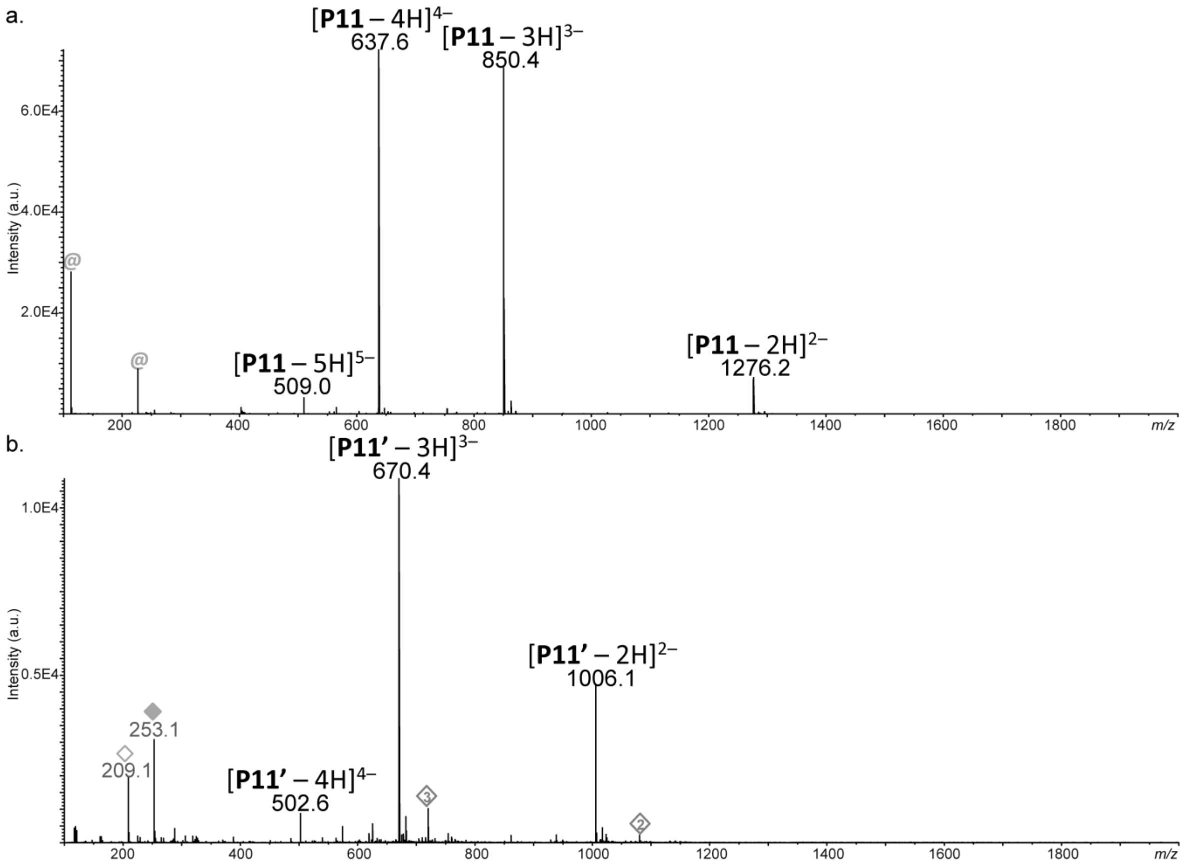

Supplementary Figure 45. ESI HRMS spectra recorded for (**a**) copolymer **P11** before irradiation and (**b**) the resulting polymer **P11’** after photo-exposure. Grey symbols designate clusters of trifluoroacetic acid (@) or photo-deprotection by-products (diamonds).

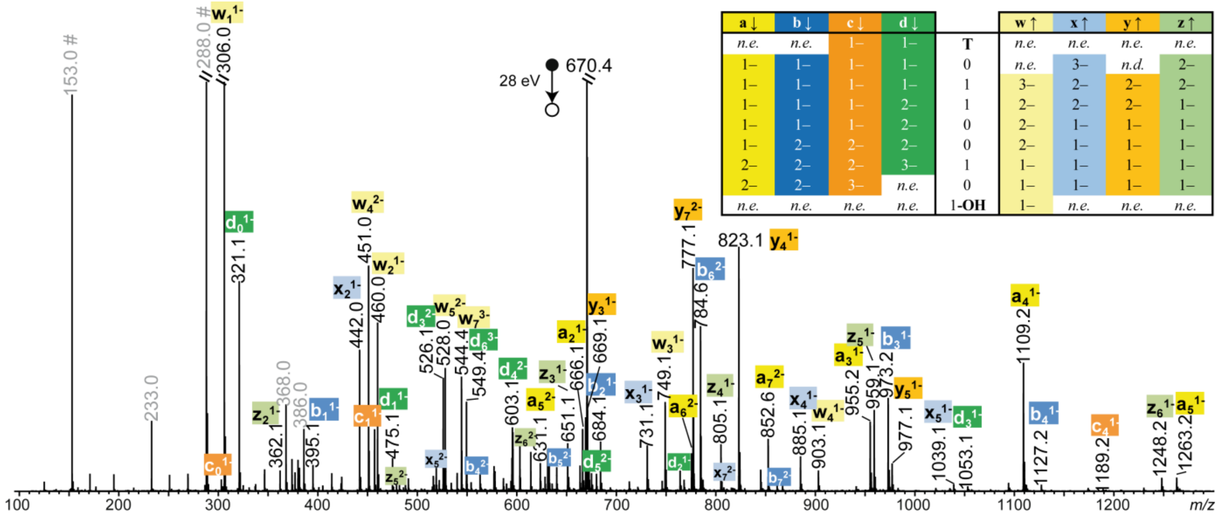

a↓	b↓	c↓	d↓		w↑	x↑	y↑	z↑
n.e.	*n.e.*	1–	1–	**T**	*n.e.*	*n.e.*	*n.e.*	*n.e.*
1–	1–	1–	1–	0	*n.e.*	3–	*n.d.*	2–
1–	1–	1–	1–	1	3–	2–	2–	2–
1–	1–	1–	2–	1	2–	2–	2–	1–
1–	1–	1–	2–	0	2–	1–	1–	1–
1–	2–	2–	2–	0	2–	1–	1–	1–
2–	2–	2–	3–	1	1–	1–	1–	1–
2–	2–	3–	*n.e.*	0	1–	1–	1–	1–
n.e.	*n.e.*	*n.e.*	*n.e.*	**1-OH**	1–	*n.e.*	*n.e.*	*n.e.*

Supplementary Figure 46. MS/MS spectra recorded for copolymer **P11’** obtained after irradiation of **P11**, using a 28 eV collision energy (laboratory frame) to activate the triply deprotonated precursor at *m/z* 670.4. Peaks annotated in grey correspond to secondary fragments, including the deprotonated units, $[0 – H]^–$ at *m/z* 153.0 and $[1 – H]^–$ at *m/z* 288.0, both designated by #. Inset Table: Sequence coverage of **P11’** (*n.d.*: not detected; *n.e.*: not expected). Before photo-exposure, copolymer **P11** exhibits the same MS/MS spectrum as **P10** in Supplementary Figure 35.

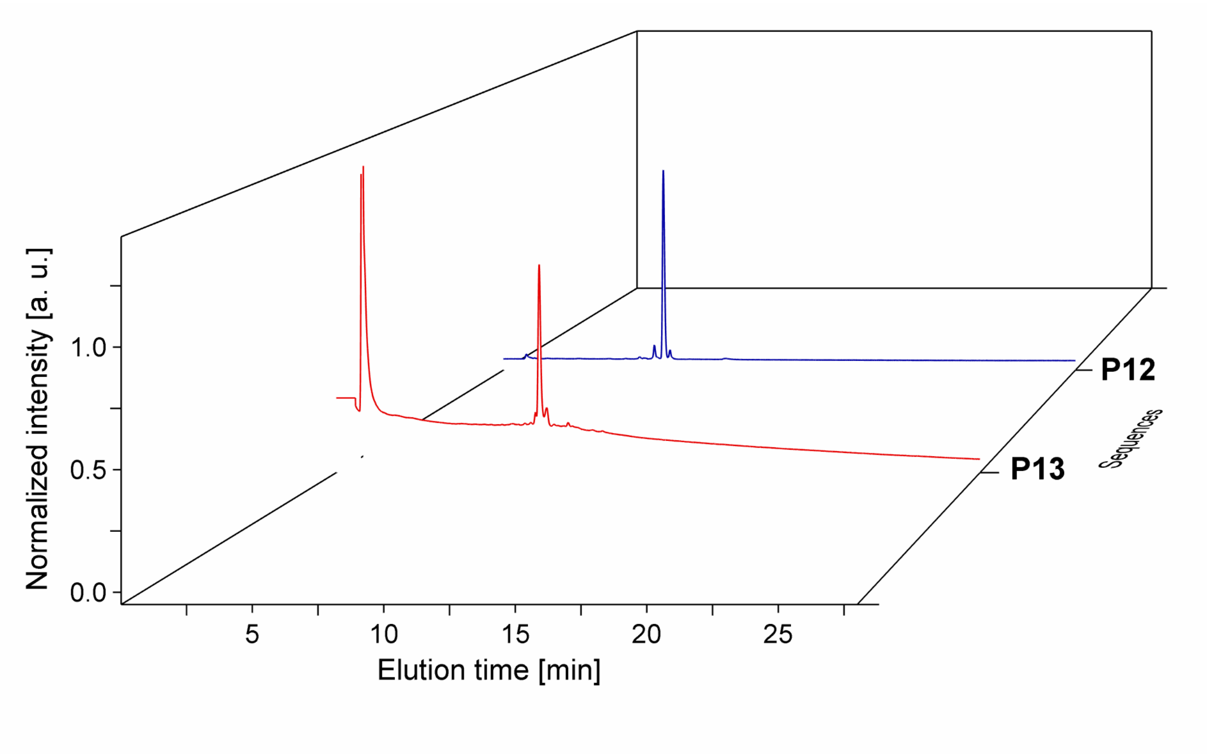

Supplementary Figure 47. HPLC traces measured for copolymers **P12** and **P13**. The chromatogram is recorded at λ=260 nm. Experimental conditions: phase A: 10 % ACN 20 % 2M NH_3 in water, phase B: 2.5 M NaCl in water; gradient: 0-3 min 5 % B, 3-23 min 5 % B-30 % B, 23-28 min 30 %B, 28-35 min 30%→5 % B; flow rate: 1 mL·min^{-1}.

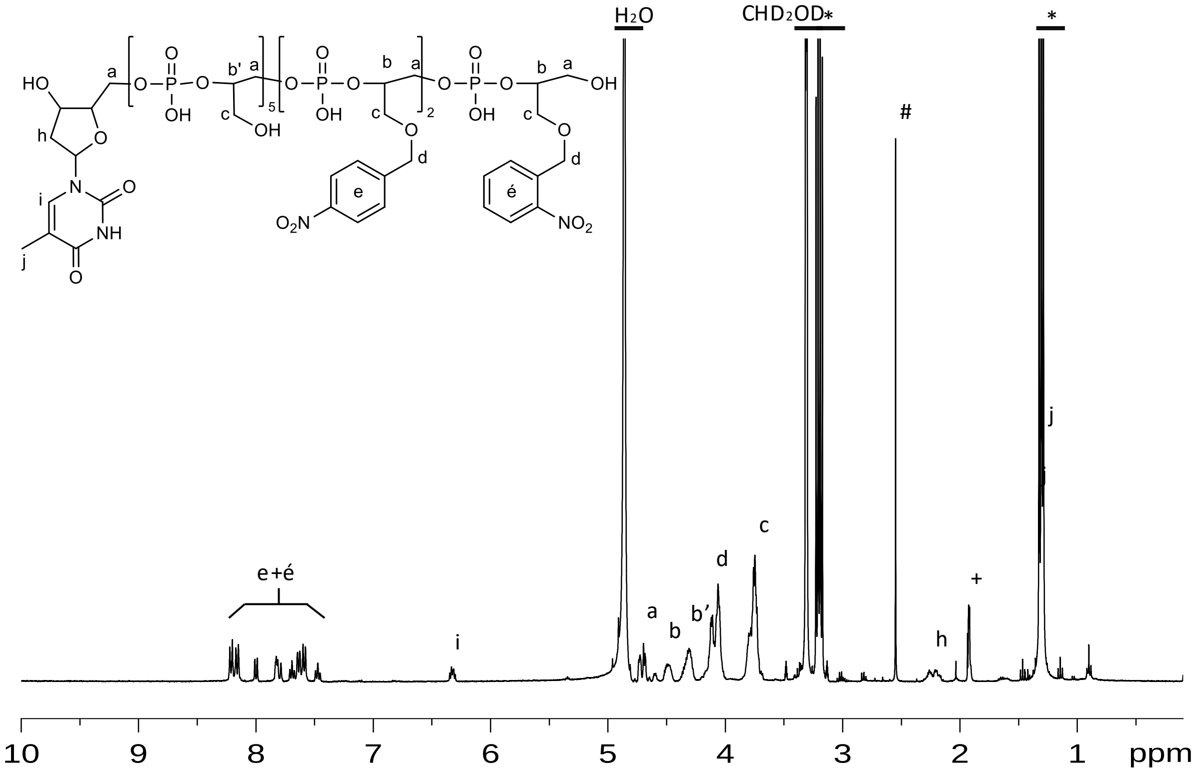

Supplementary Figure 48. ^{1}H NMR spectrum recorded at RT in CD_3OD for copolymer **P12**. The star symbols indicate signals of triethylammonium counterions. (#) Methylamine, (+) acetonitrile.

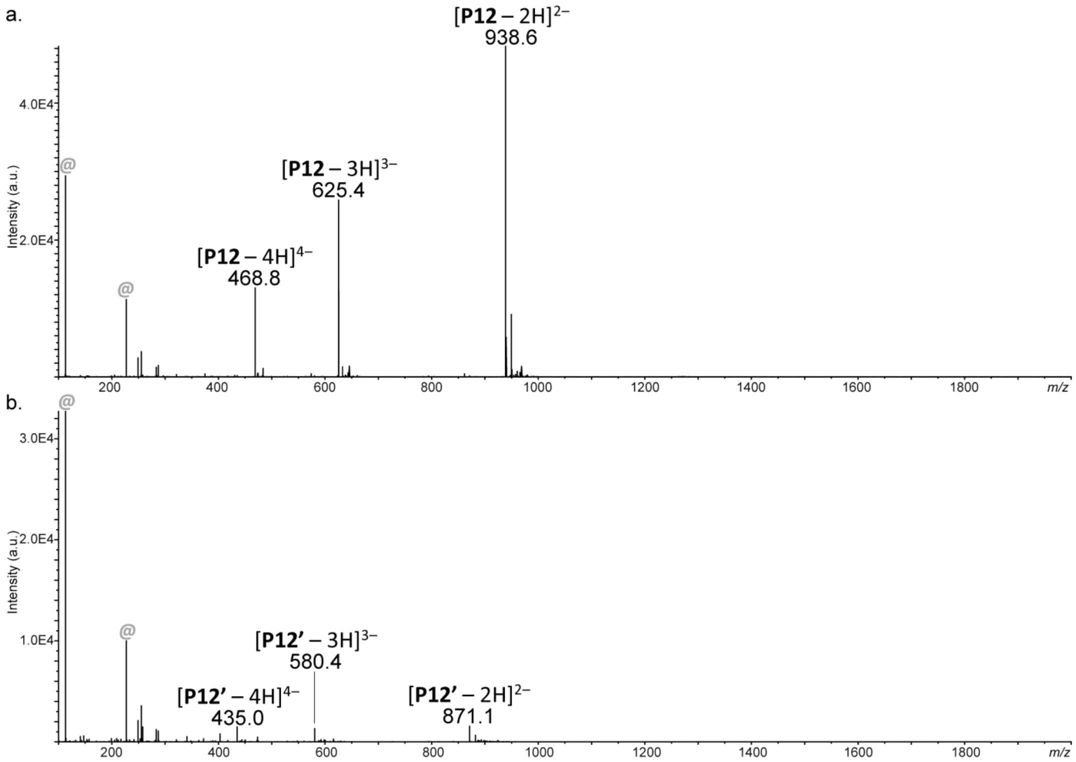

Supplementary Figure 49. ESI HRMS spectra recorded for (**a**) copolymer **P12** before irradiation and (**b**) the resulting polymer **P12'** after photo-exposure. Grey symbols indicate trifluoroacetic acid clusters (@).

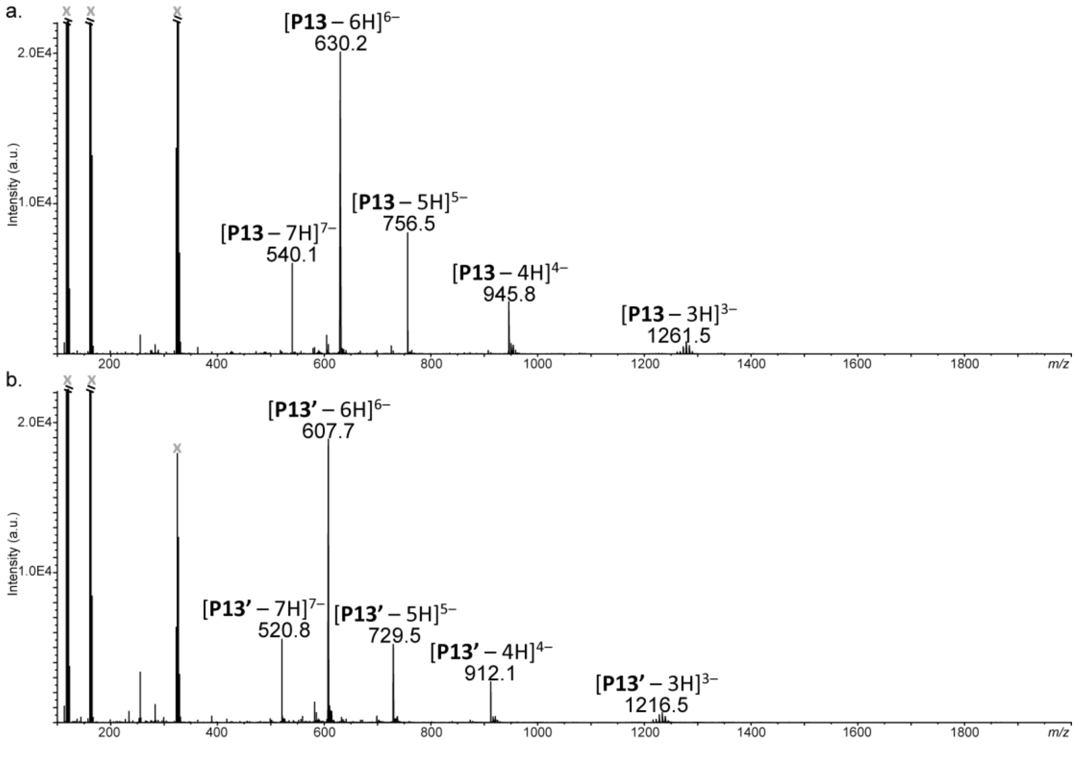

Supplementary Figure 50. ESI HRMS spectra recorded for (**a**) copolymer **P13** before irradiation and (**b**) the resulting polymer **P13'** after photo-exposure. Numerous H/Na exchanges in the lowest charge state species account for the more complex pattern observed for the 3– ions. Grey symbols designate clusters of trifluoroacetic acid (x).

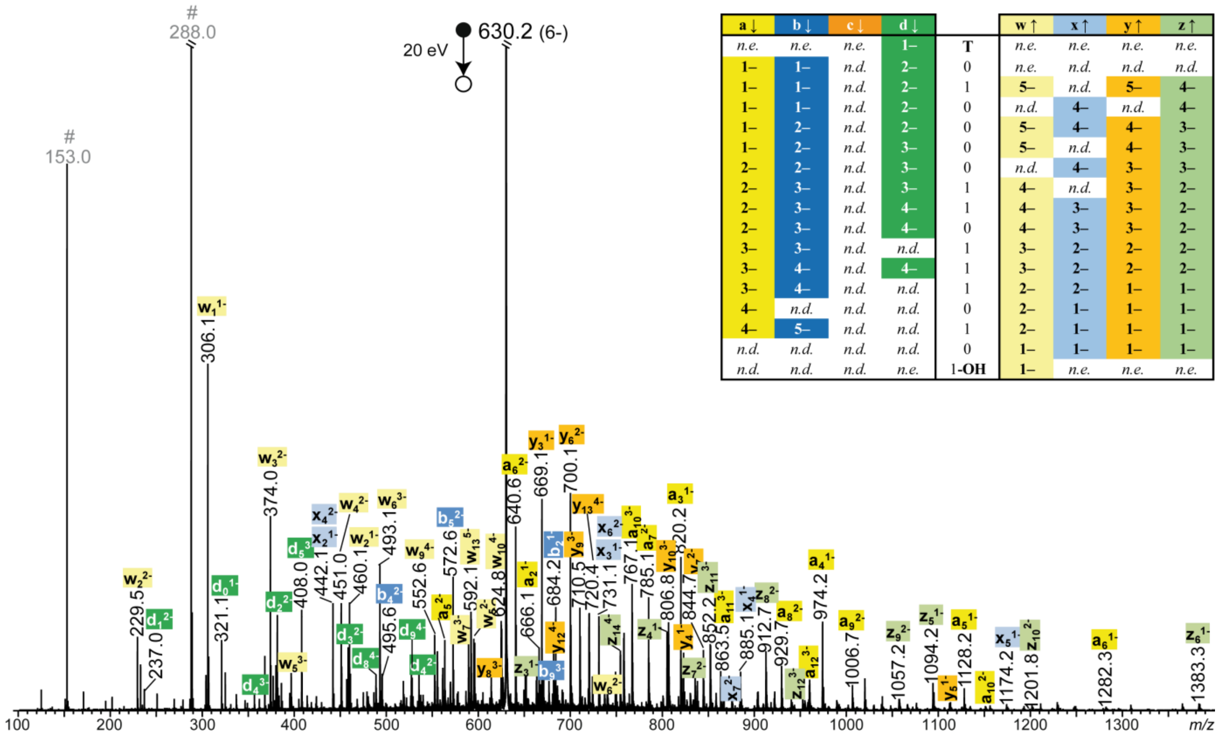

a↓	b↓	c↓	d↓		w↑	x↑	y↑	z↑
n.e.	*n.e.*	*n.e.*	1–	**T**	*n.e.*	*n.e.*	*n.e.*	*n.e.*
1–	1–	*n.d.*	2–	0	*n.e.*	*n.d.*	*n.d.*	*n.d.*
1–	1–	*n.d.*	2–	1	5–	*n.d.*	5–	4–
1–	1–	*n.d.*	2–	0	*n.d.*	4–	*n.d.*	4–
1–	2–	*n.d.*	2–	0	5–	4–	4–	3–
1–	2–	*n.d.*	3–	0	5–	*n.d.*	4–	3–
2–	2–	*n.d.*	3–	0	*n.d.*	4–	3–	3–
2–	3–	*n.d.*	3–	1	4–	*n.d.*	3–	2–
2–	3–	*n.d.*	4–	1	4–	3–	3–	2–
2–	3–	*n.d.*	4–	0	4–	3–	3–	2–
3–	3–	*n.d.*	*n.d.*	1	3–	2–	2–	2–
3–	4–	*n.d.*	4–	1	3–	2–	2–	2–
3–	4–	*n.d.*	*n.d.*	1	2–	2–	1–	1–
4–	*n.d.*	*n.d.*	*n.d.*	0	2–	1–	1–	1–
4–	5–	*n.d.*	*n.d.*	1	2–	1–	1–	1–
n.d.	*n.d.*	*n.d.*	*n.d.*	0	1–	1–	1–	1–
n.d.	*n.d.*	*n.d.*	*n.e.*	1-**OH**	1–	*n.e.*	*n.e.*	*n.e.*

Supplementary Figure 51. MS/MS spectra recorded for copolymer **P13** before irradiation, using a 20 eV collision energy (laboratory frame) to activate the [**P13** – 6H]$^{6-}$ precursor at *m/z* 630.2. In grey: secondary fragments, including the deprotonated units, [0 – H]$^{-}$ at *m/z* 153.0 and [1 – H]$^{-}$ at *m/z* 288.0, both designated by #. Inset Table: Sequence coverage of **P13** (*n.d.*: not detected; *n.e.*: not expected), with detailed assignments reported in Supplementary Table 7.

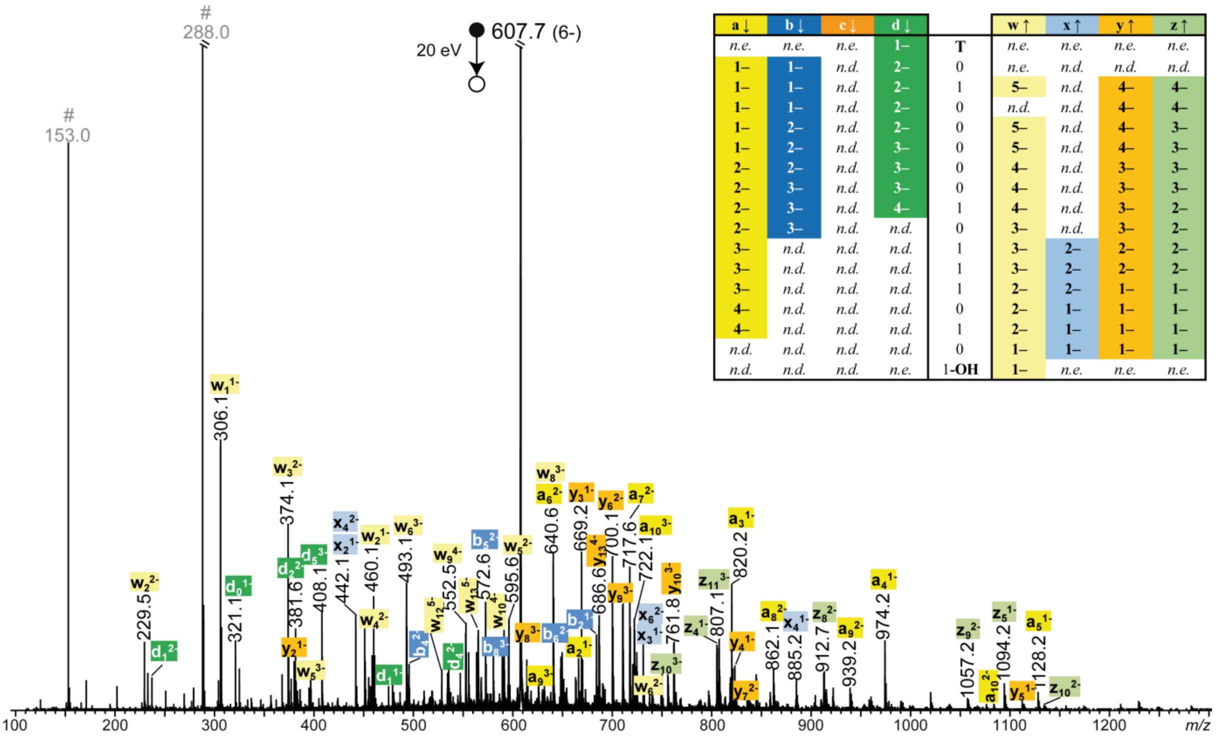

a ↓	b ↓	c ↓	d ↓		w ↑	x ↑	y ↑	z ↑
n.e.	*n.e.*	*n.e.*	1–	**T**	*n.e.*	*n.e.*	*n.e.*	*n.e.*
1–	1–	*n.d.*	2–	0	*n.e.*	*n.d.*	*n.d.*	*n.d.*
1–	1–	*n.d.*	2–	1	5–	*n.d.*	4–	4–
1–	1–	*n.d.*	2–	0	*n.d.*	*n.d.*	4–	4–
1–	2–	*n.d.*	2–	0	5–	*n.d.*	4–	3–
1–	2–	*n.d.*	3–	0	5–	*n.d.*	4–	3–
2–	2–	*n.d.*	3–	0	4–	*n.d.*	3–	3–
2–	3–	*n.d.*	3–	0	4–	*n.d.*	3–	3–
2–	3–	*n.d.*	4–	1	4–	*n.d.*	3–	2–
2–	3–	*n.d.*	*n.d.*	0	3–	*n.d.*	3–	2–
3–	*n.d.*	*n.d.*	*n.d.*	1	3–	2–	2–	2–
3–	*n.d.*	*n.d.*	*n.d.*	1	3–	2–	2–	2–
3–	*n.d.*	*n.d.*	*n.d.*	1	2–	2–	1–	1–
4–	*n.d.*	*n.d.*	*n.d.*	0	2–	1–	1–	1–
4–	*n.d.*	*n.d.*	*n.d.*	1	2–	1–	1–	1–
n.d.	*n.d.*	*n.d.*	*n.d.*	0	1–	1–	1–	1–
n.d.	*n.d.*	*n.d.*	*n.e.*	1-**OH**	1–	*n.e.*	*n.e.*	*n.e.*

Supplementary Figure 52. MS/MS spectra recorded for copolymer **P13’** obtained after irradiation of **P13**, using a 20 eV collision energy (laboratory frame) to activate [**P13’** – 6H]$^{6-}$ at *m/z* 607.7. In grey: secondary fragments, including the deprotonated units, [0 – H]$^{-}$ at *m/z* 153.0 and [1 – H]$^{-}$ at *m/z* 288.0, both designated by #. Inset Table: Sequence coverage of **P13’** (*n.d.*: not detected; *n.e.*: not expected), with detailed assignments reported in Supplementary Table 8.

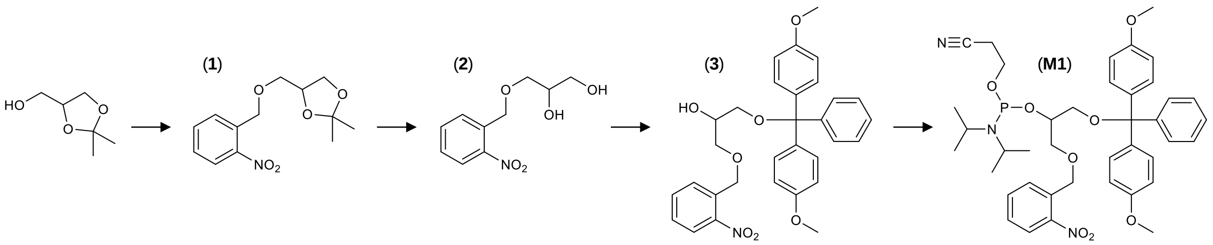

Supplementary Figure 53. Synthetic route used in this work for the preparation of **M1**.

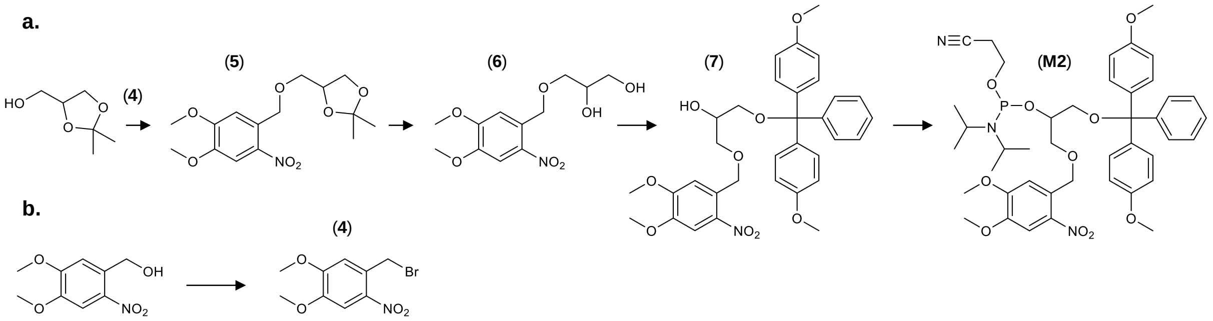

Supplementary Figure 54. Synthetic route used in this work for the preparation of **M2**: (**a**) main synthesis route; (**b**) synthesis of 4,5-dimethoxy-2-nitrobenzyl bromide.

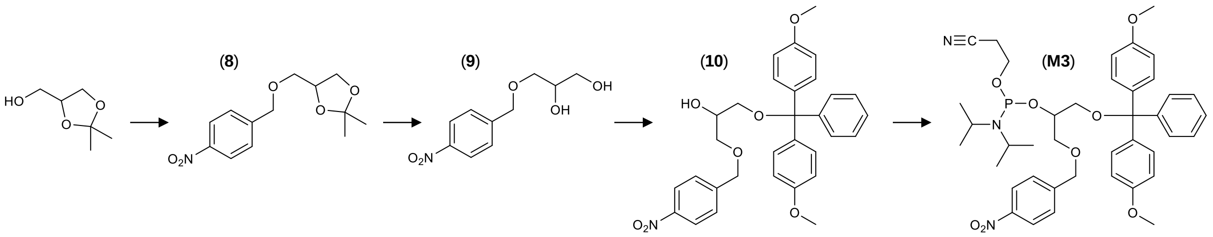

Supplementary Figure 55. Synthetic route used in this work for the preparation of **M3**.

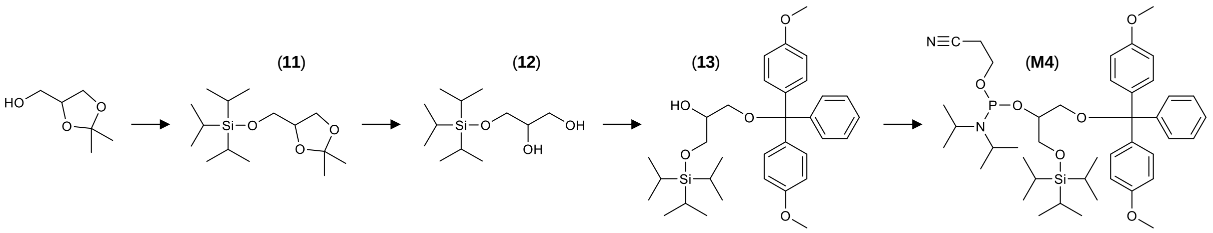

Supplementary Figure 56. Synthetic route used in this work for the preparation of **M4**.

Supplementary Tables.

Supplementary Table 1. ESI-HRMS characterization of photo-erasable polymers.

Before irradiation				After irradiation			
	Sequence[a]	m/z_{th}[b]	m/z_{exp}		Sequence[a]	m/z_{th}[b]	m/z_{exp}[c]
**H1**	T 00000000	850.4499	850.4451	**H1'**	T ••••••••	490.3644	490.3584
**H2**	T 11111111	1010.5062	1010.5081	**H2'**	T ••••••••	490.3644	*n.d.*[d]
**P1**	T 01000101	910.4710	910.4710	**P1'**	T ••••••••	490.3644	490.3555
**P2**	T 01110010	930.4780	930.4785	**P2'**	T ••••••••	490.3644	490.3559
**P3**	T 01100001	910.4710	910.4711	**P3'**	T ••••••••	490.3644	490.3589
**P4**	T 01110011	950.4851	950.4839	**P4'**	T ••••••••	490.3644	490.3585
**P5**	T 01100101	930.4780	930.4795	**P5'**	T ••••••••	490.3644	490.3573

[a] Before irradiation, bits 0 and 1 correspond to monomers **M1** and **M2**, respectively. After irradiation, the symbol • represents the monomer unit of a non-decodable homopolymer. [b] The displayed *m/z* values correspond to $[M-3H]^{3-}$. [c] After irradiation, targeted species were of too low abundance to allow high accuracy mass measurements, accounting for relative error of 10-20 ppm. [d] Due to its very low abundance, the triply deprotonated **H2'** polymer could not be accurately mass measured (*n.d.*: not determined).

Supplementary Table 2. ESI-HRMS characterization of polymers coded with an invisible ink.

Before irradiation				After irradiation			
	Sequence[a]	m/z_{th}[b]	m/z_{exp}		Sequence[a]	m/z_{th}[b]	m/z_{exp}
**H3**	T 11111111	850.4499	850.4520	**H3'**	T 11111111	850.4499	850.4484
**P6**	T **1**1**111**1**1**1	850.4499	850.4504	**P6'**	T 01000101	625.3965	625.3934
**P7**	T **1**1111**111**	850.4499	850.4527	**P7'**	T 01111000	670.4072	670.4061
**P8**	T **1**111**1111**	850.4499	850.4489	**P8'**	T 01110000	625.3965	625.3924
**P9**	T **1**11**1**1111	850.4499	850.4538	**P9'**	T 01101111	760.4285	760.4254
**P10**	T **1**111**11**11	850.4499	850.4537	**P10'**	T 01110011	715.4178	715.4148
**P11**	T **1**11**11**1**1**1	850.4499	850.4522	**P11'**	T 01100101	670.4072	670.4073

[a] Before irradiation, **1** and 1 correspond to isobaric monomers **M1** and **M3**, respectively. After irradiation, bit 0 corresponds to a photo-deprotected **M1** unit. [b] The displayed *m/z* values correspond to $[M-3H]^{3-}$.

Supplementary Table 3. ESI-HRMS characterization of photo-mutable polymers.

Before irradiation				After irradiation			
	Sequence[a]	m/z_{th}	m/z_{exp}		Sequence[a]	m/z_{th}	m/z_{exp}
**P12**	T 0100001**1**	938.5983[b]	938.5995	**P12'**	T 01000010	871.0823[b]	871.0818
**P13**	T 010000**1**101110101	756.4726[c]	756.4720	**P13'**	T 0100000101110101	729.4662[c]	729.4660

[a] Before irradiation, **1** and 1 correspond to isobaric monomers **M1** and **M3**, respectively. Bit 0 corresponds to an OH-functional unit. [b] The displayed *m/z* values correspond to $[M-2H]^{2-}$. [c] The displayed *m/z* values correspond to $[M-5H]^{5-}$, measured at isotopic maximum.

Supplementary Table 4. Full coverage of the T01000101 sequence of **P1** using MS/MS data shown in Figure 2c.

**a ↓**	**b ↓**	**c ↓**	**d ↓**		**w ↑**	**x ↑**	**y ↑**	**z ↑**
n.e.	*n.e.*	1–	1–	**T**	*n.e.*	*n.e.*	*n.e.*	*n.e.*
1–	1–	1–	2–	0	*n.e.*	*n.d.*	2–	*n.d.*
1–	1–	*n.d.*	2–	1	2–/3–	3–	2–	2–
1–	2–	*n.d.*	*n.d.*	0	2–/3–	*n.d.*	2–	2–
1–	2–	*n.d.*	3–	0	2–/3–	2–	1–/2–	1–
1–/2–	2–	*n.d.*	*n.d.*	0	2–	*n.d.*	1–	1–
2–	2–/3–	*n.d.*	3–/4–	1	1–/2–	1–	1–	1–
2–/3–	*n.d.*	*n.d.*	*n.e.*	0	1–/2–	1–	1–	*n.d.*
n.e.	*n.e.*	*n.e.*	*n.e.*	1-**OH**	1–	*n.e.*	*n.e.*	*n.e.*

Values indicate the fragment charge state. *n.d.*: not detected. *n.e.*: not expected.

Supplementary Table 5. Full coverage of the T01110011 sequence of **P10'** using MS/MS data shown in Figure 3c.

**a ↓**	**b ↓**	**c ↓**	**d ↓**		**w ↑**	**x ↑**	**y ↑**	**z ↑**
n.e.	*n.e.*	1–	1–	**T**	*n.e.*	*n.e.*	*n.e.*	*n.e.*
1–	1–	1–	1–	0	*n.e.*	3–	*n.d.*	2–
1–	1–	1–	1–	1	3–	2–	2–	2–
1–	1–	1–	2–	1	2–	2–	2–	1–
1–	1–	2–	2–	1	2–	1–	1–	1–
1–	2–	2–	2–	0	2–	1–	1–	1–
2–	2–	2–	3–	0	1–	1–	1–	1–
2–	2–	3–	*n.e.*	1	1–	1–	1–	1–
n.e.	*n.e.*	*n.e.*	*n.e.*	1-**OH**	1–	*n.e.*	*n.e.*	*n.e.*

Values indicate the fragment charge state. *n.d.*: not detected. *n.e.*: not expected.

Supplementary Table 6. MS/MS data allowing full coverage of the T0100001X sequence of **P12** (X = 1) and **P12'** (X = 0).[a]

	**T**	**0**	**1**	**0**	**0**	**0**	**0**	**1**	**X**	
**i→**	*0*	*1*	*2*	*3*	*4*	*5*	*6*	*7*	*8*	
$\mathbf{a_i^{z-}}$	*n.e.*	*n.d.*	666.1^{1-}	820.2^{1-}	974.2^{1-}	1128.3^{1-}	1282.3^{1-}	785.2^{2-}	*n.e.*	
$\mathbf{b_i^{z-}}$	*n.e.*	395.1^{1-}	684.1^{1-}	838.2^{1-}	992.2^{1-}	1146.3^{1-}	1300.3^{1-}	794.2^{2-}	*n.e.*	
$\mathbf{c_i^{z-}}$	*n.e.*	457.1^{1-}	746.1^{1-}	900.2^{1-}	1054.2^{1-}	1208.2^{1-}	680.6^{2-}	*n.d.*	*n.e.*	
$\mathbf{d_i^{z-}}$	321.1^{1-}	475.1^{1-}	764.1^{1-}	918.2^{1-}	1072.2^{1-}	1226.2^{1-}	689.6^{2-}	834.5^{2-}	*n.e.*	
		8	*7*	*6*	*5*	*4*	*3*	*2*	*1*	**←j**
	n.e.	*n.e.*	749.6^{2-}	1211.2^{1-}	1057.2^{1-}	903.2^{1-}	749.1^{1-}	595.1^{1-}	306.1^{1-}	$\mathbf{w_j^{z-}}$
	n.e.	*n.e.*	682.1^{2-}	1076.2^{1-}	922.1^{1-}	768.1^{1-}	614.1^{1-}	460.1^{1-}	171.1^{1-}	
	n.e.	817.6^{2-}	1482.2^{1-}	1193.2^{1-}	1039.2^{1-}	885.2^{1-}	731.1^{1-}	577.1^{1-}	*n.e.*	$\mathbf{x_j^{z-}}$
	n.e.	750.1^{2-}	673.1^{2-}	1058.1^{1-}	904.1^{1-}	750.1^{1-}	596.1^{1-}	442.1^{1-}	*n.e.*	
	n.e.	*n.d.*	1420.2^{1-}	1131.3^{1-}	977.2^{1-}	823.2^{1-}	669.1^{1-}	515.1^{1-}	*n.e.*	$\mathbf{y_j^{z-}}$
	n.e.	719.1^{2-}	1285.3^{1-}	996.2^{1-}	842.2^{1-}	688.1^{1-}	534.1^{1-}	380.1^{1-}	*n.e.*	
	n.e.	777.6^{2-}	1402.3^{1-}	1113.3^{1-}	959.2^{1-}	805.2^{1-}	651.1^{1-}	497.1^{1-}	*n.e.*	$\mathbf{z_j^{z-}}$
	n.e.	710.1^{2-}	1267.3^{1-}	978.1^{1-}	824.2^{1-}	670.1^{1-}	516.1^{1-}	362.1^{1-}	*n.e.*	

[a] For both polymers **P12** and **P12'**, α-terminated fragments (a_i, b_i, c_i, d_i) are identical since, in these series, species containing all units including the last X one (a_8, b_8, c_8, d_8) are not expected to form (*n.e.*). In contrast, ω-terminated fragments (w_j, x_j, y_j, z_j) all contain the X unit as this is the first unit when counting from the right- to the left-hand side: accordingly, these series are different when X=1 for **P12** (*m/z* values in black) or X=0 for **P12'** (*m/z* values in red), with a mass shift corresponding to $m_1 - m_0 = 135$ Da.

n.d.: not detected.

Supplementary Table 7. MS/MS data allowing full coverage of the T0100001101110101 sequence of **P13** (*n.e.*: not expected; *n.d.*: not detected).

i↓	a_i^{z-}	b_i^{z-}	c_i^{z-}	d_i^{z-}		w_j^{z-}	x_j^{z-}	y_j^{z-}	z_j^{z-}	
0	*n.e.*	*n.e.*	*n.e.*	321.1^{1-}	**T**	*n.e.*	*n.e.*	*n.e.*	*n.e.*	
1	377.1^{1-}	395.1^{1-}	*n.d.*	237.0^{2-}	**0**	*n.d.*	*n.d.*	*n.d.*	*n.d.*	16
2	666.2^{1-}	684.2^{1-}	*n.d.*	381.5^{2-}	**1**	680.7^{5-}	*n.d.*	664.7^{5-}	826.6^{4-}	15
3	820.2^{1-}	838.2^{1-}	*n.d.*	458.6^{2-}	**0**	*n.d.*	619.3^{4-}	*n.d.*	754.4^{4-}	14
4	974.2^{1-}	495.6^{2-}	*n.d.*	535.6^{2-}	**0**	592.1^{5-}	735.9^{4-}	720.4^{4-}	954.8^{3-}	13
5	1128.2^{1-}	572.6^{2-}	*n.d.*	408.0^{3-}	**0**	561.3^{5-}	*n.d.*	681.9^{4-}	903.5^{3-}	12
6	640.6^{2-}	649.6^{2-}	*n.d.*	459.4^{3-}	**0**	*n.d.*	658.8^{4-}	858.2^{3-}	852.2^{3-}	11
7	785.1^{2-}	529.1^{3-}	*n.d.*	555.6^{3-}	**1**	624.8^{4-}	*n.d.*	806.8^{3-}	1201.8^{2-}	10
8	929.7^{2-}	625.4^{3-}	*n.d.*	488.8^{4-}	**1**	552.6^{4-}	731.1^{3-}	710.5^{3-}	1057.2^{2-}	9
9	1006.7^{2-}	676.8^{3-}	*n.d.*	527.3^{4-}	**0**	480.3^{4-}	634.7^{3-}	614.1^{3-}	912.7^{2-}	8
10	767.1^{3-}	773.1^{3-}	*n.d.*	*n.d.*	**1**	589.4^{3-}	875.6^{2-}	844.7^{2-}	835.7^{2-}	7
11	863.5^{3-}	651.8^{4-}	*n.d.*	537.2^{5-}	**1**	493.1^{3-}	731.1^{2-}	700.1^{2-}	691.1^{2-}	6
12	959.8^{3-}	724.1^{4-}	*n.d.*	*n.d.*	**1**	595.6^{2-}	586.6^{2-}	1112.2^{1-}	1094.2^{1-}	5
13	758.1^{4-}	*n.d.*	*n.d.*	*n.d.*	**0**	451.0^{2-}	885.1^{1-}	823.2^{1-}	805.2^{1-}	4
14	830.4^{4-}	667.6^{5-}	*n.d.*	*n.d.*	**1**	374.0^{2-}	731.1^{1-}	669.2^{1-}	651.1^{1-}	3
15	*n.d.*	*n.d.*	*n.d.*	*n.d.*	**0**	460.1^{1-}	442.1^{1-}	380.1^{1-}	362.1^{1-}	2
16	*n.d.*	*n.d.*	*n.d.*	*n.e.*	**1-OH**	306.1^{1-}	*n.e.*	*n.e.*	*n.e.*	1
										j↑

Supplementary Table 8. MS/MS data allowing full coverage of the T0100000101110101 sequence of **P13’** (*n.e.*: not expected; *n.d.*: not detected).

i↓	a_i^{z-}	b_i^{z-}	c_i^{z-}	d_i^{z-}		w_j^{z-}	x_j^{z-}	y_j^{z-}	z_j^{z-}	
0	*n.e.*	*n.e.*	*n.e.*	321.1^{1-}	**T**	*n.e.*	*n.e.*	*n.e.*	*n.e.*	
1	377.1^{1-}	395.1^{1-}	*n.d.*	237.0^{2-}	**0**	*n.d.*	*n.d.*	*n.d.*	*n.d.*	16
2	666.2^{1-}	684.2^{1-}	*n.d.*	381.5^{2-}	**1**	653.7^{5-}	*n.d.*	797.4^{4-}	792.9^{4-}	15
3	820.2^{1-}	838.2^{1-}	*n.d.*	458.6^{2-}	**0**	*n.d.*	*n.d.*	725.1^{4-}	720.6^{4-}	14
4	974.2^{1-}	495.6^{2-}	*n.d.*	535.6^{2-}	**0**	565.1^{5-}	*n.d.*	686.6^{4-}	909.8^{3-}	13
5	1128.2^{1-}	572.6^{2-}	*n.d.*	408.0^{3-}	**0**	534.2^{5-}	*n.d.*	648.1^{4-}	858.5^{3-}	12
6	640.6^{2-}	649.6^{2-}	*n.d.*	459.4^{3-}	**0**	629.6^{4-}.	*n.d.*	813.1^{3-}	807.1^{3-}	11
7	717.6^{2-}	484.3^{3-}	*n.d.*	510.7^{3-}	**0**	691.1^{4-}	*n.d.*	761.8^{3-}	755.8^{3-}	10
8	862.1^{2-}	580.4^{3-}	*n.d.*	455.1^{4-}	**1**	552.6^{4-}	*n.d.*	710.5^{3-}	1057.2^{2-}	9
9	939.2^{2-}	631.7^{3-}	*n.d.*	*n.d.*	**0**	480.3^{4-}	*n.d.*	614.1^{3-}	912.7^{2-}	8
10	722.1^{3-}	*n.d.*	*n.d.*	*n.d.*	**1**	589.4^{3-}	875.6^{2-}	844.7^{2-}	835.5^{2-}	7
11	818.5^{3-}	*n.d.*	*n.d.*	*n.d.*	**1**	493.1^{3-}	731.1^{2-}	700.1^{2-}	691.1^{2-}	6
12	914.8^{3-}	*n.d.*	*n.d.*	*n.d.*	**1**	595.6^{2-}	586.6^{2-}	1112.3^{1-}	1094.2^{1-}	5
13	724.4^{4-}	*n.d.*	*n.d.*	*n.d.*	**0**	451.0^{2-}	885.1^{1-}	823.2^{1-}	805.2^{1-}	4
14	796.6^{4-}	*n.d.*	*n.d.*	*n.d.*	**1**	374.0^{2-}	731.1^{1-}	669.2^{1-}	651.1^{1-}	3
15	*n.d.*	*n.d.*	*n.d.*	*n.d.*	**0**	460.1^{1-}	442.1^{1-}	380.1^{1-}	362.1^{1-}	2
16	*n.d.*	*n.d.*	*n.d.*	*n.e.*	**1-OH**	306.1^{1-}	*n.e.*	*n.e.*	*n.e.*	1
										j↑

Supplementary References.

1 Klán, P., Šolomek, T., Bochet, C. G., Blanc, A., Givens, R., Rubina, M., Popik, V., Kostikov, A. & Wirz, J. Photoremovable Protecting Groups in Chemistry and Biology: Reaction Mechanisms and Efficacy. *Chem. Rev.* **113**, 119-191 (2013).

2 Barltrop, J. A., Plant, P. J. & Schofield, P. Photosensitive protective groups. *Chem. Commun.*, 822-823 (1966).

3 Patchornik, A., Amit, B. & Woodward, R. B. Photosensitive protecting groups. *J. Am. Chem. Soc.* **92**, 6333-6335 (1970).

4 Carré, P. Sur la décomposition de l'alcool ortho-nitrobenzylique sous l'influence de la soude aqueuse, et de la soude alcoolique. *C.R. Hebd. Seances Acad. Sci.* **140**, 663-665 (1905).
